# Randomised feasibility trial of a remotely delivered holistic UK employee programme combining tailored sleep hygiene, diet, and physical activity counselling for weight management: a mixed-methods evaluation

**DOI:** 10.1017/jns.2026.10104

**Published:** 2026-06-18

**Authors:** Andrea Du Preez, Danae Marshall, Lorraine Kelly, Kirti Swift, Zak Evans, Michael Clinton, Rakhee Doshi, Charlotte Fitzhugh, Benjamin Gardner, Rachel Gibson, Wendy L. Hall

**Affiliations:** 1 Department of Nutritional Sciences, King’s College London, UK; 2 Organisational Development, King’s College London, UK; 3 Department of Human Resource Management & Employment Relations, King’s College London, UK; 4 Institute of Psychiatry, Psychology & Neuroscience, King’s College London, London, UK; 5 Research Institute for Sport and Exercise Sciences, Liverpool John Moores University, Liverpool, UK; 6 School of Psychological Sciences, University of Surrey, UK

**Keywords:** Diet, Feasibility trial, Holistic, Lifestyle intervention, Physical activity, Sleep, Weight management

## Abstract

Workplaces offer a key setting for health improvement interventions, given their reach and potential to promote lasting behaviour change. While diet and physical activity (PA) are common targets, sleep remains an underexplored yet influential factor in employee health. This randomised feasibility trial evaluated the practicality and acceptability of a 14-week, remotely delivered sleep-enhanced intervention (SEI) integrating sleep, PA, and diet, compared with a standard intervention (SI) focusing on PA and diet only, with exploratory assessment of behavioural and health-related outcomes. Staff from a UK higher education institution (*n* = 28) with short sleep duration (<7 h) and elevated cardiometabolic risk were randomised to SEI or SI. Quantitative data included self-reported and objective measures of PA, sleep, diet, and anthropometry over 14 weeks. Feasibility outcomes (recruitment, retention, compliance) and acceptability were assessed through qualitative interviews informed by the Theoretical Framework of Acceptability (TFA). Recruitment targets were achieved with 82% retention and 84% adherence. The SEI group showed indications of improvement in PA, dietary behaviours, anxiety symptoms, and sleep hygiene, while both groups showed reductions in BMI and waist circumference. Qualitative data highlighted the value of personalised guidance, peer support, and accountability, but also noted that the SEI’s complexity sometimes hindered adherence. The WHOLE trial demonstrates the feasibility and acceptability of a remotely delivered, holistic lifestyle intervention for UK employees. A phased or extended approach may enhance sustainability and engagement in future large-scale evaluations.

## Introduction

Obesity contributes to 7.7% of global deaths and has been estimated to cost the UK economy up to £97 billion annually.^(1–3)^ Poor sleep, unhealthy diets, and low physical activity (PA), defined as not achieving the recommended 150 min of moderate-intensity activity per week^(4)^ are all key, interconnected factors linked to obesity.^(5–10)^ Sleep disruption can promote unhealthy eating by increasing snacking opportunities, disrupting hormonal regulation, and altering brain reward pathways.^(11–14)^ Meanwhile, sedentary lifestyles can lead to increased energy intake and weight gain, further exacerbating health issues.^(15)^ Although diet and PA have long been the focus of obesity prevention efforts, sleep’s role has often been under-recognised. Emerging evidence highlights the need to address sleep, diet, and PA together, given their complex interactions affecting energy balance and health.^(16)^ However, few interventions take this integrated approach, with most excluding sleep, emphasising the need for comprehensive strategies targeting *all three* behaviours simultaneously.

There is a current drive in the UK to improve the health of working adults,^(17)^ and with more than one in ten hours during our lifetime being spent at work,^(18)^ the workplace represents a critical setting for public health interventions. Given the substantial amount of time individuals devote to work, its influence extends not only to behaviours and health outcomes during working hours but also beyond them, shaping broader lifestyle patterns. The work environment can influence the physical, mental, economic, and social well-being of employees, their families and society.^(19)^ Work-related behaviours such as prolonged sitting, poor dietary practices, and inadequate sleep can contribute to weight gain, decreased PA, and overall health decline.^(20–24)^ Digitalisation has further reduced occupational energy expenditure by ∼100 kcal/day,^(25)^ while long, irregular hours continue to exacerbate these issues – ultimately affecting weight management and well-being both in and outside the workforce.^(22,26–29)^

Importantly, the COVID-19 pandemic brought about a significant shift in working practices in the UK, with many desk-based employers adopting a work-from-home model. Although the prevalence of remote work has declined since 2022, it is estimated that about 20% of UK adults continue to work remotely or in a hybrid pattern.^(30,31)^ This shift may impact health behaviours, as lack of traditional workplace structure can lead to more sedentary time, irregular meals, and blurred work–rest boundaries, harming health and well-being.^(32,33)^ Therefore, effective employee health interventions must reflect the changing nature of work.

The UK government has implemented various strategies (e.g. Better Health campaign) to reduce the prevalence of obesity, including workplace interventions, fiscal policies, and initiatives promoting healthier routines.^(16,34,35)^ An extensive systematic review demonstrated that workplace wellness programmes including a dietary component can have a beneficial impact on markers of cardiometabolic health.^(36)^ However, of the 121 studies included, only one was conducted in the UK. Moreover, while these programmes often combined diet and PA, very few incorporated sleep interventions, meaning there is limited understanding of the potential benefits of integrating sleep improvement strategies. Additionally, there remains a gap in knowledge about how employee wellness interventions can effectively support remote and hybrid workers.^(28)^

Therefore, this study aimed to evaluate the feasibility of a multi-behaviour intervention integrating sleep hygiene – behavioural and environmental practices that promote healthy sleep, such as maintaining regular sleep schedules and optimising the sleep environment – alongside diet and PA to support weight management among remote, desk-based UK employees, addressing a critical evidence gap of growing interest to both employers and policymakers. Developed by a multidisciplinary team, the King’s Workplace Holistic Optimisation of Lifestyle and Energy (King’s-WHOLE) Study sought to enhance sleep quality and duration, increase PA, and improve dietary practices among employees reporting inadequate sleep (<7 h) and a BMI indicative of increased cardiometabolic risk. A mixed-methods process evaluation was conducted to assess both the intervention’s practicality and acceptability, as well as the feasibility of the planned trial procedures, while exploring how the intervention operates, identifying its most promising components, and examining preliminary behavioural trends to inform a future definitive trial.

## Methods and materials

### Study design and setting

This 14-week feasibility randomised controlled trial compared the WHOLE intervention with a control group receiving only counselling on PA and diet (i.e. standard intervention; SI). The intervention was delivered remotely by a UK Registered Associate Nutritionist from 8th March to 6th September 2022. Stopping guidelines were established for serious adverse events, recruitment or retention issues, and operational challenges. These guidelines were not invoked during the trial. The primary outcomes of this feasibility trial were intervention practicality and acceptability, assessed through recruitment, retention, and compliance metrics, alongside qualitative evaluation informed by the Theoretical Framework of Acceptability (TFA).

### Approval

The trial was approved by King’s College London (KCL) Ethics (HR/DP-21/22-2642) and registered on Clinicaltrials.gov (NCT05273892; https://clinicaltrials.gov/study/NCT05273892; registration March 2022). Informed consent was obtained online. The TIDieR guidelines and the CONSORT 2010 extension for feasibility trials guidelines were used to report the study^(37,38)^.

### Study population

The trial recruited desk-based hybrid employees aged 18–70 years from a London-based UK higher education institution (King’s College London). Participants reporting typical sleep duration of 5–7 h per night were included, as <7 h is widely considered insufficient for optimal adult health and is associated with increased cardiometabolic risk.^(39,40)^ A lower threshold of ≥5 h was selected pragmatically to capture short sleepers while reducing inclusion of very short sleepers (<5 h), who have substantially higher health risk and may represent more severe sleep restriction.^(41,42)^ Additional inclusion criteria required employees to work at least 0.5 full-time equivalent, have a BMI ≥ 25 kg/m^2^, and a waist circumference indicative of increased cardiometabolic risk according to the National Institute for Health and Care Excellence (NICE) guidelines for different ethnic groups by sex.^(43)^ Exclusion criteria included current enrolment in a weight management programme, inability to increase PA, diagnosed sleep conditions, or caregiving responsibilities preventing changes to sleep behaviours. Participants were also required to own a smartphone and be technology literate (e.g. able to use online learning platforms, respond to emails, and/or text messages).

### Recruitment

The trial was advertised through newsletters, posters, and a staff well-being survey, with eligibility assessed via an online questionnaire. To achieve the recommended pilot sample size of 12 participants per arm of the trial,^(44)^ and accounting for an estimated 20% attrition rate – consistent with commonly reported loss to follow-up in behavioural lifestyle interventions and pilot trials^(45,46)^ – the target recruitment was 28 participants. No monetary incentives were given, but participants received a scale and tape measure for weekly self-assessment, which they kept after the trial.

### Randomisation and blinding

Groups of three participants (to facilitate group consultation sessions) were randomly assigned to either the SI or sleep-enhanced intervention (SEI) arm following a one-week baseline period. Allocation was performed using opaque sealed envelopes by an independent researcher. Participants were informed only that the study evaluated a remote workplace well-being intervention. If participants reported illness before or during the baseline period, they were reassigned to a group with a later start date.

Given that participants were recruited from a single workplace, there was potential for cross-group contamination through informal interaction among colleagues. To minimise this risk, allocation was concealed and all intervention delivery, including one-to-one consultations and group sessions, was conducted separately for each intervention arm. Participants were not informed of the specific differences between conditions and were asked not to share programme materials. However, informal communication outside study activities could not be fully prevented.

### Trial intervention and measures

**Figure 1.**
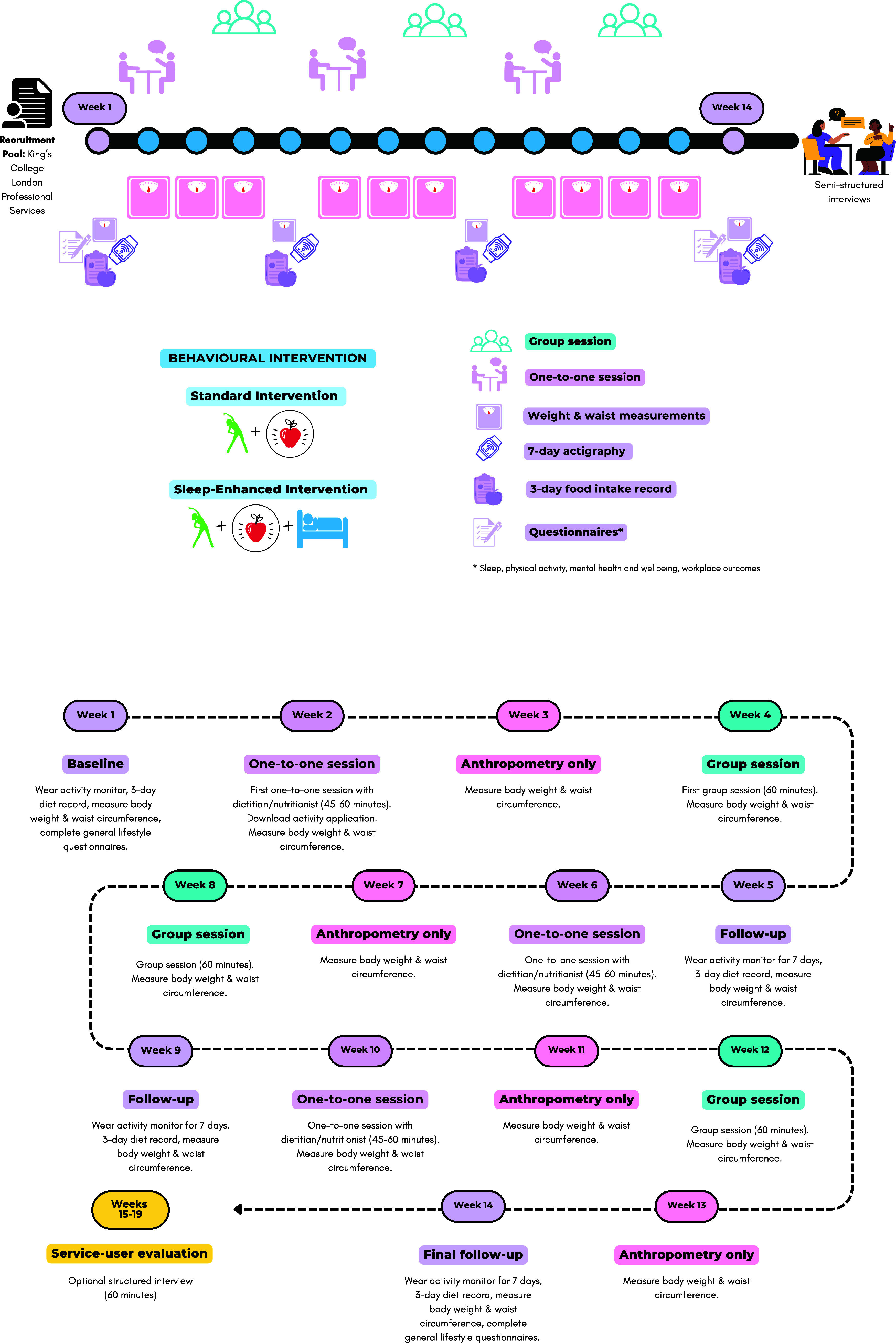
Study flowchart: timeline, measures, and participant progression. King’s College London desk-based employees were invited to participate in a remotely delivered feasibility randomised controlled trial (WHOLE Feasibility Trial). Eligible participants were randomised to either the standard intervention arm, which included diet and physical activity counselling, or the sleep-enhanced intervention arm, which included diet, physical activity, and sleep counselling. At baseline (week 1) and endpoint (week 14), all participants completed a demographics questionnaire, wore an activity watch for seven days to track sleep and physical activity, recorded food intake across three days using the Intake24 online platform, measured their weight and waist circumference, and completed questionnaires assessing lifestyle, mental well-being, and workplace experience. Lifestyle assessments included chronotype (Automated Morningness–Eveningness Questionnaire [AutoMEQ]), physical activity (International Physical Activity Questionnaire – Short Form [IPAQ-SF]), sleep outcomes (Patient Reported Outcomes Measurement Information System [PROMIS]; Epworth Sleepiness Scale [ESS]; Sleep Hygiene Index [SHI]), and alcohol use (Alcohol Use Disorders Identification Test [AUDIT]). Mental well-being was assessed using the Patient Health Questionnaire (PHQ-9) and the Generalised Anxiety Disorder Questionnaire (GAD-7). Workplace experience was evaluated with the Short Utrecht Work Engagement Scale (UWES-9), the Short Index of Job Satisfaction (SIJS), the State Self-Control Capacity Questionnaire (SSCCS), and the Oldenburg Burnout Inventory (OLBI). During the intervention, participants received individual online counselling sessions with an Associate Nutritionist at weeks 2, 6, and 10, which lasted 45 to 60 min and focused on topics assigned per intervention group. Group counselling sessions, also led by the Associate Nutritionist, took place at weeks 4, 8, and 12 and lasted 60 min. Throughout the feasibility trial, participants were asked to measure their weight and waist circumference weekly. At weeks 5 and 9, they were additionally asked to wear the activity watch for seven days to track sleep and physical activity and to record food intake across three days using the Intake24 online platform. At the end of the trial, participants were invited to take part in semi-structured interviews to assess the acceptability of the intervention. *Note: Image created using Canva software*.

For an overview of the trial’s timeline, measures, and participant progression, see Figure 1.

#### Intervention

##### Intervention development

The intervention was developed by a multidisciplinary team of academics and practitioners with expertise in nutrition, sleep science, PA, and behaviour change. In line with behaviour change intervention development guidance,^(47)^ it was designed based on a ‘behavioural diagnosis’. Specifically, the team drew on expert opinion and existing evidence from lifestyle interventions targeting diet, PA, and sleep, alongside literature on behavioural barriers and facilitators, to identify capability, opportunity, and motivation determinants of behaviour (COM-B), and to select appropriate techniques to address these.^(47–55)^

The team deemed capability (lacking the psychological resources needed to act) and opportunity (not detecting or seizing upon opportune moments for action) as the primary barriers to changing all three behaviours, and so the intervention prioritised self-regulatory strategies designed to support individuals to act on their motivation. Core behaviour change techniques included setting and reviewing behavioural and outcome goals, action planning, self-monitoring behaviour and outcomes, personalised feedback on behaviour, problem solving, and social support, which are consistently associated with successful behaviour change interventions.^(46,56,57)^

The sleep component focused on sleep hygiene, encouraging modifiable environmental and behavioural practices that promote healthy sleep through self-regulatory techniques such as prompts and cues, and habit formation approaches.^(58,59)^

Practical considerations, including feasibility of remote delivery, participant burden, and compatibility with working schedules, shaped the structure and content of the programme. The intervention was iteratively refined to maximise acceptability and relevance for the target population.

##### One-to-one consultations

Participants were asked to attend three one-to-one consultations (weeks 2, 6, 10) with an Associate Nutritionist (DM). Each session focused on goal-setting and self-monitoring. Consultations were held at times convenient to participants and could be arranged during working hours. Consultations lasted 30–45 min for the SI group, and 45–60 min for the SEI group, which included additional components related to sleep.

During the first consultation, participants set goals for PA and diet (both groups) and sleep (SEI only), based on their current behaviours and what was realistically achievable (Supplementary materials). The initial session followed the Motivation, Action, Prompts (MAP) framework, helping participants identify social support, plan self-monitoring, and set SMART goals, as previously described.^(60,61)^ Sessions were participant-led, with a nutritionist guiding discussions using data from diet, activity, and sleep records to promote adherence to UK PA and healthy eating guidelines. After each session, participants received their goals and relevant British Dietetic Association food factsheets.^(62)^

The SEI group was also supported to improve sleep using sleep hygiene techniques. Participants were educated on the benefits of sleep extension and introduced to a sleep hygiene booklet (Supplementary materials) selecting 4–6 behaviours to implement. They were guided through a process of ‘script elicitation’^(51)^ to review habitual bedtime routines, identify barriers, and design improved alternatives (Supplementary materials), followed by a sleep behaviour contract confirming their new plans.

Subsequent consultations focused on reviewing progress, updating goals, and addressing new challenges. All sessions were fully personalised.

##### Group consultations

Four group sessions were held in weeks 4, 8, and 12, with 10 groups (5 control, 5 intervention). Of the 10 groups formed (eight with three participants, two with two), five participants withdrew or missed sessions due to illness. Consequently, seven groups ended with two participants, and three groups retained all three participants. Sessions were held during working hours and were participant-led. The nutritionist prompted discussions with questions like, ‘What have you found easy/hard?’ and ‘What sleep changes have been challenging?’ The focus was on participants sharing advice and supporting each other.

All individual/group consultation sessions were conducted online via MS Teams.

#### Measures

##### Baseline (week 1) only

Sociodemographic and lifestyle data were collected via an online survey (Qualtrics), including age, sex, ethnicity, parental status, smoking status, dietary patterns, and other health-related information (e.g. menopause status). Participants also completed the Automated Morningness–Eveningness Questionnaire (AutoMEQ^(63)^) to assess chronotype and, in view of the impact of alcohol consumption on sleep quality, the Alcohol Use Disorders Identification Test (AUDIT) to capture alcohol use.^(64)^ For further detail, see Supplementary materials.

##### Baseline and follow-up

At baseline (week 1) and endpoint (week 14), self-reported questionnaires assessed PA, sleep, mental well-being, and workplace-related experiences. Key measures included the International Physical Activity Questionnaire – Short Form (IPAQ-SF^(65)^) for overall PA and sedentary behaviour, the Patient Reported Outcomes Measurement Information System (PROMIS) sleep/wake disturbances scale,^(66)^ the Epworth Sleepiness Scale (ESS**^(^** ^67)^), and the Sleep Hygiene Index (SHI**^(^** ^59)^), for sleep disturbance, daytime sleepiness, and sleep hygiene, respectively, and the Generalised Anxiety Disorder Questionnaire (GAD-7^(68)^) and Patient Health Questionnaire (PHQ-9^(69)^) for anxiety and depressive symptoms. Workplace-related experiences were measured using the Short Utrecht Work Engagement Scale (UWES-9^(70)^), the Short Index of Job Satisfaction (SIJS^(71)^), the State Self-Control Capacity Scale (SSCCS^(72)^), and the Oldenberg Burnout Inventory (OLBI^(73)^). For further detail, see Supplementary materials.

##### Baseline, mid-trial, and follow-up

Objective measures of PA, sleep, and self-reported dietary intake were collected at four intervals: week 1 (baseline), week 5, week 9, and week 14 (endpoint). Anthropometric measures were recorded weekly from baseline to endpoint.

#### Physical activity

Participants wore a Motion Watch (MW) 8 device (CamNtech Ltd., Cambridgeshire, UK) on their non-dominant wrist for seven consecutive days at each assessment interval. Overall, PA was represented by metabolic equivalent of task (MET) minutes, calculated by multiplying activity duration (in minutes) by its MET value, with total MET minutes summed across all activities.^(74)^

#### Sleep activity

Sleep was also assessed using the MW8 for seven consecutive days at each assessment interval. A composite sleep quality score was generated by averaging standardised sleep duration, efficiency, and fragmentation scores across the whole week, with the fragmentation score multiplied by −1.^(75,76)^ Higher scores indicated better sleep quality. Additional sleep parameters included social jet lag (i.e. the difference between weekday and weekend sleep midpoints, reflecting circadian biological and social misalignment, with lower scores indicating better alignment^(77,78)^) and sleep debt (the cumulative difference between actual sleep duration and the recommended 8 h, with negative values indicating sufficient sleep.^(79)^)

#### Dietary intake

Participants completed four 3-day food records via the Intake24 platform – an online platform used in national UK diet and nutrition surveys (https://intake24.co.uk/) – from which total daily intake (calories), diet quality, and eating behaviours were assessed. Diet quality was evaluated using the Healthy Eating Index (HEI) based on reported intake, with higher scores indicating better diet quality. HEI scores were calculated as previously described^(80)^ and adapted to align with UK dietary guidelines.^(81)^ Additionally, a Nutrient Adequacy Score (NAS) was calculated from nutrient and mineral intake data to reflect the proportion of essential nutrients meeting UK recommendations,^(82)^ where higher scores indicated greater adequacy.^(83)^ Eating behaviour was examined through onset (first energy consumption of the day), offset (last time that energy was consumed), and eating window (total duration between onset and offset).

#### Body weight and waist circumference

Participants measured weight and waist circumference weekly using provided scales and tape measures.

##### Post-completion

Thirteen participants were invited to participate in the optional 1-h semi-structured interviews following trial completion. Conducted via MS Teams at participants’ convenience, interviews followed a researcher-developed schedule covering motivations, expectations, perceived benefits and challenges, and experiences with intervention components (see Supplementary materials). Informed consent was obtained before each interview, which was audio-recorded, transcribed verbatim, and checked for accuracy.

### Primary outcomes

The feasibility of the intervention was evaluated based on two core components:

#### Practicality

Practicality was defined as how easily and effectively the intervention could be implemented. It was assessed using key recruitment, retention, and compliance metrics. Recruitment metrics included recruitment efficiency (participants recruited vs. target), screening response rate (screened vs. interested), recruitment rate (consented vs. interested), eligibility pass rate (eligible vs. screened), and enrolment rate (enrolled vs. eligible). Retention and compliance were assessed via the completion rate (participants who finished the study, regardless of adherence), compliance rate (participants who completed the study and followed the protocol), total retention (participants retained from randomisation to final assessment), post-intervention retention (participants retained from baseline to final assessment), and attrition rate (participants lost to follow-up or withdrawn before study completion).

To measure compliance, participants were expected to complete 14 weekly anthropometric submissions, 7 questionnaires, 4 diet records, wear a MW and complete a sleep diary 4 times, and attend 6 sessions (3 one-to-one, 3 group). Each correctly completed task earned 1 point (max = 35). Partial points (0.5) were awarded for late, early, or incomplete but usable data. Effortful attempts hindered by technical issues were still awarded 1 point. Total compliance included all full and partial points; strict protocol adherence counted only full points.

For full metric definitions, see Supplementary materials.

#### Acceptability

Acceptability was defined as the degree to which participants were satisfied with the intervention, found it relevant to their needs, and were able to adhere to the components of the trial.^(84–86)^ It was assessed both quantitatively and qualitatively to capture key dimensions of,^(84)^ including affective attitude, reflected in participants’ satisfaction and perceived benefits (qualitative interviews); intervention coherence, demonstrated by participants’ understanding of and engagement with intervention components (qualitative data and practicality metrics); burden, assessed through adherence to trial activities and any reported difficulties (qualitative and quantitative data and practicality metrics); perceived effectiveness, explored via participants’ reported outcomes (quantitative data) and satisfaction with changes (qualitative data); and opportunity costs and ethicality, which were indirectly considered through qualitative feedback on barriers and the relevance of the intervention to personal values and needs.

### Data analysis

#### Quantitative data

Quantitative analyses were conducted in this feasibility trial to explore the perceived effectiveness dimension of acceptability and to generate pilot data to inform a future full-scale trial, rather than to confirm definitive intervention effects. As recommended for feasibility studies, analyses focused on estimation rather than hypothesis testing. Effect sizes and CIs are therefore presented to describe the direction and magnitude of observed changes but are not interpreted as confirmatory evidence.

Analyses were conducted using R (v.4.3.1^(87)^). Normality and homoscedasticity were checked, and descriptive statistics were calculated. Outliers were identified, and sensitivity analyses were performed. Baseline characteristics were summarised as means (SD) for continuous variables and percentages for categorical variables by intervention group.

A modified intention-to-treat (mITT) approach (i.e. excluding pre-baseline dropouts; *n* = 4) was implemented, with attrition bias assessed via logistic regression comparing baseline demographics of dropouts and remaining participants.

Outcomes were analysed using linear or generalised mixed-effects models (LMMs/GLMMs), adjusting for repeated measures, baseline scores, and stratification variables (age, sex, ethnicity). The Benjamini–Hochberg procedure and False Discovery Rate correction were applied with an alpha threshold of 0.05.

Missing data were assessed (Supplementary Table 1) and addressed using LMMs/GLMMs with maximum likelihood estimation (MLE/REML), adhering to the mITT principle. Logistic regression models tested whether missing data were missing at random to justify the use of LMM/GLMMs.

For further detail on quantitative analyses, see Supplementary materials.

#### Qualitative data

A qualitative process evaluation explored participants’ experiences, satisfaction, perceived benefits, barriers, and engagement to assess acceptability dimensions of both the trial and intervention and to provide rich contextual insights that support the quantitative findings.

Codebook thematic analysis was conducted on the qualitative data.^(88)^ Interpretation, synthesis, and data reduction were performed independently by three research team members to identify relevant themes. Coding was carried out using NVivo (v.12^(89)^). Codes, grounded in participants’ language, were applied to meaningful units of text and regularly reviewed through team discussions to resolve discrepancies and ensure consistency. These codes were organised into internally coherent themes aligned with study aims. To enhance transparency, a coding manual documented the development of codes and themes (Supplementary materials), and a paper trail illustrated the evolution of the analysis. The iterative process involved re-reading transcripts to treat all data equally and ground findings in the data. Final themes were agreed upon through ongoing discussion and comparison, ensuring a coherent and comprehensive representation of the dataset.

## Results

### Sample characteristics

Table 1 details participant characteristics by intervention group. Baseline demographics were similar across groups, indicating effective randomisation. Participants were predominantly female (82%) with a mean age of 42 ± 12 years. The cohort was diverse, with 50% identifying as ethnic minorities. The mean BMI was 30.4 ± 5.1 kg/m^2^, and 39% had a BMI ≥ 30 kg/m^2^. More participants with a BMI ≥ 30 kg/m^2^ were allocated to the SI group (64%).

**Table 1.** Participant characteristics of all participants enrolled into the WHOLE trial and as stratified by the two intervention groups (*n* = 28)

**Characteristic**	**All participants*N* = 28^a^**	**Standard intervention *N* = 13^a^**	**Sleep-enhanced intervention *N* = 15^a^**
**Sex**			
Female	23 (82)	11 (85)	12 (80)
Male	5 (18)	2 (15)	3 (20)
**Age (years)**	42 (12)	42 (13)	43 (12)
**Ethnicity^b^**			
Asian or Asian British	4 (14)	2 (15)	2 (13)
Black, Black British, Caribbean, or African	3 (11)	1 (8)	2 (13)
Mixed or multiple ethnic groups	1 (4)	0 (0)	1 (7)
White	20 (71)	10 (77)	10 (67)
Other ethnic group	0 (0)	0 (0)	0 (0)
**Parental status**			
Yes	8 (29)	3 (23)	5 (33)
No	20 (71)	10 (77)	10 (67)
**BMI (kg/m²)**	30.4 (5.14)	30.4 (3.92)	30.5 (6.32)
**Waist circumference (cm)**	94 (12.51)	95 (13.21)	93 (12.23)
**Dietary restrictions**			
None	19 (68)	9 (69)	10 (67)
Vegetarian	3 (11)	1(8)	2 (13)
Vegan	2 (7)	1(8)	1 (7)
Pescatarian	1 (4)	1(8)	0 (0)
Gluten free	1 (4)	0 (0)	1 (7)
Flexitarian	1 (4)	1(8)	0 (0)
Not specified	1 (4)	0 (0)	1 (7)
**Chronotype^c^**			
Morning	8 (38)	5 (45)	3 (30)
Intermediate	10 (48)	4 (36)	6 (60)
Evening	3 (14)	2 (18)	1 (10)
**Peri- or post-menopausal status**			
Yes	7 (25)	3 (23)	4 (27)
No	16 (57)	8 (62)	8 (53)
Not applicable	5 (18)	2 (15)	3 (20)
**Current smoking status**			
Yes	2 (7)	2 (13)	0 (0)
No	26 (93)	13 (87)	15 (100)
**Alcohol intake (units per week)**	1.45 (1.68)	1.91 (1.84)	0.90 (1.34)
**Severity of alcohol misuse^d^**			
Low risk	1 (4)	1 (8)	0 (0)
Increasing risk	1 (4)	0 (0)	1 (9)
Higher risk	20 (87)	10 (83)	10 (91)
Possible dependence	1 (4)	1 (8)	0 (0)

a Values represent mean (SD) or *N* (%) of non-missing values from all participants enrolled in the study.

b Based on Office of National Statistics ethnicity categories. Asian or Asian British includes Indian, Pakistani, Bangladeshi, Chinese, and any other Asian background). Black, Black British, Caribbean, or African includes Caribbean, African, and any other Black, Black British, or Caribbean background. Mixed or multiple ethnic groups includes White and Black Caribbean, White and Black African, White and Asian, and any other Mixed or multiple ethnic background. White includes English, Welsh, Scottish, Northern Irish or British, Irish, Gypsy or Irish Traveller, Roma, and any other White background. Other ethnic group includes Arab and any other ethnic group.

c Determined by the self-assessment automated Morningness–Eveningness Questionnaire (AutoMEQ)^(63)^.

d Assessed using the Alcohol Use Disorders Identification Test (AUDIT)^(64)^.

### Demographic characteristics of qualitative interview participants

Thirteen participants (54%) consented to semi-structured interviews, with 62% from the SEI group. The interviewed subgroup (*n* = 13; Supplementary Table 2) had similar demographic characteristics to the wider cohort. Both groups were predominantly female (85%), with 50% identifying as from ethnic minority backgrounds. The interviewed participants had a slightly higher mean age (44 ± 13 years) and BMI (31.3 ± 5.68 kg/m^2^), though overall ranges were comparable.

### Quantitative data

#### Practicality metrics

**Figure 2.**
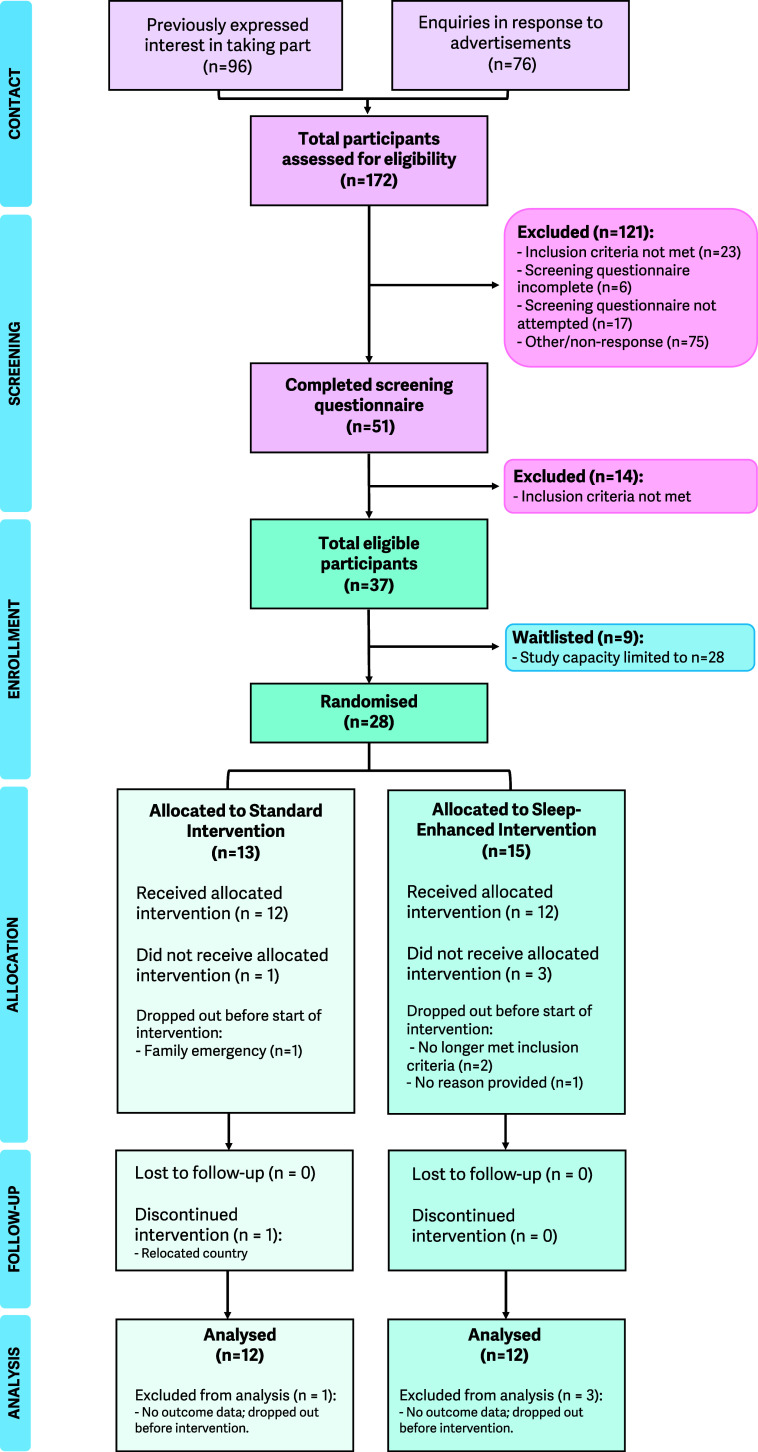
Recruitment and retention of participants. CONSORT diagram showing the flow of participants through each stage of the WHOLE study trial.

**Figure 3.**
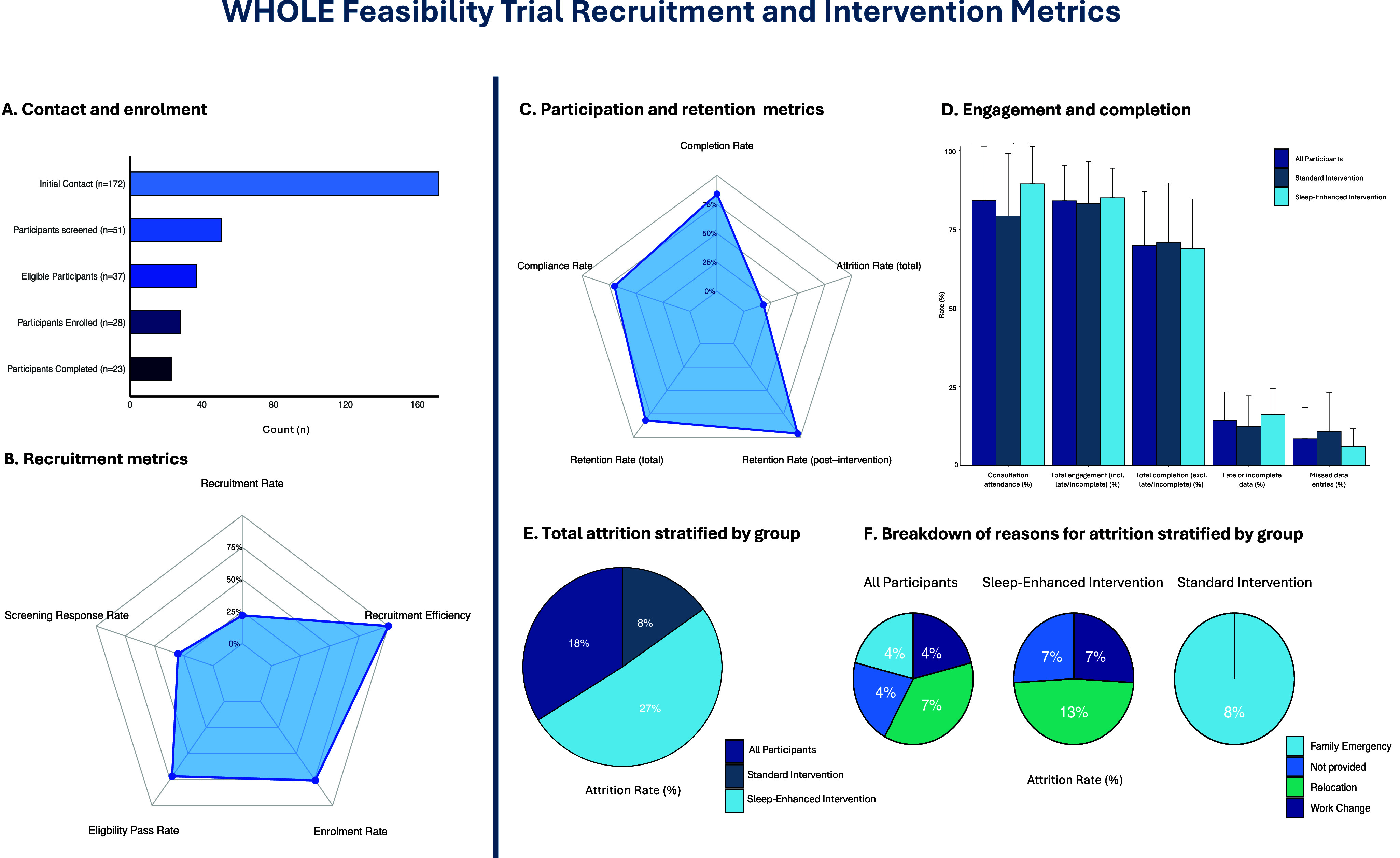
Recruitment, retention, and compliance metrics for the WHOLE feasibility randomised controlled trial. **(a)** Recruitment funnelshowing participant flow from initial contact (*n* = 172) to trial completion (*n* = 23). **(b)** Radar plot of recruitment metrics including efficiency (i.e. the proportion of participants recruited relative to the target sample size), screening response rate (i.e. percentage of individuals who completed the screening questionnaire out of the total number who expressed interest), recruitment rate (i.e. the number of participants who consented to participate relative to the total number of individuals who expressed interest), eligibility pass rate (i.e. the percentage of screened individuals who met the study’s inclusion criteria), and enrolment rate (i.e., theenrolment ratewas the proportion of eligible individuals who proceeded to enrol in the trial). **(c)** Radar plot of retention and compliance metrics, including completion rate (i.e. the percentage of enrolled participants who finished the study, irrespective of whether they fully adhered to the protocol), attrition rate (i.e. the percentage of participants who withdrew or were lost to follow-up before completing the study), total retention rate (i.e. the percentage of participants who remained in the study until the final assessment since randomisation), post-intervention retention rate (i.e. the proportion of participants who stayed in the study until the final assessment, excluding those who dropped out prior to baseline), and compliance rate (i.e. the proportion of participants who finished the study and followed the study protocol as intended). **(d)** Bar chart comparing engagement and completion metricsacross all participants, sleep-enhanced intervention, and standard intervention groups. **(e)** Pie charts illustrating attrition ratesamong all participants and within each intervention group.

As shown in Figures 2 and 3, of 172 screened participants, 37 were eligible, and 28 were randomised, yielding a 22% enrolment rate. Retention was 82% (96% excluding pre-intervention dropouts), with an 84% overall completion rate and 70% full compliance. Attrition was 18%, dropping to 4% when excluding non-starters, primarily due to work changes, family emergencies, or relocation. Retention and compliance were comparable between intervention groups, though the SEI group had higher consultation attendance (89% vs. 79%) but also higher pre-baseline attrition (27% vs. 8%). Once enrolled, no SEI participants dropped out. The SEI group had slightly more late/incomplete data (6% vs. 3%) but fewer missing data overall (2% vs. 4%). Missing data analyses confirmed randomness across all measures, and attrition bias was minimal, with no demographic differences between those who dropped out and those who remained in the trial.

#### Acceptability outcomes

To evaluate the intervention’s relevance, potential benefits, and possible adverse effects as part of acceptability assessment, we quantitatively examined selected behavioural, anthropometric, and psychological outcomes.

##### Physical activity, diet, and sleep

**Figure 4.**
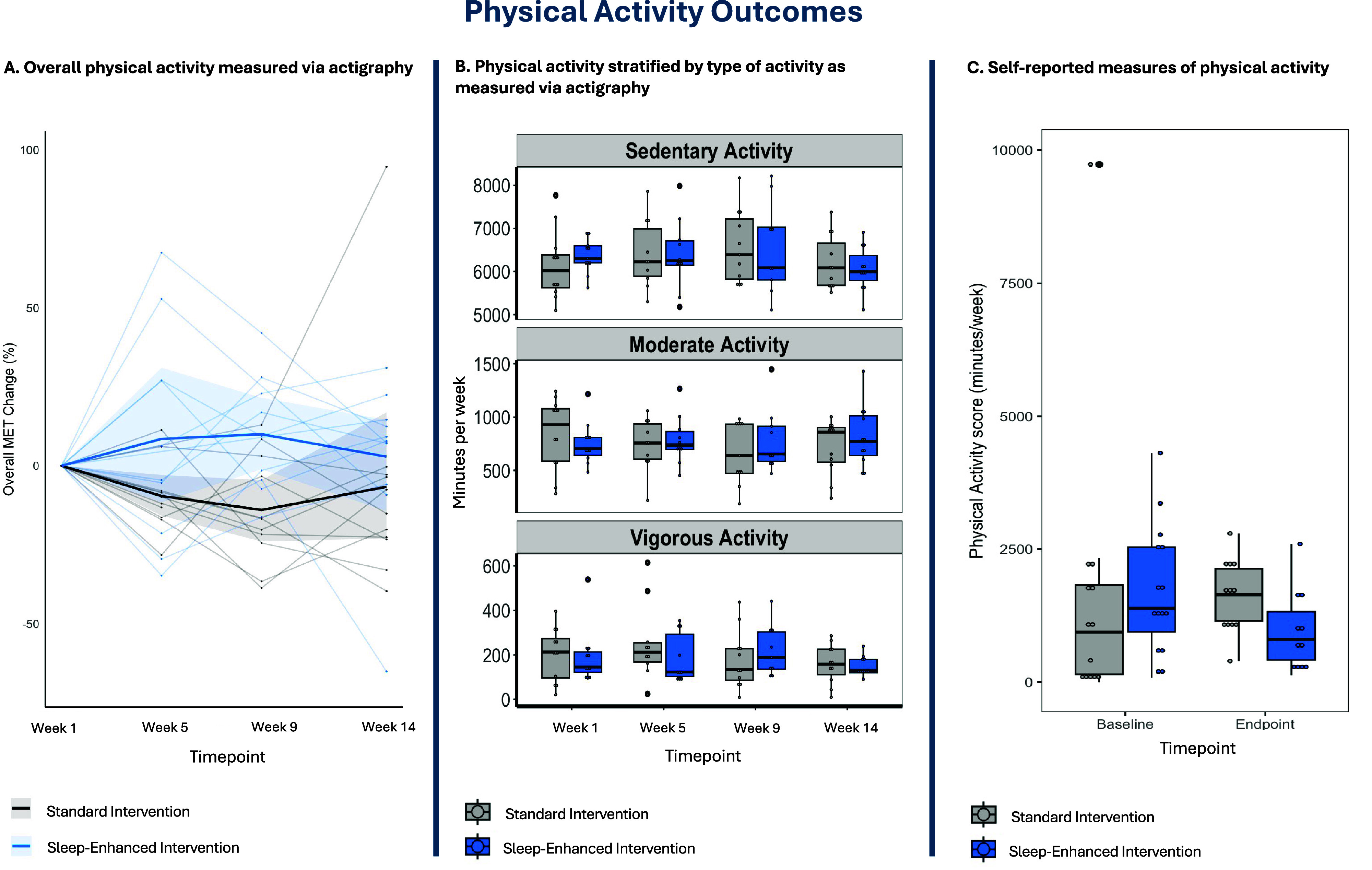
Preliminary findings on the impact of the WHOLE feasibility trial intervention on objective and subjective physical activity outcomes. **(a)** Overall physical activity per week, operationalised as metabolic equivalent of task (MET) minutes, measured using actigraphy across the study period. **(b)** Types of physical activity per week (sedentary, moderate and vigorous), measured using actigraphy and stratified by intervention group across the study period. **(c)** Self-reported overall physical activity at baseline and endpoint measured using the International Physical Activity Questionnaire – Short Form (IPAQ-SF). MET minutes were calculated by multiplying the duration of each activity (minutes) by its corresponding MET value and summing across activities. Values are presented by intervention group across time. Analytical methods are described in the Methods section. Estimates of effect are reported in the Results. Analyses are presented descriptively, as this feasibility trial was not powered to detect intervention effects. Exploratory inferential analyses are provided in the Supplementary Materials for transparency and are not interpreted as confirmatory evidence.

Exploratory analyses of actigraphy and dietary intake data (Tables 2 and 3; Supplementary Tables 3–6) suggested that the SEI may have supported short-term increases in PA. As shown in Figure 4, objective PA levels in the SEI group appeared higher than in the SI group at week 9 (Figure 4a), particularly for vigorous activity (Figure 4b), corresponding to an approximate 20% relative increase in overall weekly MET minutes (≈811-minute mean difference; 95% CI: 271 to 1,893). However, this apparent advantage was not sustained at week 14. By the end of the trial, self-reported PA levels were higher in the SI group, representing an approximate 41% relative difference compared with the SEI group (≈660-minute mean difference; 95% CI: 50 to 1,270, Figure 4c).

**Table 2.** Physical activity, diet, and sleep outcomes by intervention group among participants who actively started the intervention throughout the study duration (*n* = 24)

**Outcome**	**All participants *N* = 24^a^**	**Standard intervention *N* = 12^a^**	**Sleep-enhanced intervention *N* = 12^a^**
**Physical activity^b^**			
**Sedentary behaviour (hours/week)**			
Week 1: Baseline	104.45 (10.12)	102.21 (13.33)	106.45 (6.34)
Week 5	107.14 (13.34)	107.92 (13.45)	107.56 (14.12)
Week 9	109.29 (15.56)	109.65 (14.23)	109.78 (18.22)
Week 14: Endpoint	102.34 (9.45)	103.45 (11.43)	101.23 (9.67)
Mean percentage change in sedentary behaviour at endpoint^c^	−2.42 (6.34)	−0.23 (6.22)	−4.76 (5.13)
**Overall activity (MET minutes/week)^d^**			
Week 1: Baseline	4,765 (1,864)	4,972 (2,223)	4,539 (1,449)
Week 5	4,806 (1,629)	4,982 (1,955)	4,629 (1,308)
Week 9	4,459 (1,922)	4,094 (2,042)	4,905 (1,777)
Week 14: Endpoint	4,301 (1,501)	4,137 (1,650)	4,465 (1,398)
Mean percentage change in overall physical activity at endpoint^c^	−2.04 (31.23)	−7.10 (36.45)	3.04 (25.12)
**Diet and nutrition^e^**			
**Overall energy intake: daily calories (kcal)**			
Week 1: Baseline	1,914 (678)	1,838 (701)	2,006 (674)
Week 5	1,726 (502)	1,734 (595)	1,717 (407)
Week 9	1,614 (535)	1,493 (423)	1,736 (630)
Week 14: Endpoint	1,485 (364)	1,514 (447)	1,445 (230)
Mean percentage change in calorie intake at endpoint^c^	−14.10 (31.12)	−11.25 (28.33)	−19.32 (36.53)
**Diet quality: Healthy Eating Index (HEI)^f^**			
Week 1: Baseline	53.00 (8.41)	53.00 (5.12)	52.00 (10.22)
Week 5	53.80 (7.14)	53.00 (7.92)	54.60 (6.34)
Week 9	51.20 (8.0)	51.20 (10.15)	51.20 (5.85)
Week 14: Endpoint	51.00 (9.61)	53.0 (10.12)	48.00 (8.23)
Mean percentage change in HEI scores at endpoint**^c^**	−6.00 (18.67)	0 (17.12)	−15.06 (17.66)
**Diet quality: Nutrient Adequacy Score (NAS)^g^**			
Week 1: Baseline	0.88 (0.15)	0.86 (0.19)	0.92 (0.09)
Week 5	0.88 (0.11)	0.87 (0.12)	0.89 (0.10)
Week 9	0.83 (0.17)	0.80 (0.16)	0.85 (0.19)
Week 14: Endpoint	0.83 (0.13)	0.81 (0.15)	0.86 (0.09)
Mean percentage change in NAS at endpoint**^c^**	−1.09 (30.41)	1.30 (36.64)	−4.45 (21.22)
**Eating behaviour: eating onset (HH:MM)^h^**			
Week 1: Baseline	09:31 (1:56)	09:11 (2:11)	08:33 (1:06)
Week 5	09:16 (1:21)	09:03 (1:40)	09:47 (2:22)
Week 9	09:35 (1:44)	09:25 (1:42)	09:15 (1:59)
Week 14: Endpoint	09:27 (1:33)	09:37 (2:12)	08:55 (1:02)
Mean percentage change in onset at endpoint**^c^**	1.30 (13.32)	2.23 (16.34)	0 (8.12)
**Eating behaviour: eating offset (HH:MM)^i^**			
Week 1: Baseline	20:51 (1:00)	20:18 (0:46)	20:39 (1:18)
Week 5	21:04 (0:55)	20:09 (1:29)	20:28 (0:42)
Week 9	20:13 (1:39)	20:43 (1:14)	20:00 (2:00)
Week 14: Endpoint	20:36 (1:25)	20:59 (0:47)	20:33 (0:58)
Mean percentage change in offset at endpoint**^c^**	0 (8.11)	2.43 (9.25)	−2.33 (7.45)
**Eating behaviour: eating window (total hours)**			
Week 1: Baseline	10.95 (2.26)	10.24 (2.68)	11.80 (1.30)
Week 5	11.34 (2.04)	11.37 (2.37)	11.31 (1.73)
Week 9	11.10 (1.69)	11.12 (1.77)	10.68 (2.19)
Week 14: Endpoint	11.29 (1.96)	11.08 (1.72)	11.59 (1.32)
Mean percentage change in eating window at endpoint**^c^**	−6.23 (18.23)	0 (17.45)	−15.09 (17.11)
**Sleep**			
**Overall sleep quality^j^**			
Week 1: Baseline	0.01 (0.82)	−0.02 (0.95)	0.02 (0.70)
Week 5	0.01 (0.83)	0.12 (0.74)	−0.12 (0.93)
Week 9	0.01 (0.87)	0.01 (0.97)	0.01 (0.80)
Week 14: Endpoint	0.01 (0.88)	−0.05 (0.83)	0.05 (0.96)
Mean absolute change in sleep quality at endpoint**^k^**	0 (0)	−0.03 (0.87)	0.03 (0.94)
**Sleep duration (hours)**			
Week 1: Baseline	6.50 (0.74)	6.44 (0.84)	6.55 (0.65)
Week 5	6.49 (0.91)	6.52 (0.92)	6.46 (0.96)
Week 9	6.44 (0.87)	6.57 (0.96)	6.27 (0.76)
Week 14: Endpoint	6.48 (0.85)	6.44 (0.84)	6.52 (0.90)
Mean absolute change in duration at endpoint**^k^**	−0.02 (8.23)	0 (7.44)	−0.03 (10.12)
**Sleep latency (minutes)**			
Week 1: Baseline	8.4 (7.81)	10.2 (18.03)	15.0 (18.06)
Week 5	9.0 (7.82)	8.4 (6.07)	15.6 (15.66)
Week 9	7.2 (7.26)	16.8 (19.84)	15.0 (13.23)
Week 14: Endpoint	9.0 (13.27)	18.6 (22.21)	15.6 (18.03)
Mean absolute change in latency at endpoint**^k^**	0.60 (11.81)	8.40 (15.12)	0.60 (15.36)
**Sleep efficiency (%)**			
Week 1: Baseline	85.54 (4.72)	85.63 (5.05)	85.42 (4.67)
Week 5	84.44 (6.15)	85.83 (5.83)	82.91 (6.33)
Week 9	83.15 (6.25)	82.87 (7.25)	83.32 (5.24)
Week 14: Endpoint	82.54 (6.88)	83.38 (6.85)	81.62 (7.07)
Mean percentage change in efficiency at endpoint**^c^**	−3.60 (6.33)	−2.84 (5.21)	−4.53 (7.12)
**Sleep fragmentation (%)**			
Week 1: Baseline	24.34 (7.12)	23.44 (6.63)	24.33 (8.22)
Week 5	24.55 (8.22)	24.65 (9.62)	25.43 (7.43)
Week 9	26.67 (8.34)	28.45 (10.34)	25.33 (7.56)
Week 14: Endpoint	28.12 (9.56)	30.55 (9.67)	26.65 (8.34)
Mean percentage change in fragmentation at endpoint**^c^**	19.23 (27.34)	26.25 (20.12)	11.46 (32.32)
**Social jet lag^l^**			
Week 1: Baseline	0.70 (0.64)	0.63 (0.76)	0.77 (0.50)
Week 5	0.67 (0.94)	0.60 (0.66)	0.75 (1.21)
Week 9	0.57 (1.18)	0.32 (1.35)	0.89 (0.94)
Week 14: Endpoint	0.71 (0.76)	0.65 (0.84)	0.78 (0.71)
Mean absolute change in social jet lag at endpoint**^k^**	0 (0.87)	−0.01 (0.80)	0.01 (0.97)
**Sleep debt^m^**			
Week 1: Baseline	−10.5 (5.24)	−10.8 (5.92)	−10.1 (4.63)
Week 5	−10.4 (5.33)	−9.9 (4.81)	−10.9 (6.12)
Week 9	−10.9 (5.07)	−10.3 (5.53)	−11.6 (4.46)
Week 14: Endpoint	−11.0 (5.87)	−10.9 (5.74)	−11.2 (6.26)
Mean absolute change in sleep debt at endpoint**^k^**	−0.53 (4.09)	0 (3.14)	−1.05 (4.93)

a Values represent mean (SD) of non-missing values from all participants that started the intervention. *N* = 4 enrolled participants dropped out prior to the baseline assessment (week 1).

b Objectively measured using the MotionWatch 8© actigraphy system.

c Expressed as percentage change from baseline (week 1) to endpoint (week 14).

d Activity (MET) minutes were calculated by multiplying the duration of each activity (in minutes) by its corresponding metabolic equivalent of task (MET) value. Total MET minutes represent the sum of all activities over the specified period^(^^74^^)^.

e Dietary intake was assessed using INTAKE24, an online dietary recall tool (https://intake24.co.uk/).

f The Healthy Eating Index (HEI) score was calculated based on dietary intake, assessing adherence to UK dietary guidelines^(^^81^^)^, as previously described^(^^80^^)^. Component scores were summed to generate a total HEI score, with higher values indicating better diet quality.

g The Nutrient Adequacy Score (NAS) was calculated based on nutrient and mineral intake, reflecting the proportion of essential nutrients meeting UK recommended levels^(^^82^^)^. Higher scores indicate greater nutrient adequacy^(^^83^^)^.

h Refers to the recorded time when participants start eating for the day.

i Refers to the recorded time when participants stop eating for the day.

j A composite MW8 sleep quality score was created by averaging the standardised duration, efficiency, and fragmentation scores across the whole week. The fragmentation score was multiplied by −1 prior to averaging^(^^75^^,^^76^^)^. Higher composite scores represent better sleep quality.

k Expressed as absolute change from baseline (week 1) to endpoint (week 14).

l Social jet lag was operationalised as the difference between weekday and weekend sleep midpoints. It reflects the misalignment between the individual’s biological clock and social or work-related schedules, with greater differences indicating higher levels of social jet lag^(^^77^^,^^78^^)^.

m Refers to the cumulative difference between sleep duration and the recommended amount (8h). A positive sleep debt indicates insufficient sleep compared to the recommended amount^(^^79^^)^.

**Table 3.** Sleep and physical activity self-report outcomes by intervention among participants who actively started the intervention at baseline and endpoint (*n* = 24)

**Outcome**	**All participants *N* = 24^a^**	**Standard intervention *N* = 12^a^**	**Sleep-enhanced intervention *N* = 12^a^**
**Sleep disturbance^b^**			
Week 1: Baseline	52.43 (2.81)	51.23 (2.41)	53.84 (2.66)
Week 14: Endpoint	51.82 (3.52)	50.14 (3.43)	53.73 (2.62)
Mean absolute change**^c^**	−0.64 (3.36)	−1.13 (3.61)	−0.10 (3.14)
**Daytime sleepiness^d^**			
Week 1: Baseline	4.83 (2.74)	4.42 (3.23)	5.27 (2.15)
Week 14: Endpoint	4.96 (3.34)	5.08 (3.80)	4.82 (2.93)
Mean absolute change**^c^**	0.13 (2.32)	0.67 (2.42)	−0.45 (2.16)
**Sleep hygiene^e^**			
Week 1: Baseline	18.95 (5.63)	19.83 (5.0)	18.13 (6.13)
Week 14: Endpoint	16.33 (5.22)	17.34 (5.14)	15.36 (5.44)
Mean absolute change**^c^**	−2.64 (4.62)	−2.65 (5.32)	−2.64 (4.15)
**Physical activity^f^**			
Week 1: Baseline	1,666 (1,925)	1,606 (2,581)	1,717 (1,194)
Week 14: Endpoint	1,293 (779)	1,609 (686)	949 (753)
Mean absolute change^c^	−421 (1,921)	−131 (2,510)	−738 (987)

a Values represent mean (SD) of non-missing values from all participants that started the intervention. *N* = 4 enrolled participants dropped out prior to the baseline assessment (week 1).

b Assessed using the Patient Reported Outcomes Measurement Information System (PROMIS) sleep/wake disturbances scale^(^ ^66^ ^)^.

c Expressed as absolute change from baseline (week 1) to endpoint (week 14).

d Assessed using the Epworth Sleepiness Scale (ESS)^(^ ^67^ ^)^.

e Assessed using the Sleep Hygiene Index (SHI)^(^ ^59^ ^)^.

f Assessed using the shortened version of the International Physical Activity Questionnaire (IPAQ-SF)^(^ ^65^ ^)^.

**Figure 5.**
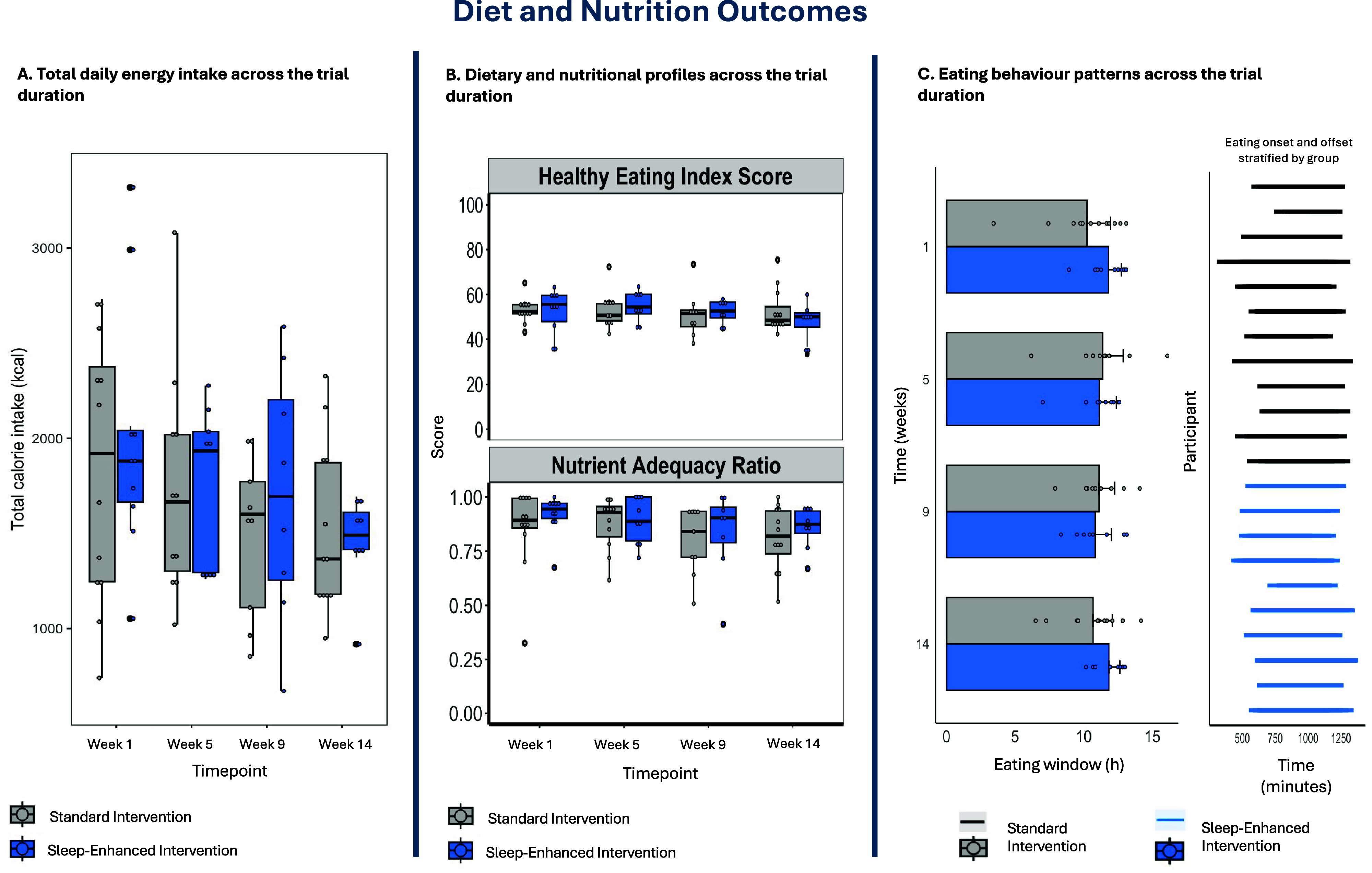
Preliminary findings on the impact of the WHOLE feasibility trial intervention on objective and subjective sleep outcomes. **(a)** Actigraphy-derived measures of sleep, including composite sleep quality, social jet lag, and sleep debt, across the study period. **(b)** Self-reported sleep outcomes, including sleep disturbance, daytime sleepiness, and sleep hygiene. Sleep quality was operationalised as a composite *z*-score based on sleep duration, sleep efficiency, and sleep latency. Social jet lag was operationalised as the difference in sleep midpoint (i.e. the middle of the sleep period) between weekdays and weekends. Sleep debt was operationalised as the cumulative difference between an individual’s recommended sleep duration and actual sleep. Values are presented by intervention group across time. Analytical methods are described in the Methods section. Estimates of effect are reported in the Results. Analyses are presented descriptively, as this feasibility trial was not powered to detect intervention effects. Exploratory inferential analyses are provided in the Supplementary Materials for transparency and are not interpreted as confirmatory evidence.

Exploratory findings also suggested changes in eating behaviour within the SEI group (Figure 5). Both groups reduced energy intake over time; however, the reduction appeared more pronounced in the SEI group, corresponding to an approximate 28% decrease in daily caloric intake by week 14 relative to baseline (≈561-kcal mean difference; 95% CI: 269 to 853, Figure 5a). Additionally, the SEI group exhibited a shorter eating window at week 9, corresponding to a 4% relative decrease compared with the SI group (≈0.44-hour mean difference; 95% CI: −1.68 to −0.22; Figure 5c). However, no clear differences in diet quality were observed between or within groups (Figure 5b).

**Figure 6.**
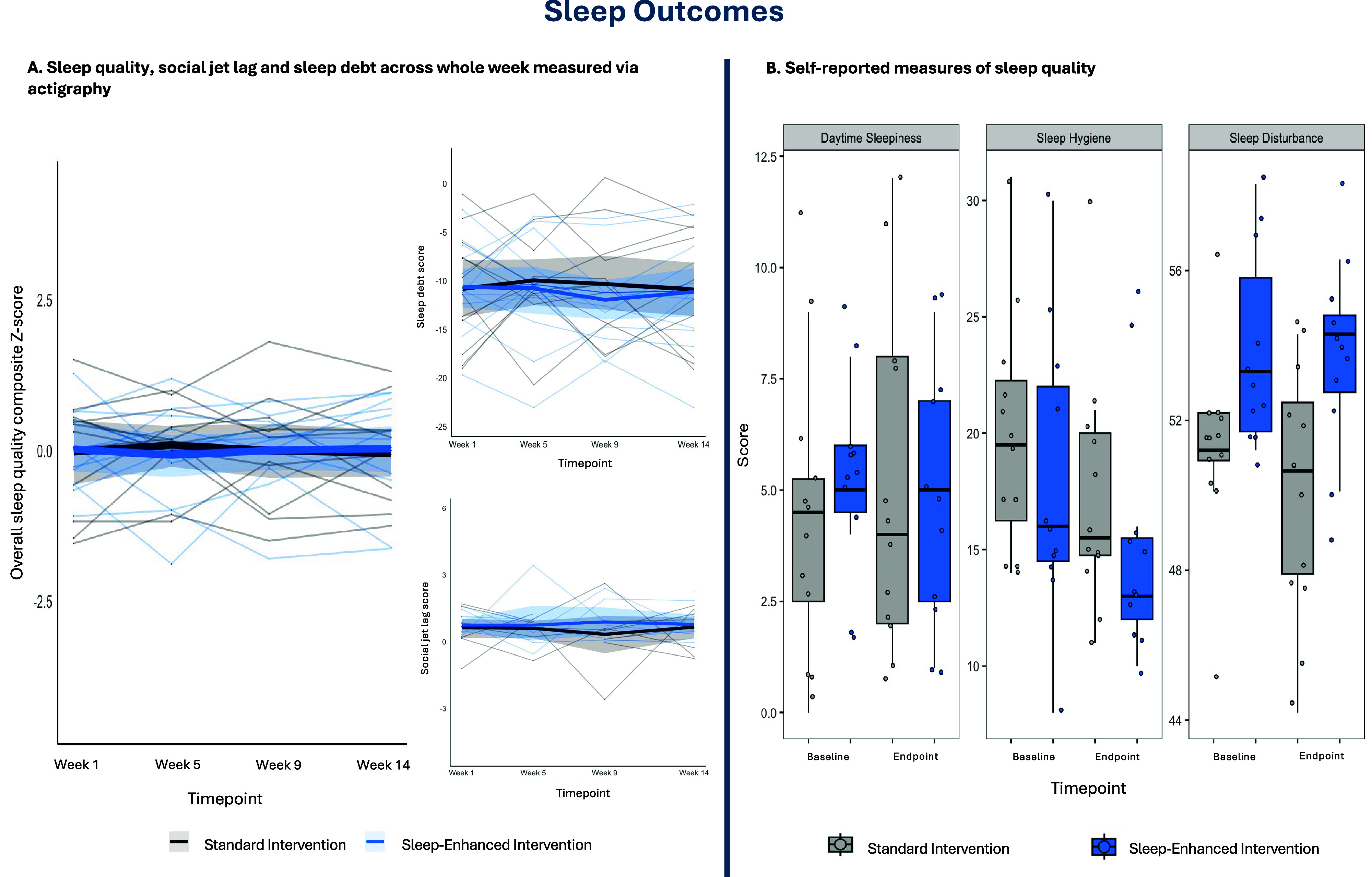
Preliminary findings on the impact of the WHOLE feasibility trial intervention on dietary intake and eating behaviour outcomes. **(a)** Total daily energy intake across the study period, derived from dietary records. **(b)** Diet quality measures, including the Healthy Eating Index (HEI) and Nutrient Adequacy Score (NAS), across the study period. **(c)** Eating behaviour indicators, including eating onset, eating offset and eating window duration, across the study period. Energy intake represents total kilocalories consumed per day. Eating onset and offset refer to the timing of the first and last caloric intake, respectively. Eating window represents the duration between eating onset and offset. Values are presented by intervention group across time. Analytical methods are described in the Methods section. Estimates of effect are reported in the Results. Analyses are presented descriptively, as this feasibility trial was not powered to detect intervention effects. Exploratory inferential analyses are provided in the Supplementary Materials for transparency and are not interpreted as confirmatory evidence.

Objective sleep measures, including sleep quality, social jet lag, and sleep debt, showed no clear differences between or within groups (Figure 6, Supplementary Figure 1). However, both groups reported improvements in sleep hygiene at week 14 relative to baseline (Figure 6b; SI: 13% improvement; ≈2.49-point mean difference; CI: 0.47 to 4.51; SEI: 15% improvement; ≈2.77-point mean difference; CI: 0.44 to 5.10), suggesting perceived behavioural change despite limited objective differences.

Given the exploratory nature of these findings, we next examined whether observed behavioural changes were accompanied by trends in weight-related outcomes – anticipated as primary endpoints in a future full-scale trial – as well as other potential indicators of intervention impact.

##### Anthropometric outcomes

**Figure 7.**
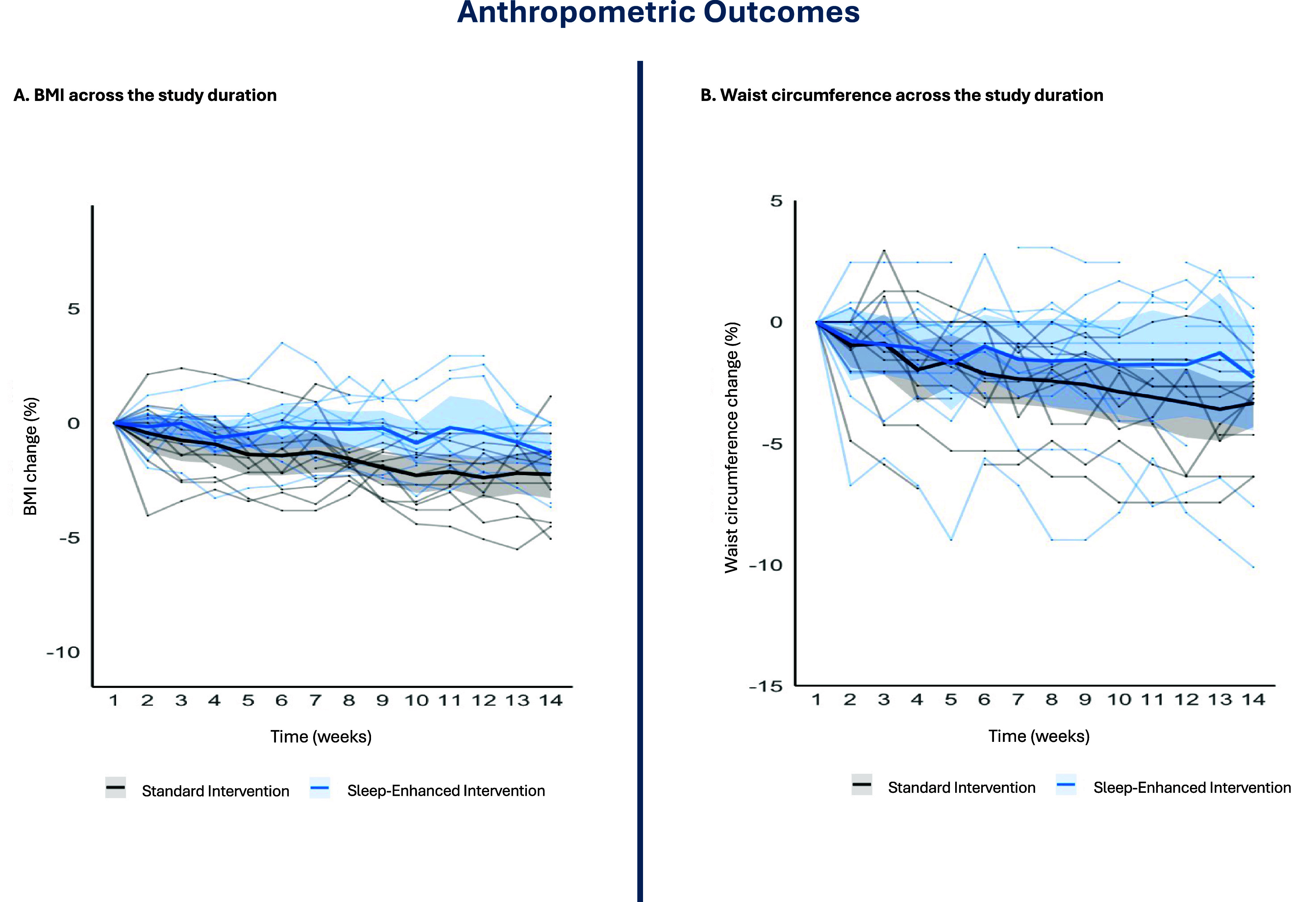
Preliminary findings on the impact of the WHOLE feasibility trial intervention on anthropometric outcomes. **(a)** BMI across the study period. **(b)** Waist circumference across the study period. BMI is expressed in kg/m^2^, and waist circumference in centimetres. Values are presented by intervention group across time. Analytical methods are described in the Methods section. Estimates of effect are reported in the Results. Analyses are presented descriptively, as this feasibility trial was not powered to detect intervention effects. Exploratory inferential analyses are provided in the Supplementary Materials for transparency and are not interpreted as confirmatory evidence.

As shown in Table 4, Supplementary Table 7, and Figure 7, there were no clear differences in overall BMI reduction between groups over time. Both groups showed reductions from baseline in BMI and waist circumference over the intervention period. Mean reductions were numerically greater in the SI group, although CIs were wide and overlapped substantially, indicating considerable uncertainty around these estimates (BMI: SI: 3%; ≈0.90-point mean difference; CI: −3.30 to 1.50; SEI: 2%; ≈0.71-point mean difference; CI: −4.58 to 3.61; Figure 7a; waist circumference: SI: 5%; ≈5-cm mean difference; CI: −11.82 to 1.82; SEI: 1%; ≈1-cm mean difference; CI: −8.08 to 6.08; Figure 7b).

**Table 4.** Anthropometric, well-being and work-related outcomes by intervention among participants who actively started the intervention throughout the study duration (*n* = 24)

**Outcome**	**All participants *N* = 24^a^**	**Standard intervention *N* = 12^a^**	**Sleep-enhanced intervention *N* = 12^a^**
**Anthropometry**			
**BMI **(kg/m²)****			
Week 1: Baseline	30.43 (5.14)	30.43 (3.94)	30.52 (6.33)
Week 2	30.12 (5.26)	30.22 (3.92)	29.92 (6.43)
Week 3	30.04 (5.23)	30.11 (3.82)	29.91 (6.41)
Week 4	29.92 (5.08)	29.41 (3.15)	30.30 (6.41)
Week 5	29.61 (5.12)	29.46 (3.33)	29.85 (6.42)
Week 6	30.62 (5.21)	30.45 (3.63)	30.86 (6.75)
Week 7	30.24 (5.24)	30.04 (4.02)	30.54 (6.43)
Week 8	29.95 (5.21)	29.97 (4.01)	29.90 (6.44)
Week 9	30.07 (5.24)	29.65 (4.11)	30.45 (6.42)
Week 10	30.16 (5.26)	29.53 (4.15)	30.73 (6.33)
Week 11	29.73 (4.92)	29.52 (4.04)	29.92 (6.04)
Week 12	30.12 (5.36)	29.41 (4.33)	30.85 (6.41)
Week 13	29.85 (5.43)	29.57 (4.02)	30.22 (7.22)
Week 14: endpoint	29.74 (5.33)	29.53 (4.11)	29.81 (6.61)
Mean change in BMI at endpoint**^b^**	−1.83 (1.70)	−2.25 (1.88)	−1.37 (1.42)
**Waist Circumference (cm)**			
Week 1: baseline	94 (12)	95 (13)	93 (12)
Week 2	93 (12)	94 (12)	93 (11)
Week 3	93 (11)	94 (12)	93 (11)
Week 4	92 (11)	91 (11)	93 (11)
Week 5	91 (11)	90 (11)	92 (11)
Week 6	94 (12)	92 (13)	95 (12)
Week 7	93 (12)	92 (12)	93 (12)
Week 8	92 (12)	92 (12)	92 (12)
Week 9	92 (11)	90 (11)	93 (12)
Week 10	92 (11)	90 (11)	94 (11)
Week 11	91 (11)	90 (11)	92 (11)
Week 12	92 (11)	90 (11)	93 (12)
Week 13	90 (12)	89 (11)	92 (14)
Week 14: endpoint	91 (11)	90 (11)	92 (13)
Mean change in waist circumference at endpoint^b^	−2.31 (2.44)	−3.35 (1.75)	−1.16 (2.65)
**Mental health and well-being**			
**Depressive symptomatology^c^**			
Week 1: baseline	6.13 (5.01)	7.74 (5.81)	4.43 (3.43)
Week 14: endpoint	5.74 (5.50)	8.02 (6.46)	3.13 (2.75)
Mean change in depressive symptomatology**^d^**	−0.4 (3.91)	0.3 (4.72)	−1.3 (2.82)
**Anxiety symptomatology^e^**			
Week 1: baseline	5.63 (5.51)	6.63 (7.05)	4.50 (3.07)
Week 14: endpoint	4.32 (5.12)	6.81 (5.93)	1.51 (1.42)
Mean change in anxiety symptomatology^d^	−1.34 (3.25)	0.33 (3.06)	−2.91 (2.72)
**Workplace-related outcomes**			
**Work engagement^f^**			
Week 1: baseline	3.43 (0.84)	3.32 (0.74)	3.54 (0.94)
Week 14: endpoint	3.63 (1.03)	3.68 (0.99)	3.58 (1.12)
Mean change in work engagement**^b^**	7.21 (27.32)	11.34 (26.12)	3.72 (28.07)
**Job satisfaction^g^**			
Week 1: baseline	14.32 (2.85)	14.28 (2.37)	14.46 (3.42)
Week 14: endpoint	13.73 (3.42)	14.42 (3.35)	13.06 (3.62)
Mean change in job satisfaction**^b^**	−4.06 (15.22)	1.09 (15.12)	−10.78 (13.54)
**Social support and coping^h^**			
Week 1: baseline	11.23 (3.82)	13.13 (3.66)	9.14 (2.82)
Week 14: endpoint	11.26 (4.14)	13.45 (3.34)	8.82 (3.61)
Mean change in support and coping**^b^**	6.21 (40.09)	9.07 (33.11)	3.15 (48.51)
**Burnout^i^**			
Week 1: baseline	2.37 (0.34)	2.41 (0.35)	2.33 (0.34)
Week 14: endpoint	2.29 (0.40)	2.30 (0.40)	2.27 (0.43)
Mean change in burnout**^b^**	−3.21 (15.23)	−3.05 (19.12)	−3.12 (10.05)

a Values represent mean (SD) of non-missing values from all participants that started the intervention. *N* = 4 enrolled participants dropped out prior to the baseline assessment (week 1).

b Expressed as percentage change from baseline (week 1) to endpoint (week 14).

c Assessed using the Patient Health Questionnaire-9 (PHQ-9)^(^ ^69^ ^)^.

d Expressed as absolute change from baseline (week 1) to endpoint (week 14).

e Assessed using the Generalised Anxiety Disorder Questionnaire (GAD-7)^(^ ^68^ ^)^.

f Assessed using the Short Utrecht Work Engagement Scale (UWES-9)^(^ ^70^ ^)^.

g Assessed using the Short Index of Job Satisfaction (SIJS)^(^ ^71^ ^)^.

h Assessed using State Self-Control Capacity Scale (SSCCS)^(^ ^72^ ^)^.

i Assessed using the Oldenberg Burnout Inventory (OLBI)^(^ ^73^ ^)^.

*Note*: Waist circumference was reported to the nearest cm. Mental well-being and workplace well-being were only assessed at two timepoints, that is, baseline and endpoint.

These findings suggest that both interventions may be associated with modest short-term improvements in anthropometric measures, but no clear advantage of the SEI was evident.

Finally, we explored whether the intervention supported broader positive changes that might enhance its overall acceptability, focusing on mental well-being and workplace-related experiences.

##### Mental well-being and workplace-related experiences

**Figure 8.**
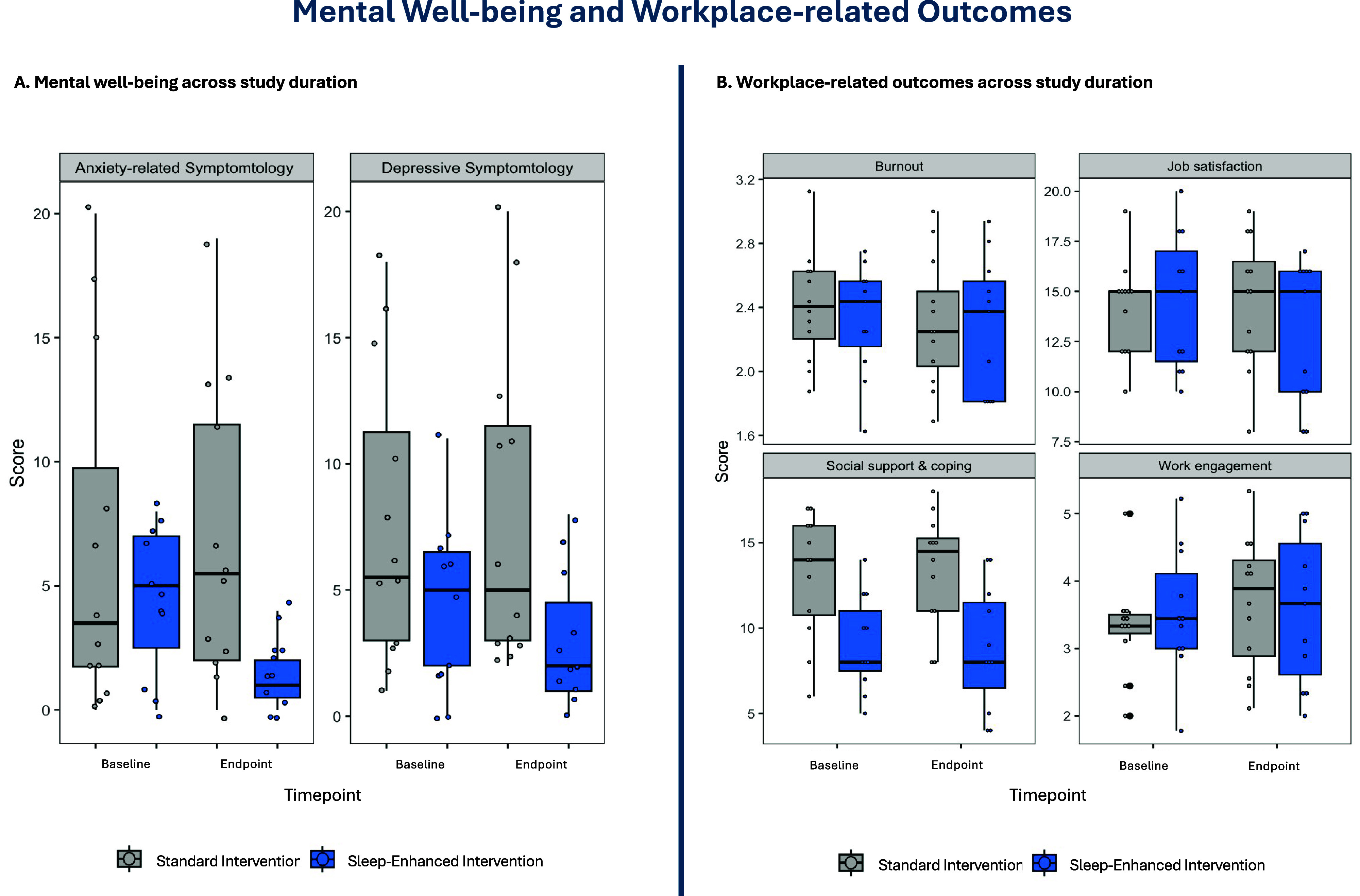
Preliminary findings on the impact of the WHOLE feasibility trial intervention on mental well-being and workplace-related outcomes. **(a)** Anxiety and depressive symptomatology across the study period. **(b)** Workplace-related outcomes, including work engagement, job satisfaction, social support and coping, and burnout. Mental well-being outcomes were assessed using validated self-report questionnaires, and workplace outcomes using established scales as described in the Methods section. Values are presented by intervention group across time. Analytical methods are described in the Methods section. Estimates of effect are reported in the Results. Analyses are presented descriptively, as this feasibility trial was not powered to detect intervention effects. Exploratory inferential analyses are provided in the Supplementary Materials for transparency and are not interpreted as confirmatory evidence.

While no clear differences in depressive symptoms or workplace-related measures between or within groups were observed, participants in the SEI group showed a 67% relative reduction in anxiety symptoms by week 14 (≈3.0-point mean difference; 95% CI: 1.64 to 4.34), and a 78% larger reduction than observed in the SI group (≈5.3-point mean difference; 95% CI: 2.86 to 7.74; Table 4; Supplementary Table 8; Figure 8).

Overall, these exploratory findings suggest that the SEI may confer potential benefits for mental well-being, although conclusions regarding effectiveness cannot be drawn from this feasibility study.

### Qualitative data

A qualitative process evaluation was used to explore participants’ experiences and satisfaction in greater depth. Analysis identified four overarching themes: *motivational drives for participation*, *experiences of adherence*, *perceived benefits and consequences*, and *areas for improvement*.

#### Theme 1: motivational drives for participation

Participants described seeking a holistic and structured approach that supported change across multiple lifestyle behaviours. The intervention was seen to ‘*kick-start a process of trying to lose some weight, trying to have healthier sleep patterns, and just a healthier lifestyle all around’* (Participant (P)7, SEI). The opportunity to focus on sleep, food, and exercise over a defined period, with expert input, was perceived as beneficial: ‘*I felt that I would benefit from advice from a nutritionist… and from the very fact that I’d be focusing more on sleep, exercise, and food*’ (P4, SI). Others valued contributing to research, viewing it as ‘*the responsible thing to do’* P10, SEI).

#### Theme 2: experiences of adherence

Accountability and support were important to engagement. Personalised, non-judgemental advice and regular check-ins ‘*tailored to the individual… that was really good for accountability*’ (P4, SI) helped maintain motivation. Peer support fostered a sense of shared experience – ‘*sharing your experiences makes you realise that you’re not the only person who struggles with sleep, eating, and exercise*’ (P1, SEI) – and enhanced intervention coherence.

However, some participants found adhering to all components challenging, particularly in the SEI group, where making multiple changes simultaneously disrupted routines: ‘*It was difficult to do everything and keep everything on track… it’s a lot of changes to my usual lifestyle*’ (P1, SEI). Moreover, practical barriers, including illness, busy schedules, and technical issues with tools such as the dietary recording app, added burden: *‘The [intake24] app needs some serious updates… sometimes the search function wasn’t always the best.’* (P4, SI).

#### Theme 3: perceived benefits and consequences

Participants reported a range of perceived benefits, particularly improved sleep, energy, and greater alignment across behaviours, which aligned with the pilot quantitative data (Figures 4–6). ‘*Having better sleep means that you have more energy… and when I exercise more, I eat less*’ (P9, SEI). Self-monitoring also raised awareness: ‘*I’m certainly eating far better. I’m snacking far less… I’m looking at portion sizes. I’m looking at my sleep much more*’ (P2, SI). Psychological gains were also noted – ‘*That was quite nice to have that positive encouragement… rather than… where if I couldn’t do all 10 things, then I failed*’ (P6, SEI).

However, not all components were universally motivating. Weekly weigh-ins prompted anxiety for some: ‘*I always did slightly dread the Monday because like, I’ve eaten so much this weekend*’ (P7, SEI). This may have contributed to lower engagement in some behaviours and missing data towards the end of the trial.

#### Theme 4: areas for improvement

Participants recommended a phased introduction to behaviour changes to reduce cognitive and practical burden: ‘*I would have introduced the changes sequentially or gradually… that would have been a bit more helpful*’ (P1, SEI). Technical improvements were also recommended, including clearer feedback from wearables: ‘*It always felt like it wasn’t doing anything… I wasn’t sure whether it was active or not*’ (P7, SEI). Additionally, participants requested clearer structure in the group sessions – ‘*Maybe more clear structures… like this week, we’re going to discuss nutritional value*’ (P2, SI) – and greater peer connection: ‘*I would have liked them more frequently… even if there was just like a Teams channel*’ (P4, SI). Importantly, while many valued the group component, some felt that being in very small groups limited opportunities for shared experiences: they struggled to relate to others or felt they had little in common. This not only reduced the potential for peer support but also made session delivery more difficult, as noted by the facilitator (DM). Several participants also expressed a preference for in-person interaction: ‘*It’s just nice to talk to people in person… interact with people is always helpful*’ (P11, SEI).

## Discussion

### Trial and intervention feasibility and process evaluation

This feasibility trial evaluates a novel SEI for weight management, targeting PA, diet, and sleep, versus a SI focusing only on PA and diet. The trial indicates good operational feasibility, with successful recruitment and procedures consistent with CONSORT and TIDieR guidance,^(37,38)^ high retention (82% overall; 96% post-randomisation), and good compliance (84%) – comparable with similar behavioural interventions, which often report lower compliance (60–70%) than pharmacological trials.^(46,56)^

However, eligibility restrictions and the SEI’s complexity may limit enrolment (22%). A future trial could expand recruitment through broader outreach, such as community partnerships (e.g. WFH hubs, cafes, coffee shops), targeted local advertising, and referral networks.^(90–92)^ These strategies may reduce opportunity costs by making participation more accessible and less disruptive to participants’ routines.

Retention is another key strength. Although early attrition is higher in the SEI group, this did not appear to be clearly attributable to the intervention itself. Once engaged, SEI participants demonstrate strong adherence, reinforcing intervention coherence (i.e. understanding and sense-making of the intervention) and overall acceptability of trial procedures.

While the overall incidence of missing data is low, particularly for the SEI group (2%), missing data fluctuates weekly, sometimes exceeding 10% (e.g. weeks 5–9) for measures like actigraphy and dietary intake. However, technical and logistical challenges account for most missing data, such as issues with the wearable device, participant absences, and missed food diaries or weight check-ins, rather than a lack of engagement. These challenges, while common in remote, self-reported studies and research involving wearable technologies,^(93–95)^ highlight the need for improved digital tools and additional support. A future trial may benefit from regular, structured check-ins, personalised digital support, simplified reporting systems and incentive-based rewards to reduce participant burden and improve data quality.^(96)^

The mixed-methods process evaluation indicates that both the intervention and trial design are generally acceptable across multiple TFA domains. Participants describe the intervention as relevant and beneficial, particularly appreciating perceived effectiveness, self-efficacy, and ethicality, highlighting personal relevance, health gains, and alignment with values. Personalised guidance, peer support, and accountability appear to contribute to motivation and sustained engagement, consistent with prior evidence on the importance of self-monitoring and goal-setting in behaviour change,^(97–105)^ which form the foundation of the intervention.

Notably, exploratory findings indicate potential improvements in eating behaviour, objective PA, sleep hygiene, and anxiety within the SEI group, with no adverse events. While these findings remain preliminary, as the trial is not designed to assess definitive effectiveness, they warrant further investigation in a future, fully powered trial. However, it is noteworthy that only subjective improvements in sleep hygiene are observed, reflecting a common discrepancy in behavioural interventions,^(106,107)^ which may suggest limits in intervention coherence, particularly in how participants understand and apply the sleep component or may highlight the inherent complexity of sleep behaviours. Therefore, more intensive or tailored approaches, such as cognitive–behavioural therapy for insomnia^(108)^ and/or digital sleep tools,^(109)^ may be necessary in future iterations.

Interestingly, SI participants, despite not receiving the sleep component, also report improvements in sleep hygiene. While this may reflect a Hawthorne effect,^(110)^ it may also reflect indirect benefits of diet and PA counselling. Research has shown that regular PA can enhance sleep duration and quality by promoting deeper sleep cycles,^(111)^ while a plant-rich diet (fruits, vegetables, whole grains, legumes, olive oil, and seafood) with limited processed foods and sugar-sweetened beverages can improve sleep quality.^(10)^ To better isolate intervention effects, a future trial should include a no-intervention control group and increase sample size to strengthen efficacy evaluations beyond feasibility.

While the SEI demonstrates preliminary indications of multi-behavioural change, qualitative data suggest that its multiple simultaneous demands may have placed a significant burden on some participants, limiting their capacity for full engagement, particularly in the absence of phased support. These challenges may help explain the plateau in behavioural gains we observe after week 9 in the SEI group. The combined cognitive and emotional demands of the SEI may have reduced participants’ ability to maintain engagement and focus on complex behaviours like diet quality and sleep, which often require more targeted attention due to their inherent complexity.^(112,113)^

Additionally, although both groups show improvements in BMI and waist circumference, greater reductions in the SI group may reflect differences in intervention complexity. Given the complex demands of the SEI, the less pronounced weight loss observed may reflect increased stress, compensatory eating, or reduced focus.^(114–117)^ Therefore, simplifying or sequencing behavioural targets may help reduce this burden and enhance long-term engagement.

### Strengths, limitations, and future implementation

To our knowledge, this is the first UK intervention targeting three modifiable factors, including the often-overlooked role of sleep, to promote weight management. Our robust mixed-methods design, diverse sample, and positive participant feedback supports the feasibility of both the intervention and trial procedures. Importantly, given the rise of remote work models, our findings offer timely preliminary support for adapting multi-component weight management interventions to these settings, underscoring the need for a larger trial to evaluate effectiveness and implementation.

However, a key limitation of this study is the small sample size, which restricts the precision of feasibility estimates and limits the generalisability of findings. While small samples are typical of feasibility studies, the modest number of participants means that estimates of recruitment, retention, adherence, and behavioural trends should be interpreted cautiously. The study was not designed or powered to detect intervention effects, and observed changes should therefore be considered preliminary and hypothesis-generating, requiring confirmation in a larger, fully powered trial.

Moreover, this feasibility trial highlights that while multi-component interventions can engage and benefit participants, balancing intensity and manageability will be critical. Future iterations should consider a phased approach, introducing behavioural modifications sequentially rather than simultaneously, and extending duration to improve adherence and support more sustained behavioural change.

Additionally, acceptability data were collected only from participants, and facilitator perspectives were not formally assessed. Input from intervention providers could offer valuable insights into delivery burden, fidelity, feasibility, and practical implementation challenges. Future trials should therefore incorporate facilitator feedback to support optimisation, scalability, and real-world implementation of the intervention.

To optimise engagement, compliance, and data integrity, refining intervention tools, such as improving the usability of food diaries and sleep/activity-tracking systems, will be essential. Strengthening group session structures and increasing peer support opportunities could further enhance motivation and accountability. Offering flexible delivery options, including in-person components, may also improve accessibility and adherence.

It is important to note that this intervention was supported by the host organisation’s Organisational Development team, with sessions held during working hours – a key factor to consider when interpreting feasibility and acceptability. Recruitment from a single workplace may also have introduced the possibility of cross-group contamination through informal interaction among colleagues, despite efforts to minimise interaction between intervention arms. Moreover, as this was a feasibility trial, no cost analysis was conducted. Future implementations should include a cost–benefit analysis over a longer period, where organisational outcomes (e.g. absenteeism, presenteeism) can be assessed. Finally, a future trial should consider workplace stigma around body weight and size^(118)^ and avoid reinforcing sole individual responsibility^(119)^ by engaging broader stakeholders, including the Institute for Employment Studies.^(120)^

### Conclusion

This feasibility trial provides valuable insights for optimising intervention design in a full-scale randomised controlled trial. Exploratory findings suggest that the SEI may support multi-behavioural change. Given that weight loss alone is insufficient for sustained health improvements,^(121)^ the SEI’s holistic approach aligns well with the UK Better Health campaign,^(122)^ warranting evaluation in a fully powered trial. However, rapidly introducing multiple behavioural components may hinder long-term adherence and feasibility of a future full-scale trial. Striking a balance between comprehensiveness and manageability will be crucial to maximise potential effectiveness and sustained engagement in future trials.

## Supporting information

10.1017/jns.2026.10104.sm001 Du Preez et al. supplementary material 1 Du Preez et al. supplementary material

10.1017/jns.2026.10104.sm002 Du Preez et al. supplementary material 2 Du Preez et al. supplementary material

10.1017/jns.2026.10104.sm003 Du Preez et al. supplementary material 3 Du Preez et al. supplementary material

## Supplementary material

The supplementary material for this article can be found at https://doi.org/10.1017/jns.2026.10104.
