# Supplementary material for: Randomised feasibility trial of a remotely delivered holistic UK employee programme combining tailored sleep hygiene, diet, and physical activity counselling for weight management: a mixed-methods evaluation
Source: J Nutr Sci. 2026 Jun 18;15:e45. doi: 10.1017/jns.2026.10104 (PMC13279985; doi:10.1017/jns.2026.10104)
Supplement: Du Preez et al. supplementary material 1 — Du Preez et al. supplementary material [file S2048679026101049sup001.docx]

**SUPPLEMENTARY MATERIALS**

**Randomised feasibility trial of a remotely delivered holistic UK employee programme combining tailored sleep hygiene, diet, and physical activity counselling for weight management: a mixed-methods evaluation**

Andrea Du Preez^1*,^ Danae Marshall^1^*, Lorraine Kelly^2^, Kirti Swift^2^, Zak Evans^3^, Michael Clinton^3^, Rakhee Doshi^4^, Charlotte Fitzhugh^5^, Benjamin Gardner^4^, Rachel Gibson^1^* and Wendy Hall^1^*

(1). Department of Nutritional Sciences, School of Life Course & Population Sciences, Faculty of Life Sciences & Medicine, King’s College London, 150 Stamford Street, London SE1 9NH, UK; (2). Organisational Development, King's College London, 5-11 Lavington Street, SE1 0NZ, UK; (3). Department of Human Resource Management & Employment Relations, King’s Business School, Bush House, 30 Aldwych, London WC2B 4BG, UK; (4). Research Institute for Sport and Exercise Sciences, Liverpool John Moores University, Liverpool, UK; (5). School of Psychological Sciences, University of Surrey, Guildford, GU2 7XH, UK.

***Authors contributed equally to the study and manuscript.**

**Keywords:** Feasibility trial, holistic, lifestyle intervention, weight management, sleep, diet, physical activity.

**Correspondence to:** Dr Rachel Gibson, email: [rachel.gibson@kcl.ac.uk](mailto:rachel.gibson@kcl.ac.uk); or Dr. Andrea Du Preez, email: [andrea.du_preez@kcl.ac.uk](mailto:andrea.du_preez@kcl.ac.uk). Franklin Wilkins Building, 150 Stamford Street, London SE1 9NH, UK.

**CONTENT**

**Methods and materials**

**Results, tables and figures**

**Tidier & CONSORT checklists**

**References**

**METHODS AND MATERIALS**

**Trial intervention and measures**

***Intervention***

*Goal setting during one-to-one consultations:* At the initial consultation, the number of goals—covering physical activity (PA) and diet (both groups) and sleep (SEI only)—was deliberately limited based on what the participant felt comfortable managing. Additional goals were introduced gradually as participants demonstrated progress with earlier targets. In follow-up consultations, existing goals, action plans, and behavioural cues were reviewed and adapted as needed. Participants were encouraged to reflect on their progress since the last session, discussing what had gone well and identifying any ongoing challenges. Data from relevant self-monitoring tools (e.g., dietary intake, physical activity logs) were used to guide the discussion, alongside the participant’s own behaviour records. Barriers to progress were explored, and tailored strategies—including the development or revision of “if…then…” plans—were introduced to support behaviour change. Where goals were unmet, overly difficult, or causing distress, they were adjusted to better suit the participant’s needs. If motivation appeared to be a limiting factor, motivational strategies from the initial consultation were revisited. For participants who were progressing well and wished to advance further, goals were made more challenging or additional goals were introduced (e.g., increasing the number of days meeting PA targets, or adding new dietary changes such as including more fibre-rich foods).

*Script Elicitation:* Script elicitation was conducted to examine participants' cognitive representations of routine behaviours related to sleep. Participants were prompted to recall and describe typical sequences of actions associated with these behaviours using a combination of **free recall** (unstructured descriptions of routine activities) and **structured prompts** (targeted questions guiding participants through key aspects of the behaviour). This approach allowed for an in-depth understanding of how participants conceptualise and engage with these behaviours in their daily lives to map current and planned bedtime routines, identify barriers, and develop solutions ^1^.

**Measures**

*Baseline only*

***The Automated Morningness-Eveningness Questionnaire (AutoMEQ)*** was used to assess chronotype ^2^. This questionnaire determines whether a participant is more of a "morning person" or an "evening person." Resulting scores range from 16–86 ("definite evening type" to "definite morning type"). The questionnaire website also generates an estimated "melatonin onset time" and "natural bedtime" (https://chronotype-self-test.info/index.php?sid=61524&newtest=Y).

***The Alcohol Use Disorders Identification Test (AUDIT)*** was used to assess alcohol consumption and its consequences ^3^. The questionnaire consists of ten items evaluating alcohol use and drinking-related behaviours. Participants responded on a 0–4 scale, and total AUDIT scores were calculated as the sum of all responses. Scores are categorized into risk levels, with higher scores indicating greater alcohol-related risks. Since the AUDIT assesses alcohol use over the past 12 months, it was only administered at baseline.

*Baseline and follow-up*

***The short-form International Physical Activity Questionnaire (IPAQ-SF)*** was used to subjectively measure physical activity ^4^. This seven-item scale assesses time spent walking and engaging in vigorous, moderate, and sedentary activities over the past seven days. Metabolic Equivalents (METs) minutes were calculated using the standard formula:

- Vigorous activity: minutes × 8 METs
- Moderate activity: minutes × 4 METs
- Walking: minutes × 3.3 METs

A total physical activity score was computed by summing all MET scores. Sedentary behaviour was measured by asking participants how many hours they spent sitting over the past seven days.

**Sleep** was assessed using three short questionnaires: the Patient-Reported Outcomes Measurement Information System (PROMIS) Sleep/Wake Disturbances Scale, the Epworth Sleepiness Scale (ESS), and the Sleep Hygiene Index (SHI).

***The PROMIS*** was used to measure sleep disturbance ^5^. This questionnaire consists of eight items, asking participants to rate their sleep experiences over the past seven days. Sample statements include “My sleep was restless” and “I was satisfied with my sleep.” Each item is rated on a five-point scale from 0–5. Scores were summed and converted to T-scores using the official PROMIS conversion table, where higher scores indicate greater sleep and wake disturbances.

***The ESS*** was used to assess daytime sleepiness **^6^.** This questionnaire consists of eight items that evaluate an individual's tendency to fall asleep in various daily situations. Participants rated their likelihood of dozing off while engaging in different activities. Each item is scored on a four-point scale from 0–3. Total ESS scores range from 0–24, with higher scores indicating greater daytime sleepiness.

***The SHI*** was used to measure sleep hygiene **^7^.** This 13-item self-report measure evaluates sleep-related behaviours. Sample items include “I take daytime naps lasting two or more hours” and “I stay in bed longer than I should two or three times a week.” Each item is rated on a five-point scale from 0–4. Total SHI scores range from 0–52, where higher scores indicate worse sleep hygiene.

**Mental well-being** was assessed using two questionnaires: the Generalized Anxiety Disorder Questionnaire (GAD-7) and the Patient Health Questionnaire (PHQ-9).

***The GAD-7*** measured anxiety levels over the past two weeks ^8^. This seven-item questionnaire screens for generalized anxiety disorder (GAD). Participants rated how often they experienced anxiety symptoms on a four-point scale, from 0–3. Total scores (ranging from 0–21) fall into predefined categories of anxiety severity. Participants with a score of 10 or above were contacted and encouraged to consult a GP regarding potential GAD.

***The PHQ-9*** was used to assess depression and depressive symptoms ^9^. Participants responded to nine questions about the frequency of depressive symptoms over the past two weeks, using the same four-point scale (0–3). Total scores (ranging from 0–27) are categorized into five levels of depression severity. Before completing the questionnaire, participants were informed that their responses were not being monitored in real time and were provided with mental health resources. Participants with a score of 10 or above were contacted and advised to consult a GP.

**Workplace-related experience** was assessed using four short questionnaires: the Short Utrecht Work Engagement Scale (UWES-9), the Short Index of Job Satisfaction (SIJS), the State Self-Control Capacity Scale (SSCCS), and the Oldenburg Burnout Inventory (OLBI).

***The UWES-9*** measures how often participants experience nine specific feelings about their job ^10^. Responses are given on a 0–6 scale, where 0 = never and 6 = every day. The scale consists of three subscales: vigour (three items), dedication (three items), and absorption (three items). Subscale scores were calculated as the average of their respective items, and a total UWES-9 score was also computed, with higher scores indicating greater work engagement. Due to a formatting error in the initial UWES-9 questionnaire (a missing response column), 10 participants had to retake the questionnaire after baseline. Of these, 9/10 resubmitted their results. As a result, findings from this scale should be interpreted with caution.

***The SIJS*** consists of four items adapted from the Index of Job Satisfaction ^11^. It assesses overall job satisfaction through statements such as “I am not happy at my job” and “Most days I am enthusiastic about my job.” Participants rated their agreement on a five-point scale ("strongly disagree" to "strongly agree"), with two negatively worded items being reverse-scored. The total SIJS score ranges from 4–20, where higher scores indicate greater job satisfaction.

***The SSCC*S** was adapted to include four items assessing self-control over the past two weeks ^12^. Participants rated their agreement with statements such as “It has taken a lot of effort to concentrate on things.” Responses were scored from 1–5. A total SSCC score was calculated as the average of all responses, with lower scores indicating stronger self-control capacity.

***The OLBI*** assesses burnout symptoms in the workplace ^13^. Participants responded to 16 statements on a four-point scale ("strongly disagree" to "strongly agree"). A total OLBI score was computed as the average of all responses, where higher scores indicate greater burnout severity. Additionally, two subscale scores were calculated: disengagement (eight items) and exhaustion (eight items).

*Post-completion: Qualitative interview scripts*

***Participant Interview Schedule: Sleep-Enhanced Intervention***

Thank you for agreeing to speak to me today. In this interview I would like to talk to you about your experiences of the programme and how you are getting on now. The interview should take about half to three quarters of an hour, but if you want to take a break or stop at any me just tell me and we can stop immediately. You don’t have to answer any question you don’t want to, so if that is the case, please just say so and we can move on to something else. I’d just like to reassure you, again, of confidentiality. Your name will not be attached to the transcripts of this interview or included in any reports of our findings from these interviews. Please feel free to add anything that you think is important but which I may not ask you about. Do you have any questions? I’d just like to check if you are happy for me to record the interview? Shall we begin?

Topics to cover

**Section 1-Motivation for the participant**

1. Can you tell me why you signed up to this study?
2. As you may know, one of the aims of this initiative is to support people to lose weight. Have you tried any weight loss methods before (and how successful were you)?
3. What made you want to lose weight on that past occasion?
4. What made you decide to participate in the programme, and did the fact it was part of a research study affect your decision? - explore why they entered the study, expectations of research study?

**Section 2-Expectations of participation**

1. What were your expectations of the programme?
2. What were you expecting from the initiative

- Was there anything else you would like to have known?
- Was there anything you did not understand?
- Were your expectations met/not met?

1. How easy or hard was it to follow and understand the guidance?

- Is there anything you feel that could be done to make the guidance easier?
- Was there anything that was unclear and why?

**Section 3-Perceived advantages and disadvantages of participation**

1. How did you get on with trying to stick, day to day, to the advice that you were given on the programme?
2. Did you experience any difficulties when following the programme?
3. If so, how could these have been made more easier or practical for you?
4. Were there any tips or tasks you found particularly easy on the program?
5. Why were they easy for you?
6. Aspects of the programme that you found most helpful? Explore what these are and why
7. Any aspects you found less helpful? Explore areas for improvement, anything that could have been done differently?
8. Is there anything from the programme that you are still doing?
9. Imagine one of your friends told you they were considering whether to sign up to this programme, and they wanted your views on it. What would you tell them?
10. Imagine you were running this programme and had to improve it. What one or two improvements would you make, and why?’

**Section 4-Experiences of participation i.e., were their expectations met?**

You’ve told us that you expected X, Y, Z. Now let’s talk about your actual experiences.

1. How did you get on with the programme?’

Thinking about the study programme…

1. Which parts of the programme they found most helpful?

Thinking about the one-to-one sessions with the nutritionist…

1. How did you get on with the one-to-one sessions with the nutritionist?
2. Did you attend if so, how many did you attend?
3. If no, ask why they didn’t attend and explore any difficulties with engaging in them
4. What was/was not useful about the session)
5. Were there too many/not enough sessions, frequency?

Thinking now, about the group sessions…

1. How did you get on with the group sessions?
2. Did you attend the group sessions and if so, how many did you attend?
3. How useful did you find them?
4. If you didn't attend - why not (Explore the barriers to attending-anything that made this process difficult)
5. If yes, how did you get on with them? (What was and what was not useful)
6. Were there too many/not enough sessions, frequency? How did you feel about the group sessions ending?

Explore the components and materials used in the programme…

- 1. MotionWatch - Did you use it? If so, how did you get on with using it/what were your experiences? how often did you use? If no, explore why? Were there any barriers or reluctance to wearing the watch?
  2. Kings’ sports app - Did you use it? If so, how did you get on with using it/what were your experiences? how often did you use? If no, explore why? Were there any barriers or reluctance to using the app?
  3. Dietary Record
- What did you think about keeping a dietary record?
- How often did you engage in this?
- How did you find it?
- Did you face any problems when doing this?
  1. Sleep Diary
- What did you think about keeping a sleep diary?
- How often did you engage in this?
- How did you find it?
- Were there any problems you faced when using it?
  1. Weekly Weighing
- We encouraged you to weigh yourself weekly as opposed to other times; did you find this useful/helpful?
- How often did you weigh? Ask if they did keep weighing themselves to the instructed amount?
- If not why, if yes, how did they find it?
- When recording your weight how did this make you feel?
  1. Sleep Advice
- How did you find the involvement of sleep in the programme and the advice given?
- How easy was it to make changes to your bedtime routine?
- Did your sleep schedule improve? When comparing your sleeping pattern prior to the start of the programme have you noticed any changes? If so, what were those changes?

Final thoughts

1. Thank you for answering all the questions so far. Is there anything else you would like to tell us before we finish? Anything we haven’t covered you would like to share that you feel is important?

Thank you for your time.

***Participant Interview Schedule: Standard Intervention***

Thank you for agreeing to speak to me today. In this interview I would like to talk to you about your experiences of the programme and how you are getting on now. The interview should take about half to three quarters of an hour, but if you want to take a break or stop at any me just tell me and we can stop immediately. You don’t have to answer any question you don’t want to, so if that is the case, please just say so and we can move on to something else. I’d just like to reassure you, again, of confidentiality. Your name will not be attached to the transcripts of this interview or included in any reports of our findings from these interviews. Please feel free to add anything that you think is important but which I may not ask you about. Do you have any questions? I’d just like to check if you are happy for me to record the interview? Shall we begin?

Topics to cover

**Section 1-Motivation for the participant**

1. Can you tell me why you signed up to this study?
2. As you may know, one of the aims of this initiative is to support people to lose weight. Have you tried any weight loss methods before (and how successful were you)?
3. What made you want to lose weight on that past occasion?
4. What made you decide to participate in the programme, and did the fact it was part of a research study affect your decision? - explore why they entered the study, expectations of research study?

**Section 2-Expectations of participation**

1. What were your expectations of the programme?
2. What were you expecting from the initiative
   - Was there anything else you would like to have known?
   - Was there anything you did not understand?
   - Were your expectations met/not met?
3. How easy or hard was it to follow and understand the guidance?

- Is there anything you feel that could be done to make the guidance easier?
- Was there anything that was unclear and why?

**Section 3-Perceived advantages and disadvantages of participation**

1. How did you get on with trying to stick, day to day, to the advice that you were given on the programme?
2. Did you experience any difficulties when following the programme?
3. If so, how could these have been made more easier or practical for you?
4. Were there any tips or tasks you found particularly easy on the program?
5. Why were they easy for you?
6. Aspects of the programme that you found most helpful. Explore what these are and why
7. Any aspects you found less helpful? Explore areas for improvement, anything that could have been done differently?
8. Is there anything from the programme that you are still doing?
9. Imagine one of your friends told you they were considering whether to sign up to this programme, and they wanted your views on it. What would you tell them?
10. Imagine you were running this programme and had to improve it. What one or two improvements would you make, and why?’

**Section 4-Experiences of participation i.e., were their expectations met?**

1. You’ve told us that you expected X, Y, Z. Now let’s talk about your actual experiences. How did you get on with the programme?’

Thinking about the study programme…

1. Which parts of the programme they found most helpful?

Thinking about the one-to-one sessions with the nutritionist…

1. How did you get on with the one-to-one sessions with the nutritionist?
2. Did you attend if so, how many did you attend?
3. If you no, ask why they didn’t attend and explore any difficulties with engaging in them
4. What was/was not useful about the session)
5. Were there too many/not enough sessions, frequency?

Thinking now, about the group sessions…

1. How did you get on with the group sessions?
2. Did you attend the group sessions and if so, how many did you attend?
3. How useful did you find them?
4. If you didn't attend - why not (Explore the barriers to attending-anything that made this process difficult)
5. If yes, how did you get on with them? (What was and what was not useful)
6. Were there too many/not enough sessions, frequency? How did you feel about the group sessions ending?

Explore the components and materials used in the programme…

1. MotionWatch - Did you use it? If so, how did you get on with using it/what were your experiences? how often did you use? If no, explore why? Were there any barriers or reluctance to wearing the watch?
2. Kings’ sports app - Did you use it? If so, how did you get on with using it/what were your experiences? how often did you use? If no, explore why? Were there any barriers or reluctance to using the app?
3. Dietary Record

- What did you think about keeping a dietary record?
- How often did you engage in this?
- How did you find it?
- Did you face any problems when doing this?

1. Sleep Diary

- What did you think about keeping a sleep diary?
- How often did you engage in this?
- How did you find it?
- Were there any problems you faced when using it?

1. Weekly Weighing

- We encouraged you to weigh yourself weekly as opposed to other times; did you find this useful/helpful?
- How often did you weigh? Ask if they did keep weighing themselves to the instructed amount?
- If not why, if yes, how did they find it?
- When recording your weight how did this make you feel?

Final thoughts

- 1. Thank you for answering all the questions so far. Is there anything else you would like to tell us before we finish? Anything we haven’t covered you would like to share that you feel is important?

Thank you for your time.

**Primary outcomes**

***Practicality***

The practical feasibility of the intervention was assessed using several key recruitment metrics and retention/compliance indicators:

*Recruitment Metrics*

Five recruitment metrics were calculated:

- **Recruitment efficiency**: the proportion of participants recruited relative to the target sample size, calculated as:
  (Number recruited ÷ Target sample size) × 100.
- **Screening response rate**: the proportion of individuals who completed the screening questionnaire relative to those who initially expressed interest, calculated as:
  (Number screened ÷ Number who expressed interest) × 100.
- **Recruitment rate**: the proportion of individuals who provided consent to participate relative to those who expressed interest, calculated as:
  (Number consented ÷ Number who expressed interest) × 100.
- **Eligibility pass rate**: the proportion of screened individuals who met the inclusion criteria, calculated as:
  (Number eligible ÷ Number screened) × 100.
- **Enrolment rate**: the proportion of eligible individuals who enrolled in the trial, calculated as:
  (Number enrolled ÷ Number eligible) × 100.

*Retention and Compliance Metrics*

Participant flow through the trial was assessed using:

- **Completion rate**: the percentage of enrolled participants who completed the study, regardless of adherence to protocol, calculated as:
  (Number completed ÷ Number enrolled) × 100.
- **Compliance rate**: the proportion of enrolled participants who completed the study *and* adhered to the study protocol (see below), calculated as:
  (Number completed with adherence ÷ Number enrolled) × 100.
- **Total retention rate**: the percentage of participants who remained in the study from randomisation through to final assessment, calculated as:
  (Number retained at endpoint ÷ Number randomised) × 100.
- **Post-intervention retention rate**: the percentage of participants who completed the study after baseline (i.e., excluding dropouts before baseline assessment), calculated as:

(Number retained at endpoint ÷ Number who completed baseline) × 100.

- **Attrition rate**: the percentage of participants who withdrew or were lost to follow-up before completing the study, calculated as:

(Number withdrawn or lost ÷ Number enrolled) × 100.

*Compliance rate protocol*

In total, participants were asked to submit 14 sets of anthropometric measurements, complete two lifestyle questionnaires, one chronotype questionnaire, and four diet records, wear the Motion Watch and keep a sleep diary four times, and attend three one-to-one consultations and three group sessions. Participant compliance with the protocol was measured by the number of times they provided full and timely measurements. Participants received 1 point for each correctly submitted measurement or attended consultation, with a maximum possible score of 35 points. A partial compliance score of 0.5 points was awarded when data was submitted late, such as forgetting to submit weight or waist measurements on the same day each week; submitted early, such as requesting to wear the Motion Watch earlier due to an upcoming holiday; or when incomplete but still usable data was provided, such as submitting only two days of a diet record instead of four. If technical issues, such as problems with the diet data website or activity watches, prevented data collection, participants were still awarded 1 point if they tried to comply with the protocol. No points were given when data was either not provided or too incomplete to be usable, such as submitting only one day of a diet record. Total compliance was calculated as the sum of all full and partial points, while correct adherence to the protocol, counting only full points for correctly submitted data, was also calculated.

**Quantitative Data analyses**

Data analyses were conducted using R (v.4.3.1 ^14^). Data were assessed for normality and homoscedasticity, followed by appropriate descriptive analyses. Outliers were identified, and sensitivity analyses were performed to measure influence. Where necessary, Box-Cox transformations were applied to meet parametric assumptions and minimise outlier influence. Baseline characteristics of the sample were expressed as means and standard deviations (SD) for continuous variables and frequencies (percentages) for categorical variables. Baseline demographics and all outcome measures were summarised overall and by intervention group.

A modified intention-to-treat (mITT) approach was used, excluding participants who dropped out before baseline (n = 4). Attrition bias was assessed by comparing demographic characteristics of participants who dropped out (n = 4) with those included in analyses (n = 24) using logistic regression.

Outcomes were analysed using linear mixed-effects models (LMMs) or generalised linear mixed-effects models (GLMMs), depending on the outcome type. A two-level hierarchical structure accounted for repeated measures (post-intervention, midpoint, endpoint), improving power and adjusting for clustering at the participant level. Models were adjusted for baseline scores, stratification variables (age, sex, ethnicity), and other relevant covariates. To control for multiple comparisons, the Benjamini-Hochberg (BH) procedure was applied within each model, followed by a False Discovery Rate (FDR) correction across all models, with an alpha threshold of 0.05 applied in each instance.

The proportion of missing data for all variables (**Supplementary Table 1**) was assessed. LMMs/GLMMs, applying maximum likelihood estimation (MLE/REML), accounted for missing data without imputation or participant exclusion, in line with the mITT principle. To assess whether missing data followed a Missing at Random (MAR) pattern and to justify LMM/GLMM use, logistic regression models were used, incorporating age, gender, baseline measurements, and intervention group as predictors of missingness.

**RESULTS**

Exploratory inferential analyses are presented for transparency only and should not be interpreted as evidence of intervention effectiveness, as this feasibility trial was not powered for hypothesis testing.

**Exploratory inferential analyses of objective and subjective physical activity outcomes**

For overall PA (MET minutes per week), no clear main effect of intervention was observed (β = 0.09, SE = 0.12, t = 0.81, p = 0.42). A main effect of time was observed (β = 0.15, SE = 0.72, t = 2.11, p = 0.04), along with a time-by-intervention interaction in the fully adjusted model (β = 0.12, SE = 0.10, t = 2.29, p = 0.02). PA levels in the sleep-enhanced intervention (SEI) group increased over the course of the intervention, with higher activity observed at Week 5 (β = 0.23, SE = 0.12, z = 1.92, p = 0.055) and Week 9 relative to baseline (β = 0.28, SE = 0.12, z = 2.12, p = 0.03) and the standard intervention (SI) group (β = 0.34, SE = 0.12, z = 2.80, p = 0.005). By Week 14, activity levels in the SEI group had declined, although estimates remained higher than those in the SI group (β = 0.21, SE = 0.12, z = 1.79, p = 0.07). The SI group showed little change in overall PA across time.

No clear main effects of intervention or time were observed for sedentary behaviour (intervention: β = −0.01, SE = 0.04, t = −0.28, p = 0.78; time: β = −0.05, SE = 0.04, t = −1.10, p = 0.27), moderate PA (intervention: β = 0.07, SE = 0.09, t = 0.86, p = 0.39; time: β = 0.09, SE = 0.06, t = 1.51, p = 0.13), or vigorous PA (intervention: β = 0.14, SE = 0.26, t = 0.55, p = 0.58; time: β = 0.16, SE = 0.13, t = 1.19, p = 0.23). A time-by-intervention interaction was observed for vigorous activity (β = 0.41, SE = 0.20, t = 2.06, p = 0.03), with higher vigorous activity in the SEI group compared with the SI group at Week 9 only (β = 0.24, SE = 0.09, z = 2.76, p = 0.006).

For self-reported PA (IPAQ-SF MET minutes), no clear main effects of intervention or time were observed (intervention: β = 0.42, SE = 0.34, t = 1.20, p = 0.23; time: β = 0.69, SE = 0.37, t = 1.85, p = 0.06). However, a time-by-intervention interaction was detected (β = 1.17, SE = 0.51, t = 2.28, p = 0.02). Estimates indicated higher reported activity at endpoint (Week 14) in the SI group compared with the SEI group (β = 0.76, SE = 0.97, z = 1.97, p = 0.04). An increase from baseline to endpoint was also observed within the SI group (β = 0.69, SE = 0.37, z = 1.85, p = 0.06).

None of the reported results remained after FDR adjustment.

**Exploratory inferential analyses of diet and eating behaviour outcomes**

For total energy intake, no clear main effects of intervention or time were observed, and no time-by-intervention interaction was detected in the fully adjusted model (intervention: β = −1.28, SE = 2.05, t = −0.62, p = 0.53; time: β = −3.44, SE = 1.87, t = −1.90, p = 0.07; interaction: β = −3.00, SE = 2.89, t = −1.04, p = 0.31). Energy intake decreased over time in both groups, with larger reductions observed in the sleep-enhanced intervention (SEI) group at week 14 relative to baseline.

For diet quality outcomes, no clear main effects or interactions were observed for either Healthy Eating Index (HEI) or Nutrient Adequacy Score (NAS) (HEI: intervention: β = 0.27, SE = 3.12, t = 0.09, p = 0.93; time: β = 0.14, SE = 2.70, t = 0.05, p = 0.80; interaction: β = 1.06, SE = 4.03, t = 0.26, p = 0.79; NAS: intervention: β = 0.02, SE = 0.05, z = 0.56, p = 0.57; time: β = 0.07, SE = 0.04, z = 1.45, p = 0.15; interaction: β = 0.01, SE = 0.07, z = 0.14, p = 0.88).

For eating behaviour, no clear main effects or interactions were observed for eating onset (intervention: β = 0.003, SE = 0.12, t = 0.003, p = 0.98; time: β = −0.01, SE = 0.10, t = −0.12, p = 0.91; interaction: β = −0.03, SE = 0.15, t = −0.22, p = 0.83). For eating offset, a time-by-intervention interaction was observed (β = −0.21, SE = 0.12, t = −2.21, p = 0.03), with earlier cessation of eating in the SEI group at week 9 compared with the standard intervention (SI) group (β = −0.16, SE = 0.05, t = −2.73, p = 0.03). No consistent differences were observed at other timepoints.

Similarly, for eating window, no clear main effects were observed, but a time-by-intervention interaction was detected (intervention: β = −5.76, SE = 7.20, t = −0.78, p = 0.43; time: β = −6.36, SE = 6.14, t = −1.04, p = 0.30; interaction: β = −20.74, SE = 9.03, t = −2.30, p = 0.03), with a shorter eating window in the SEI group at week 9 compared with the SI group (β = −0.95, SE = 0.37, t = −2.58, p = 0.01).

None of the reported results remained after FDR adjustment.

**Exploratory inferential analyses of sleep outcomes**

For actigraphy-derived measures, no clear main effects or interactions were observed for overall sleep quality, social jet lag or sleep debt (sleep quality: intervention: β = 0.34, SE = 0.46, z = 0.75, p = 0.45; time: β = 0.001, SE = 0.04, z = 0.04, p = 0.97; interaction: β = 0.01, SE = 0.05, z = 0.21, p = 0.83; social jet lag: intervention: β = 0.17, SE = 0.33, z = 0.52, p = 0.61; time: β = 0.002, SE = 0.02, z = 0.09, p = 0.93; interaction: β = 0.01, SE = 0.03, z = 0.18, p = 0.86; sleep debt: intervention: β = −0.35, SE = 2.20, t = −0.16, p = 0.87; time: β = −0.02, SE = 0.09, t = −0.23, p = 0.82; interaction: β = −0.03, SE = 0.13, t = −0.19, p = 0.85).

For self-reported outcomes, no clear main effects or interactions were observed for sleep disturbance or daytime sleepiness (sleep disturbance: intervention: β = −0.79, SE = 1.08, t = −0.73, p = 0.47; time: β = −0.09, SE = 0.07, t = −1.22, p = 0.23; interaction: β = −0.07, SE = 0.10, t = −0.77, p = 0.45; daytime sleepiness: intervention: β = −0.12, SE = 0.74, t = −0.17, p = 0.87; time: β = −0.05, SE = 0.65, t = −0.21, p = 0.83; interaction: β = −0.09, SE = 0.07, t = −1.17, p = 0.26).

For sleep hygiene, a main effect of time was observed (β = −0.20, SE = 0.10, t = −2.03, p = 0.049), with lower scores at week 14 relative to baseline in both intervention groups (SI: β = −0.31, SE = 0.10, t = −2.98, p = 0.03; SEI: β = −0.32, SE = 0.10, t = −2.56, p = 0.03).

None of the reported results remained after FDR adjustment.

**Exploratory inferential analyses of anthropometric outcomes**

For body mass index (BMI), no clear main effect of intervention was observed (β = −2.52 × 10⁻⁵, SE = 1.89 × 10⁻⁵, t = −1.39, p = 0.17). A main effect of time and a time-by-intervention interaction were observed (time: β = −2.13 × 10⁻⁶, SE = 2.49 × 10⁻⁷, t = −8.55, p < 0.001; interaction: β = 1.41 × 10⁻⁶, SE = 3.59 × 10⁻⁷, t = 3.93, p < 0.001). BMI decreased over time in both groups, with earlier reductions observed in the standard intervention (SI) group.

For waist circumference, no clear main effect of intervention was observed (β = −0.004, SE = 0.01, t = −0.31, p = 0.76). A main effect of time and interaction were detected (time: β = −0.003, SE = 2.56 × 10⁻⁴, t = −9.78, p < 0.001; interaction: β = 0.001, SE = 3.72 × 10⁻⁴, t = 3.36, p < 0.001). Reductions occurred in both groups, with larger decreases observed earlier in the SI group.

**Exploratory inferential analyses of mental well-being and workplace outcomes**

For anxiety symptoms, no clear main effects of intervention or time were observed, but a time-by-intervention interaction was detected (β = −1.10, SE = 0.32, z = −3.40, p < 0.001), with lower anxiety scores in the SEI group at week 14 relative to baseline and the SI group (baseline comparison: β = −1.06, SE = 0.28, z = −3.76, p < 0.001; between-group comparison at week 14: β = −0.96, SE = 0.28, z = −3.45, p = 0.002).

For depressive symptoms, no clear main effects or interactions were observed (intervention: β = −0.25, SE = 0.22, z = −1.17, p = 0.24; time: β = 0.04, SE = 0.15, z = 0.29, p = 0.77; interaction: β = −0.39, SE = 0.27, z = −1.45, p = 0.15).

For workplace outcomes, no clear main effects or interactions were observed for work engagement, job satisfaction or burnout. For social support and coping, a stable between-group difference was observed (intervention: β = −3.99, SE = 1.40, t = −2.86, p = 0.007), with lower scores in the SEI group across time.

Only observed differences in anxiety outcomes remained after FDR adjustment.

| SUPPLEMENTARY TABLE 1. Percentage of missing data for all outcome measures throughout the duration of the study for all participants that actively started the intervention (n=24) | | | |
| --- | --- | --- | --- |
| **Outcomes** | **Missing data (%)** | | |
|  | **All Participants**  **(N=24)** | **Standard Intervention**  **(N=12)** | **Sleep-enhanced Intervention**  **(N=12)** |
| **PHYSICAL ACTIVITY ACTIGRAPHY ^(a)^** | | | |
| **Outcomes: Sedentary Behaviour; Overall Physical Activity** | | | |
| Week 1: Baseline | 0 | 0 | 0 |
| Week 5 | 13 | 17 | 9 |
| Week 9 | 13 | 8 | 18 |
| Week 14: Endpoint | 4 | 8 | 0 |
| **DIET AND NUTRITION ^(b)^** | | | |
| **Outcomes: Energy Intake, Healthy Eating Index Score, Nutrient Adequacy Score, Eating onset, Eating offset, Eating window** | | | |
| Week 1: Baseline | 4 | 0 | 9 |
| Week 5 | 9 | 8 | 9 |
| Week 9 | 22 | 25 | 18 |
| Week 14: Endpoint | 9 | 0 | 18 |
| **SLEEP ACTIGRAPHY ^(a)^** | | | |
| **Outcomes: Sleep Quality, Sleep duration, Sleep Efficiency, Sleep Latency, Sleep Fragmentation, Social Jet Lag & Sleep Debt – all total week** | | | |
| Week 1: Baseline | 0 | 0 | 0 |
| Week 5 | 13 | 17 | 0 |
| Week 9 | 13 | 8 | 18 |
| Week 14: Endpoint | 4 | 8 | 0 |
| **ANTHROPOMETRY** | | | |
| **Body Mass Index (BMI)** |  |  |  |
| Week 1: Baseline | 0 | 0 | 0 |
| Week 2 | 0 | 0 | 0 |
| Week 3 | 0 | 0 | 0 |
| Week 4 | 4 | 8 | 0 |
| Week 5 | 9 | 17 | 0 |
| Week 6 | 9 | 8 | 9 |
| Week 7 | 0 | 0 | 0 |
| Week 8 | 0 | 0 | 0 |
| Week 9 | 4 | 8 | 0 |
| Week 10 | 9 | 8 | 9 |
| Week 11 | 17 | 8 | 18 |
| Week 12 | 13 | 17 | 9 |
| Week 13 | 17 | 8 | 27 |
| Week 14: Endpoint | 9 | 8 | 9 |
| **Waist Circumference (cm)** |  |  |  |
| Week 1: Baseline | 0 | 0 | 0 |
| Week 2 | 4 | 0 | 9 |
| Week 3 | 0 | 0 | 0 |
| Week 4 | 4 | 8 | 0 |
| Week 5 | 9 | 17 | 0 |
| Week 6 | 9 | 8 | 9 |
| Week 7 | 0 | 0 | 0 |
| Week 8 | 0 | 0 | 0 |
| Week 9 | 4 | 8 | 0 |
| Week 10 | 9 | 8 | 9 |
| Week 11 | 17 | 8 | 27 |
| Week 12 | 13 | 17 | 9 |
| Week 13 | 17 | 8 | 27 |
| Week 14: Endpoint | 9 | 8 | 9 |
| **MENTAL HEALTH AND WELL-BEING QUESTIONNAIRES** | | | |
| **Outcomes: Depressive symptomatology ^(c)^ and anxiety symptomatology ^(d)^** | | | |
| Week 1: Baseline | 0 | 0 | 0 |
| Week 14: Endpoint | 4 | 8 | 0 |
| **WORK-RELATED QUESTIONNAIRES** | | | |
| **Outcomes: Work engagement ^(e)^, job satisfaction ^(f)^, social support ^(g)^ and coping, burnout ^(h)^** | | | |
| Week 1: Baseline | 0 | 0 | 0 |
| Week 14: Endpoint | 4 | 8 | 0 |
| **SLEEP AND PHYSICAL ACTIVITY QUESTIONNAIRES** | | | |
| **Outcomes: Sleep disturbance ^(i)^, daytime sleepiness ^(j)^, sleep hygiene ^(k)^ and physical activity ^(l)^** | | | |
| Week 1: Baseline | 0 | 0 | 0 |
| Week 14: Endpoint | 4 | 8 | 0 |
| **(a)** Data acquired through a wearable device: MotionWatch 8. **(b)** Estimated from online food diaries using Intake24 software. **(c)** Assessed using the Patient Health Questionnaire-9 ^9^. **(c)** Assessed using the Generalised Anxiety Disorder Questionnaire (GAD-7) ^8^. **(e)** Assessed using the Short Utrecht Work Engagement Scale (UWES-9) ^10^**. (f)** Assessed using the Short Index of Job Satisfaction (SIJS) ^11^. **(g)** Assessed using State Self-Control Capacity Scale (SSCCS) ^12^. **(h)** Assessed using the Oldenberg Burnout Inventory (OLBI) ^13^. **(i)** Assessed using the Patient Reported Outcomes Measurement Information System (PROMIS) sleep/wake disturbances scale ^5^. **(j)** Assessed using the Epworth Sleepiness Scale (ESS) **^6^**. **(k)** Assessed using the Sleep Hygiene Index (SHI) **^7^**. **(l)** Assessed using the shortened version of the International Physical Activity Questionnaire (IPAQ-SF) ^4^. | | | |

| SUPPLEMENTARY TABLE 2. Demographic characteristics of participants who took part in qualitative interviews (n=13) | | | | | |
| --- | --- | --- | --- | --- | --- |
| **Participant** | **Sex** | **Age** | **Ethnicity** | **Baseline BMI** | **Intervention Group** |
| 1 | Female | 26-35 | White Latina | 29.16 | SEI |
| 2 | Female | 26-35 | British | 27.82 | SI |
| 3 | Male | 45-64 | Brazilian | 26.74 | SEI |
| 4 | Female | 26-35 | British | 30.92 | SI |
| 5 | Female | 45-64 | African | 36.21 | SI |
| 6 | Female | 45-64 | Portuguese | 44.74 | SEI |
| 7 | Female | 45-64 | Black British | 25.72 | SEI |
| 8 | Female | 36-44 | British | 26.95 | SEI |
| 9 | Female | 45-64 | European | 30.43 | SI |
| 10 | Male | 26-35 | Latin American | 26.03 | SEI |
| 11 | Female | 45-64 | Canadian | 39.32 | SEI |
| 12 | Female | 45-64 | British | 31.73 | SEI |
| 13 | Female | 26-35 | American | 31.36 | SI |
| **Abbreviations**: BMI, body mass index; SEI, sleep-enhanced intervention; SI, standard intervention. | | | | | |

| SUPPLEMENTARY TABLE 3. Within-group associations for physical activity outcomes throughout the study duration (n = 24) | | | |
| --- | --- | --- | --- |
| **Outcome** | **Baseline vs. Week 5** | **Baseline vs. Week 9** | **Baseline vs. Week 14: Endpoint** |
| **STANDARD INTERVENTION** | | | |
| Sedentary behaviour mean percentage change (SD)^1^ | 8.44 (14.80) | 5.86 (11.16) | -0.20 (5.58) |
| Overall physical activity mean percentage change (SD)^1^ | -9.58 (11.32) | -13.99 (17.22) | -6.67 (35.89) |
| **SLEEP-ENHANCED INTERVENTION** | | | |
| Sedentary behaviour mean percentage change (SD)^1^ | 0.55 (9.88) | 2.08 (11.78) | -4.67 (4.89) |
| Overall physical activity mean percentage change (SD)^1^ | 8.49 (34.46) | 9.96 (19.00) | 2.86 (27.17) |
| Values represent mean (SD) of non-missing values from all participants that started the intervention. N=4 enrolled participants dropped out prior to the baseline assessment (week 1).  ^1^ Values expressed as mean percentage change relative to baseline measure (week 1).  Physical activity measures were objectively measured using the MotionWatch 8© actigraphy system. Activity (MET) minutes were calculated by multiplying the duration of each activity (in minutes) by its corresponding Metabolic Equivalent of Task (MET) value. Total MET minutes represent the sum of all activities over the specified period ^15^. | | | |

| SUPPLEMENTARY TABLE 4. Within-group associations for diet and nutrition outcomes throughout the study duration (n = 24) | | | |
| --- | --- | --- | --- |
| **Outcome** | **Baseline vs. Week 5** | **Baseline vs. Week 9** | **Baseline vs. Week 14: Endpoint** |
| **STANDARD INTERVENTION** | | | |
| Energy intake (Kcal) mean percentage change (SD)^1^ | 2.83 (38.83) | -16.83 (19.75) | -10.84 (28.40) |
| Healthy Eating Index ^(a)^ mean percentage change (SD)^1^ | 0.90 (12.98) | -0.02 (19.11) | -0.46 (17.09) |
| Nutrient Adequacy Score ^(b)^ mean percentage change (SD) ^1^ | 9.54 (40.14) | -2.20 (25.14) | 0.92 (35.72) |
| Eating onset ^(c)^ mean percentage change (SD) ^1^ | 0.65 (13.60) | 0.05 (24.63) | 2.06 (16.16) |
| Eating offset ^(d)^ mean percentage change (SD) ^1^ | 14.01 (28.36) | 22.12 (46.71) | 12.19 (38.32) |
| Eating window mean percentage change (SD)^1^ | 14.01 (28.34) | 22.12 (46.71) | 12.19 (38.33) |
| **SLEEP-ENHANCED INTERVENTION** | | | |
| Energy intake (Kcal) mean percentage change (SD)^1^ | -9.73 (22.67) | -11.49 (37.39) | -19.39 (36.48) |
| Healthy Eating Index ^(a)^ mean percentage change (SD)^1^ | 3.80 (22.13) | -6.66 (16.39) | -14.64 (17.27) |
| Nutrient Adequacy Score ^(b)^ mean percentage change (SD)^1^ | -1.87 (13.11) | -7.79 (22.97) | -4.28 (20.73) |
| Eating onset ^(c)^ mean percentage change (SD)^1^ | 5.36 (3.74) | 3.96 (10.14) | 0.24 (8.13) |
| Eating offset ^(d)^ mean percentage change (SD)^1^ | -6.73 (7.95) | -11.03 (17.01) | -3.04 (12.77) |
| Eating window mean percentage change (SD)^1^ | -6.73 (7.95) | -11.03 (17.00) | -3.04 (12.77) |
| Values represent mean (SD) of non-missing values from all participants that started the intervention. N=4 enrolled participants dropped out prior to the baseline assessment (week 1).  ^1^ Values expressed as mean percentage change relative to baseline measure (week 1).  Dietary intake was assessed using INTAKE24, an online dietary recall tool (<https://intake24.co.uk/>). **(a)** The Healthy Eating Index (HEI) score was calculated based on dietary intake, assessing adherence to UK dietary guidelines ^16^, as previously described ^17^. Component scores were summed to generate a total HEI score, with higher values indicating better diet quality. **(b)** The Nutrient Adequacy Score (NAS) was calculated based on nutrient and mineral intake, reflecting the proportion of essential nutrients meeting UK recommended levels ^18^. Higher scores indicate greater nutrient adequacy ^19^. **(c)** Refers to the recorded time when participants start eating for the day. **(d)** Refers to the recorded time when participants stop eating for the day. | | | |

| SUPPLEMENTARY TABLE 5. Within-group associations for sleep outcomes throughout the study duration (n = 24) | | | |
| --- | --- | --- | --- |
| **Outcome** | **Baseline vs. Week 5** | **Baseline vs. Week 9** | **Baseline vs. Week 14: Endpoint** |
| **STANDARD INTERVENTION** | | | |
| Sleep Quality ^(a)^ absolute change (SD)^1^ | 0.48 (0.40) | 0.37 (0.30) | 0.18 (0.15) |
| Sleep Duration (h) absolute change (SD)^1^ | 0.82 (0.46) | 0.56 (0.36) | 0.35 (0.23) |
| Sleep Latency (min) absolute change (SD)^1^ | 0.15 (0.29) | 0.23 (0.31) | 0.21 (0.19) |
| Sleep Efficiency percentage change  (SD)^2^ | 0.14 (4.03) | -3.11 (6.87) | - 2.83 (4.82) |
| Sleep Fragmentation percentage change (SD)^2^ | 3.08 (29.74) | 18.33 (31.88) | 25.74 (19.72) |
| Social Jetlag ^(b)^ absolute change (SD)^2^ | 0.83 (0.66) | 1.14 (1.42) | 0.63 (0.45) |
| Sleep Debt ^(c)^ absolute change (SD)^2^ | 5.66 (2.13) | 3.75 (2.32) | 2.60 (1.54) |
| **SLEEP-ENHANCED INTERVENTION** | | | |
| Sleep Quality ^(a)^ absolute change (SD)^1^ | 0.47 (0.49) | 0.49 (0.40) | 0.63 (0.38) |
| Sleep Duration (h) absolute change (SD)^1^ | 0.57 (0.36) | 0.59 (0.57) | 0.53 (0.34) |
| Sleep Latency (min) absolute change (SD)^1^ | 0.05 (0.17) | 0.17 (0.22) | 0.18 (0.19) |
| Sleep Efficiency percentage change  (SD)^2^ | -2.41 (7.04) | -2.47 (5.63) | -4.24 (6.10) |
| Sleep Fragmentation percentage change (SD)^2^ | 5.20 (30.01) | 7.68 (16.37) | 12.70 (26.83) |
| Social Jetlag ^(b)^ absolute change (SD)^2^ | 1.07 (1.04) | 0.85 (0.33) | 0.70 (0.64) |
| Sleep Debt ^(c)^ absolute change (SD)^2^ | 3.86 (2.16) | 3.72 (3.25) | 4.21 (2.37) |
| Values represent mean (SD) of non-missing values from all participants that started the intervention. N=4 enrolled participants dropped out prior to the baseline assessment (week 1).  ^1^ Values expressed as absolute change from baseline (week 1) to endpoint (week 14).  ^2^ Values expressed as mean percentage change relative to baseline measure (week 1).  Sleep outcome measures were objectively measured using the MotionWatch 8© actigraphy system. **(a)** A composite MW8 sleep quality score was created by averaging the standardised duration, efficiency, and fragmentation scores across the whole week. The fragmentation score was multiplied by −1 prior to averaging ^20,21^. Higher composite scores represent better sleep quality. **(b)** Social jet lag was operationalised as the difference between weekday and weekend sleep midpoints. It reflects the misalignment between the individual's biological clock and social or work-related schedules, with greater differences indicating higher levels of social jet lag ^22,23^. **(c)** Refers to the cumulative difference between sleep duration and the recommended amount (8h). A positive sleep debt indicates insufficient sleep compared to the recommended amount ^24^. | | | |

| SUPPLEMENTARY TABLE 6. Within-group associations for sleep and physical activity self-report outcomes at baseline and  endpoint (n = 24) | |
| --- | --- |
| **Outcome** | **Baseline vs. Week 14: Endpoint** |
| **STANDARD INTERVENTION** | |
| Sleep disturbance mean percentage score change **^(a)^** | -1.13 (3.61) |
| Daytime sleepiness mean percentage score change ^(b)^ | 0.67 (2.42) |
| Sleep hygiene mean percentage score change ^(c)^ | -2.60 (5.21) |
| Physical activity mean percentage score change ^(d)^ | -131 (2,510) |
| **SLEEP-ENHANCED INTERVENTION** | |
| Sleep disturbance mean percentage score change **^(a)^** | -0.10 (3.14) |
| Daytime sleepiness mean percentage score change ^(b)^ | -0.45 (2.16) |
| Sleep hygiene mean percentage score change ^(c)^ | -2.61 (4.11) |
| Physical activity mean percentage score change ^(d)^ | -738 (987) |
| Values represent mean (SD) of non-missing values from all participants that started the intervention. N=4 enrolled participants dropped out prior to the baseline assessment (week 1). All values expressed as mean percentage change from baseline (week 1) to endpoint (week 14).  **(a)** Assessed using the Patient Reported Outcomes Measurement Information System (PROMIS) sleep/wake disturbances scale ^5^. **(b)** Expressed as percentage change from baseline (week 1) to endpoint (week 14). **(c)** Assessed using the Epworth Sleepiness Scale (ESS) **^6^**. **(d)** Assessed using the Sleep Hygiene Index (SHI) **^7^. (e)** Assessed using the shortened version of the International Physical Activity Questionnaire (IPAQ-SF) ^4^. | |

| SUPPLEMENTARY TABLE 7. Within-group associations for anthropometric health outcomes throughout the study duration (n = 24) | | |
| --- | --- | --- |
| **Week Comparison** | **Body Mass Index (BMI) mean % change (SD)^1^** | **Waist Circumference (cm)**  **mean % change (SD)^1^** |
| **STANDARD INTERVENTION** | | |
| Baseline vs. Week 2 | 0.00 (0.00) | 0.00 (0.00) |
| Baseline vs. Week 3 | -0.45 (1.52) | -0.97 (1.40) |
| Baseline vs. Week 4 | -0.76 (1.62) | -0.88 (2.41) |
| Baseline vs. Week 5 | -0.93 (1.50) | -1.97 (2.25) |
| Baseline vs. Week 6 | -1.39 (1.51) | -1.60 (1.77) |
| Baseline vs. Week 7 | -1.43 (1.56) | -2.14 (2.17) |
| Baseline vs. Week 8 | -1.27 (1.72) | -2.35 (1.88) |
| Baseline vs. Week 9 | -1.57 (1.16) | -2.44 (1.85) |
| Baseline vs. Week 10 | -1.96 (1.14) | -2.58 (2.05) |
| Baseline vs. Week 11 | -2.29 (1.39) | -2.88 (2.00) |
| Baseline vs. Week 12 | -2.14 (1.35) | -3.10 (2.13) |
| Baseline vs. Week 13 | -2.39 (1.53) | -3.34 (2.52) |
| Baseline vs. Week 14: Endpoint | -2.20 (1.59) | -3.60 (2.17) |
| **SLEEP-ENHANCED INTERVENTION** | | |
| Baseline vs. Week 2 | 0.00 (0.00) | 0.00 (0.00) |
| Baseline vs. Week 3 | -0.17 (0.96) | -0.20 (1.56) |
| Baseline vs. Week 4 | -0.02 (0.97) | -0.94 (2.25) |
| Baseline vs. Week 5 | -0.64 (1.25) | -1.08 (2.41) |
| Baseline vs. Week 6 | -0.47 (1.19) | -1.71 (2.96) |
| Baseline vs. Week 7 | -0.19 (1.69) | -1.00 (2.26) |
| Baseline vs. Week 8 | -0.24 (1.58) | -1.54 (2.75) |
| Baseline vs. Week 9 | -0.28 (1.37) | -1.61 (3.28) |
| Baseline vs. Week 10 | -0.23 (1.47) | -1.55 (3.23) |
| Baseline vs. Week 11 | -0.88 (1.59) | -1.77 (3.37) |
| Baseline vs. Week 12 | -0.20 (2.10) | -1.74 (3.53) |
| Baseline vs. Week 13 | -0.43 (2.22) | -1.76 (3.67) |
| Baseline vs. Week 14: Endpoint | -0.84 (1.32) | -1.27 (4.16) |
| ^1^ Values expressed as mean percentage change relative to baseline measure (week 1). Values represent mean (SD) of non-missing values from all participants that started the intervention. N=4 enrolled participants dropped out prior to the baseline assessment (week 1). | | |

| SUPPLEMENTARY TABLE 8. Within-group associations for well-being- and workplace-related health outcomes throughout the study duration (n = 24) | |  |
| --- | --- | --- |
| **Outcome** | **Baseline vs. Week 14: Endpoint** |  |
| **MENTAL HEALTH AND WELL-BEING** | | |
| **STANDARD INTERVENTION** | | |
| Depressive symptomatology mean absolute score change **^(a)^** | 0.33 (4.72) |  |
| Anxiety symptomatology mean absolute score change ^(b)^ | 0.31 (3.11) |  |
| **SLEEP-ENHANCED INTERVENTION** | | |
| Depressive symptomatology mean absolute score change **^(a)^** | -1.34 (2.82) |  |
| Anxiety symptomatology mean absolute score change ^(b)^ | -2.93 (2.71) |  |
| **WORKPLACE-RELATED OUTCOMES** | | |
| **STANDARD INTERVENTION** | |  |
| Work engagement mean percentage score change **^(c)^** | 11.23 (26.01) |  |
| Job satisfaction mean percentage score change ^(d)^ | 1.83 (15.22) |  |
| Social support and coping mean percentage score change ^(e)^ | 9.14 (33.43) |  |
| Burnout mean percentage score change ^(f)^ | -3.43 (19.12) |  |
| **SLEEP-ENHANCED INTERVENTION** | | |
| Work engagement mean percentage score change **^(c)^** | 3.36 (28.01) |  |
| Job satisfaction mean percentage score change ^(d)^ | -10.24 (13.54) |  |
| Social support and coping mean percentage score change ^(e)^ | 3.54 (48.75) |  |
| Burnout mean percentage score change ^(f)^ | -3.01 (10.12) |  |
| ^1^ Values expressed as mean percentage change relative to baseline measure (week 1). Values represent mean (SD) of non-missing values from all participants that started the intervention. N=4 enrolled participants dropped out prior to the baseline assessment (week 1).  **(a)** Assessed using the Patient Health Questionnaire-9 (PHQ-9) ^9^. **(b)** Assessed using the Generalised Anxiety Disorder Questionnaire (GAD-7) ^8^. **(c)** Assessed using the Short Utrecht Work Engagement Scale (UWES-9) ^10^**. (d)** Assessed using the Short Index of Job Satisfaction (SIJS) ^11^. **(e)** Assessed using State Self-Control Capacity Scale (SSCCS) ^12^. **(f)** Assessed using the Oldenberg Burnout Inventory (OLBI) ^13^.  Note: Mental wellbeing and workplace wellbeing were only assessed at two timepoints, i.e., baseline and endpoint. | | |

**Preliminary Sleep Actigraphy Outcomes**

**Supplementary Figure 1. Preliminary findings on the impact of the WHOLE feasibility trial intervention on objective sleep outcomes.**

**(A) Impact of the intervention on total sleep duration:** There was no significant main effect of the intervention or time, nor was there a significant interaction between intervention and time on total sleep duration in a fully adjusted model (intervention: β = 0.04, SE = 0.23, t = 0.19, *p* = .86; time: β = **0**.003, SE = 0.02, t = **0**.17, *p* = .87; intervention x time: β = **0**.007, SE = 0.02, t = **0**.30, *p* = .**77**). **Analytical approach: Linear mixed model, including a** random intercept for participant ID. **(B) Impact of the intervention on sleep latency:** There was no significant main effect of the intervention or time, nor was there a significant interaction between intervention and time on sleep latency in a fully adjusted model (intervention: β = **-**0.**22**, SE = 0.38, t = -0.57, *p* = .57; time: β = **0**.04, SE = 0.02, t = 1.72, *p* = .09; intervention x time: β = **0**.03, SE = 0.03, t = **0**.88, *p* = .**38**). **Analytical approach: Generalised linear mixed models using a Gamma distribution and a log link function, including a** random intercept for participant ID**. (C) Impact of the intervention on sleep efficiency:** There was no significant main effect of the intervention or time, nor was there a significant interaction between intervention and time on sleep efficiency in a fully adjusted model (intervention: β = 0.06, SE = 0.12, z = 0.45, *p* = .65; time: β =- **0**.02, SE = 0.01, t = -1.81, *p* = .07; intervention x time: β = -**0**.002, SE = 0.01, t = -**0**.18, *p* = .**86**). **Analytical approach: G**eneralised linear mixed model with a beta regression distribution and a random intercept for participant ID. **(D) Impact of the intervention on sleep fragmentation:** There was no significant main effect of the intervention or time on sleep fragmentation in a fully adjusted model (intervention: β = 0.03, SE = 0.10, z = 0.31, *p* = .76; time: β = **0**.03, SE = 0.01, t = 1.79, *p* = .07). However, there was a significant interaction between the intervention and time (β = -**0**.0**3**, SE = 0.01, t = -3.43, *p* = .**001**). Further analyses showed that participants in the standard intervention (SI) group experienced more fragmented sleep at week 14 than participants in the sleep-enhanced intervention (SEI) group (β = **0**.24, SE = 0.11, t = 2.22, *p* = .**02). Analytical approach:** Generalised linear mixed model with a beta regression distribution and a random intercept for participant ID.

All models were controlled for baseline levels of the respective outcome, age, sex and ethnicity. The *p* values displayed here are not adjusted across models for multiple outcomes, though Benjamini-Hochberg (BH) correction was applied within each model. All models were further adjusted using False Discovery Rate (FDR) correction, but none of the results reported survived this adjustment. ** p < .05.*

**Note: Exploratory inferential analyses presented here are for transparency only and should not be interpreted as evidence of intervention effectiveness, as this feasibility trial was not powered for hypothesis testing.**


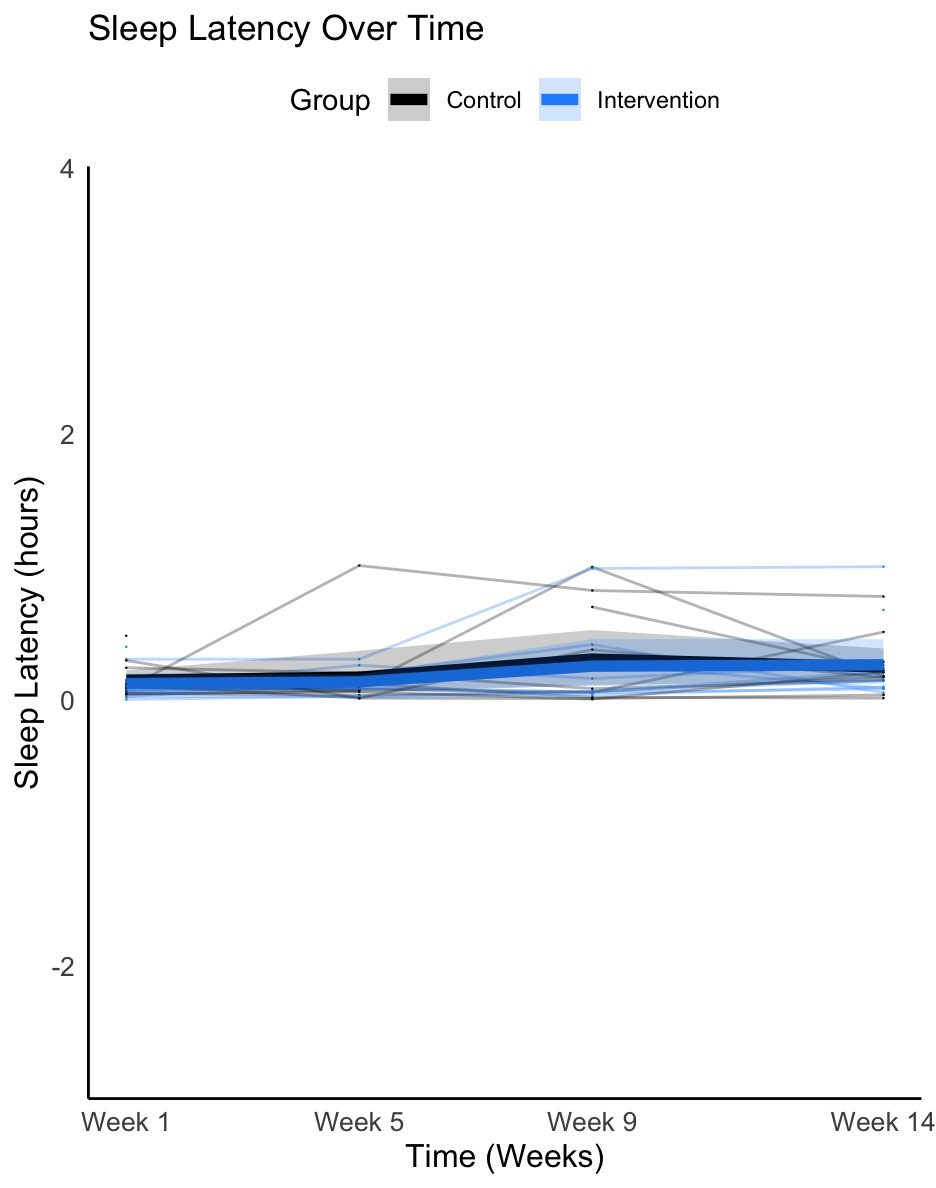

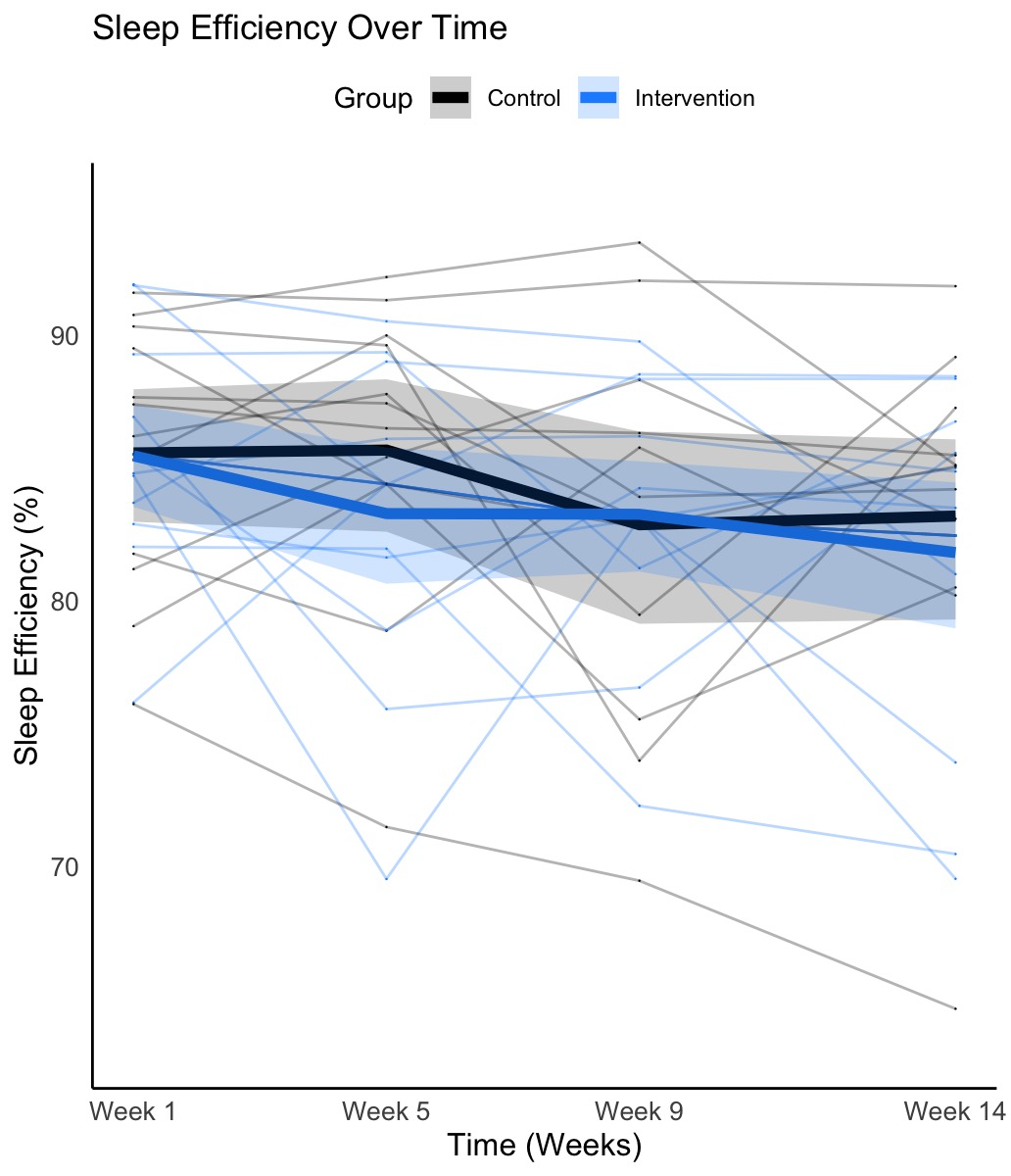

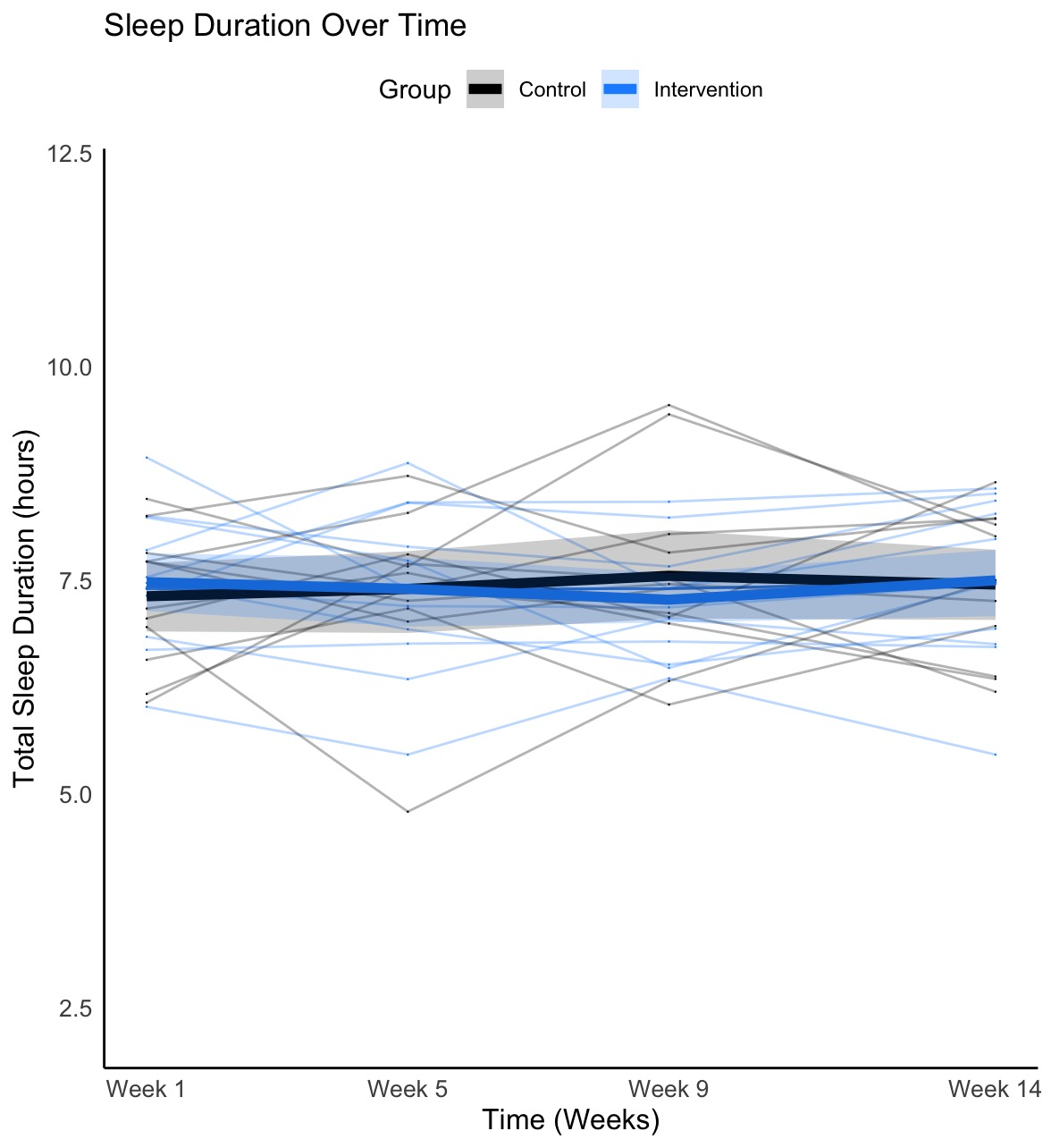

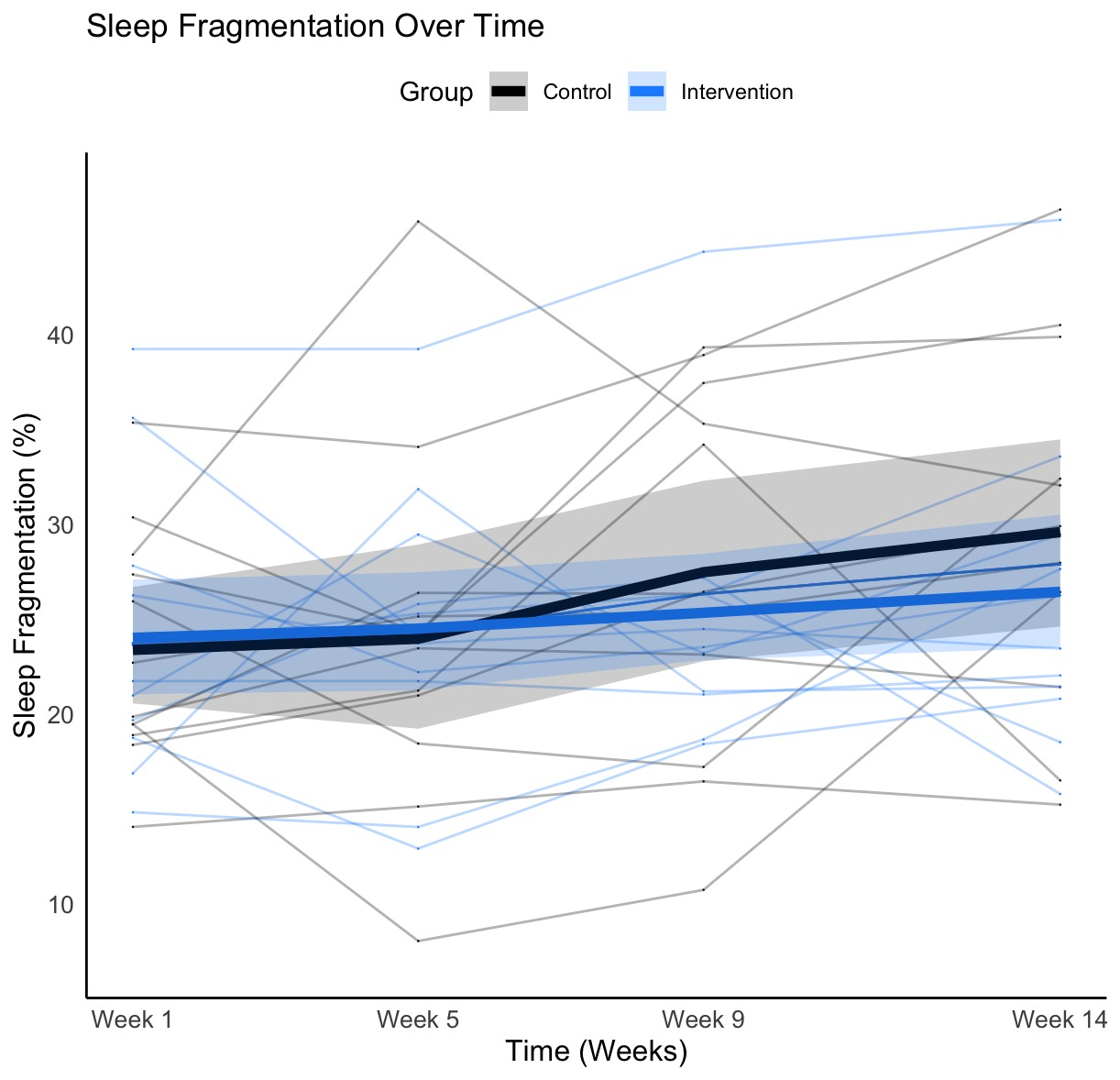


Timepoint

Timepoint

Timepoint

Timepoint


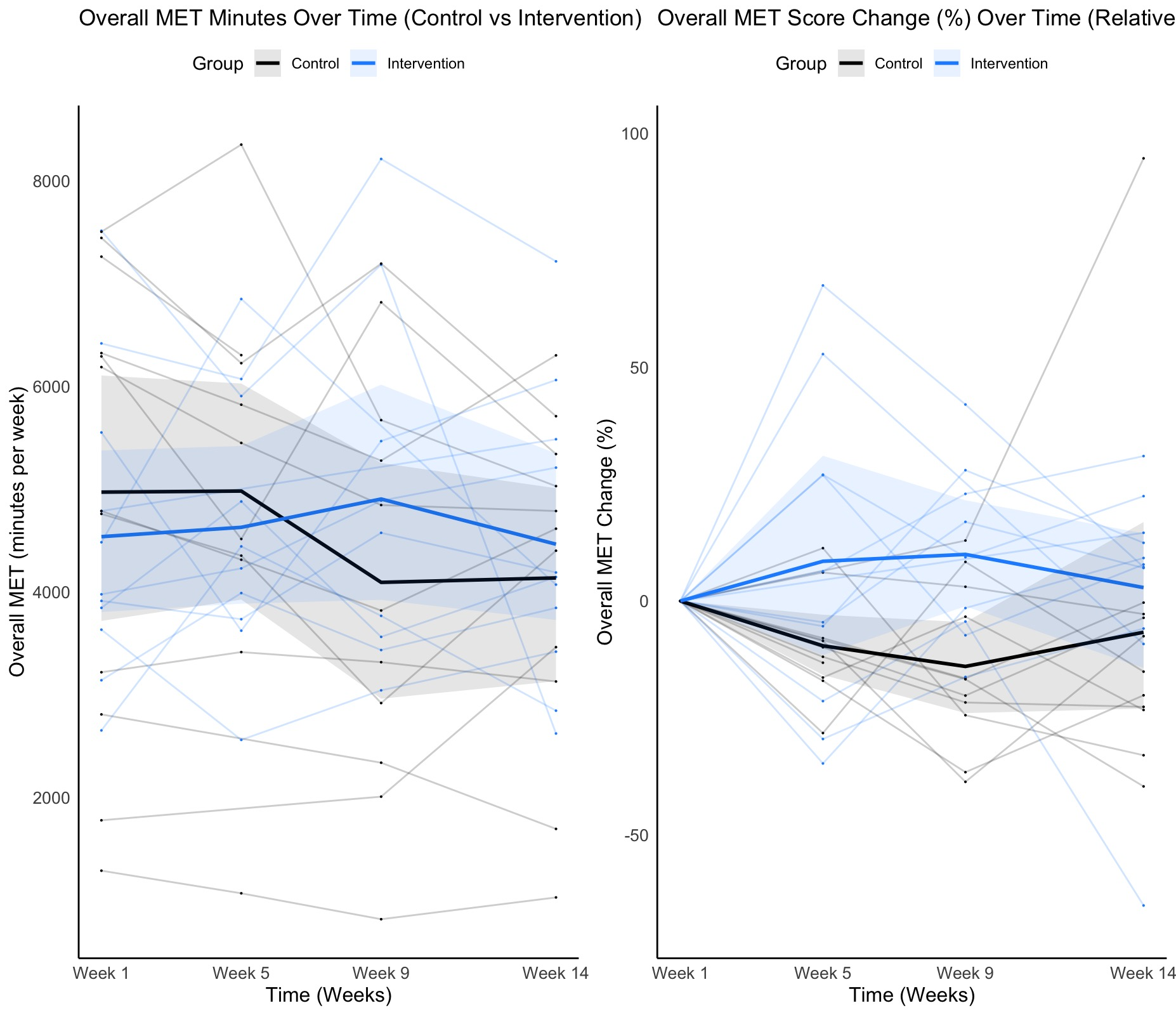


Standard Intervention

Sleep-Enhanced Intervention


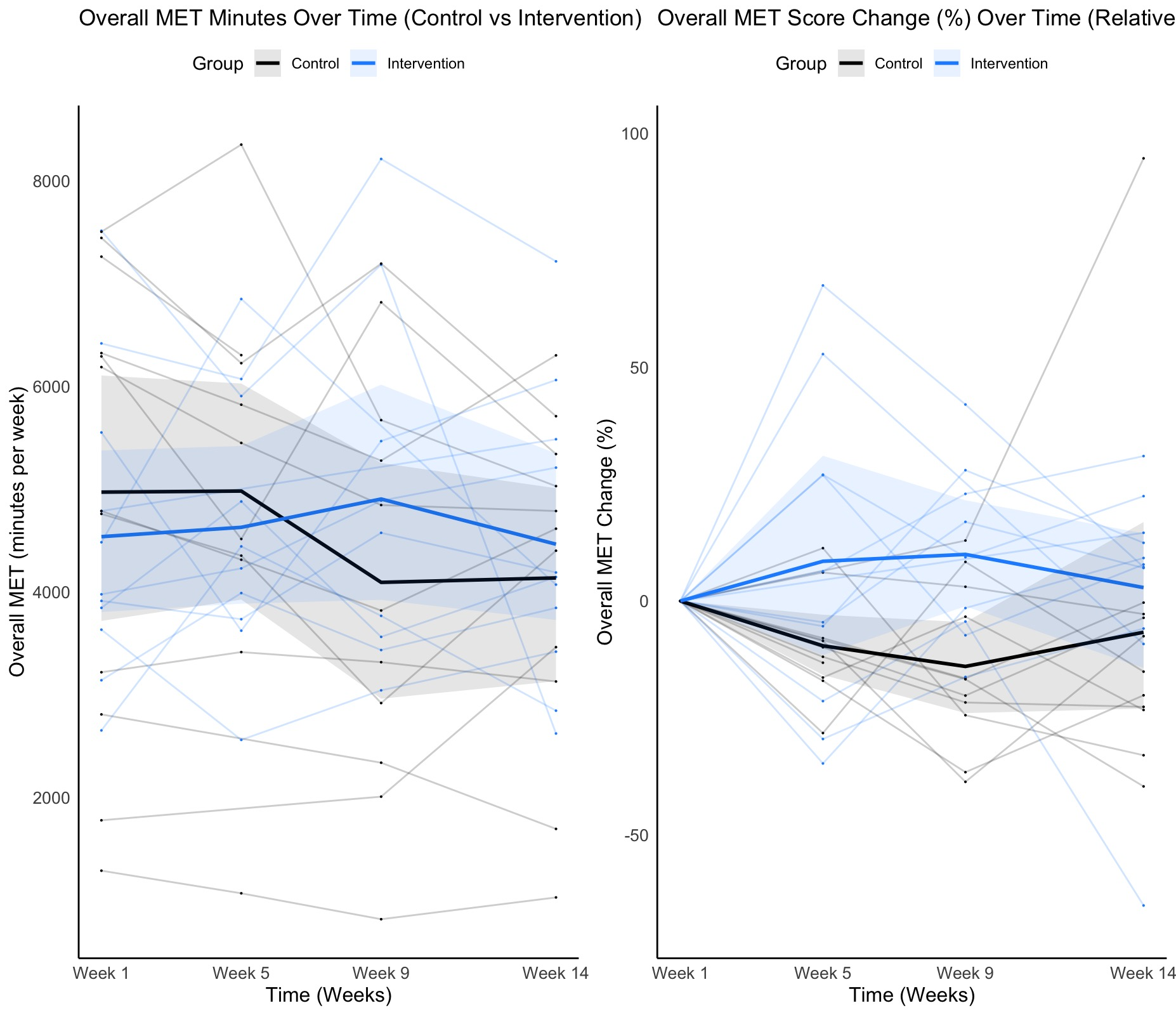


**A.**

**D.**

**C.**

**B.**

|  | Section/topic | No | CONSORT 2025 checklist item description | Reported on page no. |
| --- | --- | --- | --- | --- |
|  | **Title and abstract** | | |  |
|  | Title and structured abstract | 1a | Identification as a randomised trial | Main manuscript: (MM) Page 1; Page 3 |
|  |  | 1b | Structured summary of the trial design, methods, results, and conclusions | MM Page 1 |
|  | **Open science** | | |  |
|  | Trial registration | 2 | Name of trial registry, identifying number (with URL) and date of registration | MM Page 4 |
|  | Protocol and statistical analysis plan | 3 | Where the trial protocol and statistical analysis plan can be accessed | The trial protocol and statistical analysis plan are available upon request. Please contact the corresponding authors: Dr Rachel Gibson, email: [rachel.gibson@kcl.ac.uk](mailto:rachel.gibson@kcl.ac.uk); or Dr. Andrea Du Preez, email: [andrea.du_preez@kcl.ac.uk](mailto:andrea.du_preez@kcl.ac.uk). |
|  | Data sharing | 4 | Where and how the individual de-identified participant data (including data dictionary), statistical code and any other materials can be accessed | The individual de-identified participant data, statistical code, and other materials are available upon request. Please contact the corresponding authors: Dr Rachel Gibson, email: [rachel.gibson@kcl.ac.uk](mailto:rachel.gibson@kcl.ac.uk); or Dr. Andrea Du Preez, email: [andrea.du_preez@kcl.ac.uk](mailto:andrea.du_preez@kcl.ac.uk). |
|  | Funding and conflicts of interest | 5a | Sources of funding and other support (eg, supply of drugs), and role of funders in the design, conduct, analysis and reporting of the trial | MM Page 21 |
|  |  | 5b | Financial and other conflicts of interest of the manuscript authors | MM Page 21 |
|  | **Introduction** | | |  |
|  | Background and rationale | 6 | Scientific background and rationale | MM Pages 2-3 |
|  | Objectives | 7 | Specific objectives related to benefits and harms | MM Page 3 |
|  | **Methods** | | |  |
|  | Patient and public involvement | 8 | Details of patient or public involvement in the design, conduct and reporting of the trial | N/A |
|  | Trial design | 9 | Description of trial design including type of trial (eg, parallel group, crossover), allocation ratio, and framework (eg, superiority, equivalence, non-inferiority, exploratory) | MM Page 3-5 |
|  | Changes to trial protocol | 10 | Important changes to the trial after it commenced including any outcomes or analyses that were not prespecified, with reason | N/A |
|  | Trial setting | 11 | Settings (eg, community, hospital) and locations (eg, countries, sites) where the trial was conducted | MM Pages 4 |
|  | Eligibility criteria | 12a | Eligibility criteria for participants | MM Pages 4 |
|  |  | 12b | If applicable, eligibility criteria for sites and for individuals delivering the interventions (eg, surgeons, physiotherapists) | N/A |
|  | Intervention and comparator | 13 | Intervention and comparator with sufficient details to allow replication. If relevant, where additional materials describing the intervention and comparator (eg, intervention manual) can be accessed | MM Pages 5-9 Supplementary Materials (SM) Pages 2-14; Study Protocol available upon request. |
|  | Outcomes | 14 | Prespecified primary and secondary outcomes, including the specific measurement variable (eg, systolic blood pressure), analysis metric (eg, change from baseline, final value, time to event), method of aggregation (eg, median, proportion), and time point for each outcome | MM Pages 7-10; SM Pages 2-14 |
|  | Harms | 15 | How harms were defined and assessed (eg, systematically, non-systematically) | MM Page 7-9 |
|  | Sample size | 16a | How sample size was determined, including all assumptions supporting the sample size calculation | MM Pages 4-5 |
|  |  | 16b | Explanation of any interim analyses and stopping guidelines | MM Page 3-4 |
|  | Randomisation: |  |  |  |
|  | Sequence generation | 17a | Who generated the random allocation sequence and the method used | MM Page 5 |
|  |  | 17b | Type of randomisation and details of any restriction (eg, stratification, blocking and block size) | MM Page 5 |
|  |  |  |  |  |
|  | Allocation concealment mechanism | 18 | Mechanism used to implement the random allocation sequence (eg, central computer/telephone; sequentially numbered, opaque, sealed containers), describing any steps to conceal the sequence until interventions were assigned | MM Page 5 |
|  | Implementation | 19 | Whether the personnel who enrolled and those who assigned participants to the interventions had access to the random allocation sequence | MM Page 5 |
|  | Blinding | 20a | Who was blinded after assignment to interventions (eg, participants, care providers, outcome assessors, data analysts) | MM Page 5 |
|  |  | 20b | If blinded, how blinding was achieved and description of the similarity of interventions | Page 5 |
|  | Statistical methods | 21a | Statistical methods used to compare groups for primary and secondary outcomes, including harms | MM Pages 11-12 & SM Page 12-15 |
|  |  | 21b | Definition of who is included in each analysis (eg, all randomised participants), and in which group | MM Pages 11-12 & SM Page 12-15 |
|  |  | 21c | How missing data were handled in the analysis | MM Pages 11-12 & SM Page 12-15 |
|  |  | 21d | Methods for any additional analyses (eg, subgroup and sensitivity analyses), distinguishing prespecified from post hoc | MM Pages 11-12 & SM Page 12-15 |
|  | **Results** | | |  |
|  | Participant flow, including flow diagram | 22a | For each group, the numbers of participants who were randomly assigned, received intended intervention, and were analysed for the primary outcome | MM Figure 2 |
|  |  | 22b | For each group, losses and exclusions after randomisation, together with reasons | MM Figure 2 |
|  | Recruitment | 23a | Dates defining the periods of recruitment and follow-up for outcomes of benefits and harms | MM Page 3 & Figure 1 |
|  |  | 23b | If relevant, why the trial ended or was stopped | MM Page 3 |
|  | Intervention and comparator delivery | 24a | Intervention and comparator as they were actually administered (eg, where appropriate, who delivered the intervention/comparator, how participants adhered, whether they were delivered as intended (fidelity)) | MM Pages 5-9 |
|  |  | 24b | Concomitant care received during the trial for each group | N/A |
|  | Baseline data | 25 | A table showing baseline demographic and clinical characteristics for each group | MM Table 1 & SM Table 1 |
|  | Numbers analysed,  outcomes and estimation | 26 | For each primary and secondary outcome, by group:  ● the number of participants included in the analysis  ● the number of participants with available data at the outcome time point  ● result for each group, and the estimated effect size and its precision (such as 95% confidence interval)  ● for binary outcomes, presentation of both absolute and relative effect size | MM Pages 12-15; MM Tables 2-4; MM Figures 4-8 SM Pages 15-19; SM Tables 1-8 and SM Figure 1 |
|  | Harms | 27 | All harms or unintended events in each group | N/A |
|  | Ancillary analyses | 28 | Any other analyses performed, including subgroup and sensitivity analyses, distinguishing pre-specified from post hoc | MM Figures 4-8, SM Tables 1-8 and SM Figure 1 |
|  | **Discussion** | | |  |
|  | Interpretation | 29 | Interpretation consistent with results, balancing benefits and harms, and considering other relevant evidence | MM Pages 17-21 |
|  | Limitations | 30 | Trial limitations, addressing sources of potential bias, imprecision, generalisability, and, if relevant, multiplicity of analyses | MM Pages 19-21 |

Citation: Hopewell S, Chan AW, Collins GS, Hróbjartsson A, Moher D, Schulz KF, et al. CONSORT 2025 Statement: updated guideline for reporting randomised trials. BMJ. 2025; 388:e081123. <https://dx.doi.org/10.1136/bmj-2024-081123>
© 2025 Hopewell et al. This is an Open Access article distributed under the terms of the Creative Commons Attribution License (<https://creativecommons.org/licenses/by/4.0/>), which permits unrestricted use, distribution, and reproduction in any medium, provided the original work is properly cited.

*We strongly recommend reading this statement in conjunction with the CONSORT 2025 Explanation and Elaboration and/or the CONSORT 2025 Expanded Checklist for important clarifications on all the items. We also recommend reading relevant CONSORT extensions. See [www.consort-spirit.org](http://www.consort-spirit.org).

**
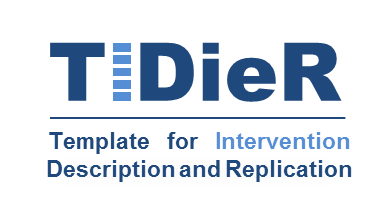
The TIDieR (Template for Intervention Description and Replication) Checklist*:**

Information to include when describing an intervention and the location of the information

| **Item number** | **Item** | **Where located **** | |
| --- | --- | --- | --- |
|  |  | Primary paper  (Page or appendix  number) | Other ^†^ (details) |
|  | **BRIEF NAME** |  |  |
| **1.** | Provide the name or a phrase that describes the intervention. | Main manuscript (MM) Page 1 & Page 3 | ___________ |
|  | **WHY** |  |  |
| **2.** | Describe any rationale, theory, or goal of the elements essential to the intervention. | MM Pages 2-3 | ___________ |
|  | **WHAT** |  |  |
| **3.** | Materials: Describe any physical or informational materials used in the intervention, including those provided to participants or used in intervention delivery or in training of intervention providers. Provide information on where the materials can be accessed (e.g. online appendix, URL). | MM Pages 5-9 & Supplementary Materials (SM) Pages 2-14 | ___________ |
| **4.** | Procedures: Describe each of the procedures, activities, and/or processes used in the intervention, including any enabling or support activities. | MM Pages 5-9 & SM Pages 2-14 | ___________ |
|  | **WHO PROVIDED** |  |  |
| **5.** | For each category of intervention provider (e.g. psychologist, nursing assistant), describe their expertise, background and any specific training given. | MM Page 5-9 | ___________ |
|  | **HOW** |  |  |
| **6.** | Describe the modes of delivery (e.g. face-to-face or by some other mechanism, such as internet or telephone) of the intervention and whether it was provided individually or in a group. | MM Pages 5-9, MM Figure 1 & SM Pages 2-14 | ___________ |
|  | **WHERE** |  |  |
| **7.** | Describe the type(s) of location(s) where the intervention occurred, including any necessary infrastructure or relevant features. | MM Pages 3-4, MM Figure 1 & SM Pages 2-14 | ___________ |
|  | **WHEN and HOW MUCH** |  |  |
| **8.** | Describe the number of times the intervention was delivered and over what period of time including the number of sessions, their schedule, and their duration, intensity or dose. | MM Pages 5-9, MM Figure 1 & SM Pages 2-14 | ___________ |
|  | **TAILORING** |  |  |
| **9.** | If the intervention was planned to be personalised, titrated or adapted, then describe what, why, when, and how. | MM Pages 5-9 & SM Pages 2-14 | ___________ |
|  | **MODIFICATIONS** |  |  |
| **10.^ǂ^** | If the intervention was modified during the course of the study, describe the changes (what, why, when, and how). | N/A | ___________ |
|  | **HOW WELL** |  |  |
| **11.** | Planned: If intervention adherence or fidelity was assessed, describe how and by whom, and if any strategies were used to maintain or improve fidelity, describe them. | N/A | Outlined in Statistical Analysis Plan – available upon request. Please contact the corresponding authors: Dr Rachel Gibson, email: [rachel.gibson@kcl.ac.uk](mailto:rachel.gibson@kcl.ac.uk); or Dr. Andrea Du Preez, email: [andrea.du_preez@kcl.ac.uk](mailto:andrea.du_preez@kcl.ac.uk). |
| **12.^ǂ^** | Actual: If intervention adherence or fidelity was assessed, describe the extent to which the intervention was delivered as planned. | MM Page 9-12 & SM Pages 12-15 | ___________ |

** **Authors** - use N/A if an item is not applicable for the intervention being described. **Reviewers** – use ‘?’ if information about the element is not reported/not sufficiently reported.

† If the information is not provided in the primary paper, give details of where this information is available. This may include locations such as a published protocol or other published papers (provide citation details) or a website (provide the URL).

ǂ If completing the TIDieR checklist for a protocol, these items are not relevant to the protocol and cannot be described until the study is complete.

* We strongly recommend using this checklist in conjunction with the TIDieR guide (see *BMJ* 2014;348:g1687) which contains an explanation and elaboration for each item.

* The focus of TIDieR is on reporting details of the intervention elements (and where relevant, comparison elements) of a study. Other elements and methodological features of studies are covered by other reporting statements and checklists and have not been duplicated as part of the TIDieR checklist. When a **randomised trial** is being reported, the TIDieR checklist should be used in conjunction with the CONSORT statement (see [www.consort-statement.org](http://www.consort-statement.org)) as an extension of **Item 5 of the CONSORT 2010 Statement.** When a **clinical trial** **protocol** is being reported, the TIDieR checklist should be used in conjunction with the SPIRIT statement as an extension of **Item 11 of the SPIRIT 2013 Statement** (see [www.spirit-statement.org](http://www.spirit-statement.org)). For alternate study designs, TIDieR can be used in conjunction with the appropriate checklist for that study design (see [www.equator-network.org](http://www.equator-network.org)).

**REFERENCES**

1. Mohideen, A., Bouvin, C., Judah, G., Picariello, F. & Gardner, B. Feasibility and acceptability of a personalised script-elicitation method for improving evening sleep hygiene habits. *Health Psychol Behav Med* 11, 2162904 (2023).

2. Horne, J. A. & Ostberg, O. A self-assessment questionnaire to determine morningness-eveningness in human circadian rhythms. *Int J Chronobiol* 4, 97–110 (1976).

3. Saunders, J. B., Aasland, O. G., Babor, T. F., De la Fuente, J. R. & Grant, M. Development of the alcohol use disorders identification test (AUDIT): WHO collaborative project on early detection of persons with harmful alcohol consumption‐II. *Addiction* 88, 791–804 (1993).

4. Craig, C. L. *et al.* International physical activity questionnaire: 12-country reliability and validity. *Med Sci Sports Exerc* 35, 1381–1395 (2003).

5. Buysse, D. J. *et al.* Development and validation of patient-reported outcome measures for sleep disturbance and sleep-related impairments. *Sleep* 33, 781–792 (2010).

6. Johns, M. W. A new method for measuring daytime sleepiness: the Epworth sleepiness scale. *Sleep* 14, 540–545 (1991).

7. Mastin, D. F., Bryson, J. & Corwyn, R. Assessment of sleep hygiene using the Sleep Hygiene Index. *J Behav Med* 29, 223–227 (2006).

8. Spitzer, R. L., Kroenke, K., Williams, J. B. W. & Löwe, B. A Brief Measure for Assessing Generalized Anxiety Disorder: The GAD-7. *Arch Intern Med* 166, 1092–1097 (2006).

9. Kroenke, K., Spitzer, R. L. & Williams, J. B. W. The PHQ‐9: validity of a brief depression severity measure. *J Gen Intern Med* 16, 606–613 (2001).

10. Schaufeli, W. B., Salanova, M., González-Romá, V. & Bakker, A. B. The measurement of engagement and burnout: A two sample confirmatory factor analytic approach. *J Happiness Stud* 3, 71–92 (2002).

11. Judge, T. A., Bono, J. E. & Locke, E. A. Personality and job satisfaction: the mediating role of job characteristics. *Journal of applied psychology* 85, 237 (2000).

12. Lindner, C., Lindner, M. A. & Retelsdorf, J. Measuring self-control depletion in achievement situations: A validation of the 5-item brief state self-control capacity scale. *Diagnostica* 65, 228–242 (2019).

13. Reis, D., Xanthopoulou, D. & Tsaousis, I. Measuring job and academic burnout with the Oldenburg Burnout Inventory (OLBI): Factorial invariance across samples and countries. *Burn Res* 2, 8–18 (2015).

14. R Core Team. R: A language and environment for statistical computing. R Foundation for Statistical Computing Preprint at (2017).

15. Ainsworth, B. E. *et al.* 2011 Compendium of Physical Activities: a second update of codes and MET values. *Med Sci Sports Exerc* 43, 1575–1581 (2011).

16. Health England, P. *A Quick Guide to the Government’s Healthy Eating Recommendations*. www.facebook.com/PublicHealthEngland (2018).

17. Panizza, C. E. *et al.* Testing the Predictive Validity of the Healthy Eating Index-2015 in the Multiethnic Cohort: Is the Score Associated with a Reduced Risk of All-Cause and Cause-Specific Mortality? *Nutrients* 10, (2018).

18. Health England, P. *Government Dietary Recommendations Government Recommendations for Energy and Nutrients for Males and Females Aged 1-18 Years and 19+ Years*. www.gov.uk/phe (2016).

19. Rathnayake, K. M., Madushani, P. A. E. & Silva, K. Use of dietary diversity score as a proxy indicator of nutrient adequacy of rural elderly people in Sri Lanka. *BMC Res Notes* 5, 469 (2012).

20. Landry, G. J., Best, J. R. & Liu-Ambrose, T. Measuring sleep quality in older adults: a comparison using subjective and objective methods. *Front Aging Neurosci* 7, (2015).

21. Elbaz, M., Yauy, K., Metlaine, A., Martoni, M. & Leger, D. Validation of a new actigraph motion watch versus polysomnography on 70 healthy and suspected sleepdisordered subjects. *J Sleep Res* 21, 218 (2012).

22. Wittmann, M., Dinich, J., Merrow, M. & Roenneberg, T. Social Jetlag: Misalignment of Biological and Social Time. *Chronobiol Int* 23, 497–509 (2006).

23. Cespedes Feliciano, E. M. *et al.* Chronotype, Social Jet Lag, and Cardiometabolic Risk Factors in Early Adolescence. *JAMA Pediatr* 173, 1049–1057 (2019).

24. Okajima, I., Komada, Y., Ito, W. & Inoue, Y. Sleep Debt and Social Jetlag Associated with Sleepiness, Mood, and Work Performance among Workers in Japan. *Int J Environ Res Public Health* 18, (2021).
