# Supplementary material for: Randomised feasibility trial of a remotely delivered holistic UK employee programme combining tailored sleep hygiene, diet, and physical activity counselling for weight management: a mixed-methods evaluation
Source: J Nutr Sci. 2026 Jun 18;15:e45. doi: 10.1017/jns.2026.10104 (PMC13279985; doi:10.1017/jns.2026.10104)
Supplement: Du Preez et al. supplementary material 2 — Du Preez et al. supplementary material [file S2048679026101049sup002.docx]

**SUPPLEMENTARY MATERIALS**

**Randomised feasibility trial of a remotely delivered holistic UK employee programme combining tailored sleep hygiene, diet, and physical activity counselling for weight management: a mixed-methods evaluation**

Andrea Du Preez^1*,^ Danae Marshall^1^*, Lorraine Kelly^2^, Kirti Swift^2^, Zak Evans^3^, Michael Clinton^3^, Rakhee Doshi^4^, Charlotte Fitzhugh^5^, Benjamin Gardner^4^, Rachel Gibson^1^* and Wendy Hall^1^*

(1). Department of Nutritional Sciences, School of Life Course & Population Sciences, Faculty of Life Sciences & Medicine, King’s College London, 150 Stamford Street, London SE1 9NH, UK; (2). Organisational Development, King's College London, 5-11 Lavington Street, SE1 0NZ, UK; (3). Department of Human Resource Management & Employment Relations, King’s Business School, Bush House, 30 Aldwych, London WC2B 4BG, UK; (4). Research Institute for Sport and Exercise Sciences, Liverpool John Moores University, Liverpool, UK; (5). School of Psychological Sciences, University of Surrey, Guildford, GU2 7XH, UK.

***Authors contributed equally to the study and manuscript.**

**Keywords:** Feasibility trial, holistic, lifestyle intervention, weight management, sleep, diet, physical activity.

**Correspondence to:** Dr Rachel Gibson, email: [rachel.gibson@kcl.ac.uk](mailto:rachel.gibson@kcl.ac.uk); or Dr. Andrea Du Preez, email: [andrea.du_preez@kcl.ac.uk](mailto:andrea.du_preez@kcl.ac.uk). Franklin Wilkins Building, 150 Stamford Street, London SE1 9NH, UK.

**CONTENT**

**Sleep Hygiene Guidelines**

**The KING’S-WHOLE Study**

**
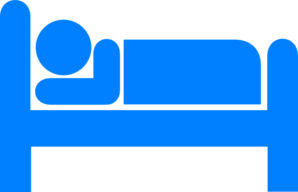
**

**Sleep Hygiene**

**Guidelines for Better Sleep**

**The mystery of sleep**

It’s where our brains travel to every night, it’s out of our voluntary control, and we often complain that we don’t get enough of it. Sleep is the mysterious shift in consciousness that our bodies require every day. It’s vital for our health and wellbeing but its importance goes far beyond that, sleep is something that we can’t live without. Not only do we function worse when we don’t get enough quality sleep, but it can lead to long-term health problems. That’s why if we’re having sleep problems it’s important we do all that we can to rectify these difficulties and restore quality sleep into our lifestyles.

**Trouble sleeping?**

If you’re experiencing sleep problems, you’re not alone. A 2012 survey by Premier Inn found that money worries are keeping 37% of us awake at night, a 2011 survey by IKEA and Which? Found that 7 in 10 feel they aren’t getting enough sleep, and the NHS spends over £50 million on sleeping pills alone (The Independent, 2012). But there’s no need to suffer in silence if you are experiencing sleeping difficulties, there is plenty that you can do to restore quality sleep into your life.

**How much sleep do you need?**

On average most adults seem to need around 7-9 hours of sleep per night though this can vary from person to person. As we grow older our sleep patterns change and as we go through our life stages we tend to get less and less sleep. Older adults tend to sleep less, not because they need less sleep, but because we’re more likely to experience disturbed sleep as we grow older. On average:

»»New-born babies sleep up to 18-21 hours per day

»»3-5 year olds sleep 11-13 hours per day

»»Pre-teens need 11 hours

»»Teenagers need 9-11 hours

»»Adults aged 18-64 need 7-9 hours

»»Adults aged 64+ need 7-8 hours

**Sleep Hygiene**

Sleep hygiene is a term used to describe behaviours that are conducive to having normal, quality night-time sleep and full daytime alertness. These behaviours are specific, modifiable and controllable. As part of the KING’S-WHOLE study we aim to work with you to identify habits and practices that are relevant and realistic targets in terms of your lifestyle, in order to improve your sleep duration and quality.

**Targets for better sleep - What is relevant to you?**

- Sleep-hygienic bedroom environment

| Target | Tips |
| --- | --- |
| Make the room dark | Use black-out curtains or an eye mask |
| Find the ideal temperature | Slightly cool – 16-18°C |
| Keep it quiet | Use ear plugs if needed and close windows if near busy roads |
| Move the clutter | Keep clothes, shoes, books etc out of sight – use wardrobes, laundry basket, bookcases |
| Make your bed comfortable | Invest in soft and comfortable linens, a comfortable bed and mattress |
| Remove computers or TVs | Remove stimulating screen devices from the bedroom |
| Banish the backlit devices | Turn phones on to ‘do not disturb’ or silent mode. Leave non-essential devices outside the bedroom.  Keep all devices out of arm’s reach |
| Keep bedroom interior décor calming | Avoid bright colours, as they can be stimulating. Decorate your room with photographs, artwork, and plants that make you feel relaxed and calm. |
| Use the bedroom for bedtime | Avoid treating your bedroom as an extension of your living room or study – use for sleep only. |
| Use relaxing scents | Certain smells can help you feel relaxed and calm; essential oils of lavender, sprinkle pot pourri, use a diffuser of lavender or geranium. Fresh sheets and smell of fabric softener promote sleepiness. |

- Sleep hygiene

| Target | Tips |
| --- | --- |
| Reduce the light | Reduce light in the evening throughout the house. Use dimmers if you have them, or lamps with low wattage bulbs. |
| Stick to a routine | Routines help to maintain regular sleep – aim to go to bed and wake up at the same time each day. |
| Let the morning light in | Expose yourself to sunlight/bright light early in the morning. |
| Reduce night-time awakening | Minimise disruptions by emptying your bladder before going to bed, turning your phone to silent, and ensuring you cannot be disturbed by pets. |
| Avoid using technology before bed | Avoid using in the hours before bedtime e.g., computers, mobile phones, TV. |
| Avoid daytime napping | If you feel tired during the day, try taking a brief walking break. |
| Avoid nicotine | Avoid, especially 4-6 hours before bedtime. |


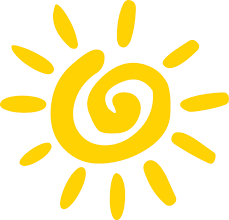


- Diet

| Target | Condition |
| --- | --- |
| Avoid late or heavy meals | Avoid having dinner past 8pm, try not to go to bed on a very full stomach. |
| Avoid going to bed too hungry | Avoid by having a small snack based on wholegrains, a banana, yoghurt or a milk-based drink (not high in sugar). |
| No heavy, spicy, or sugary foods before bed | Avoid 4-6 hours before bedtime |
| Avoid alcohol/caffeine before bed | Avoid consuming within 6hr of sleep |


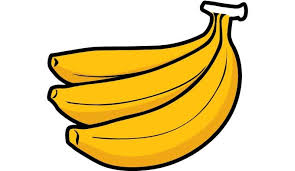


- Exercise

| Target | Ideal conditions |
| --- | --- |
| Get regular exercise | Maintaining an exercise routine can help you enjoy better quality sleep |
| Avoid strenuous exercise before bed | Strenuous exercise should be avoided 2 hours before bedtime. |
| Regular low-intensity exercise | Regular low intensity exercise in the evening can promote muscle relaxation and improve sleep quality e.g., yoga, Pilates. |


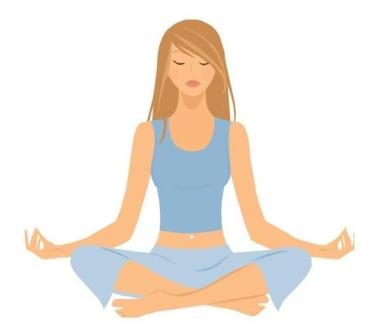


- Stress and worry

| Target | Ideal conditions |
| --- | --- |
| Reduce anxiety and stress with deep breathing exercises | Practice deep breathing exercises to slow your heart rate and calm yourself. |
| Leave your worries behind | Avoid taking them to bed, deal with them during the day e.g., write a list, schedule “worry time” in the day to think about any nagging tasks and face them. |


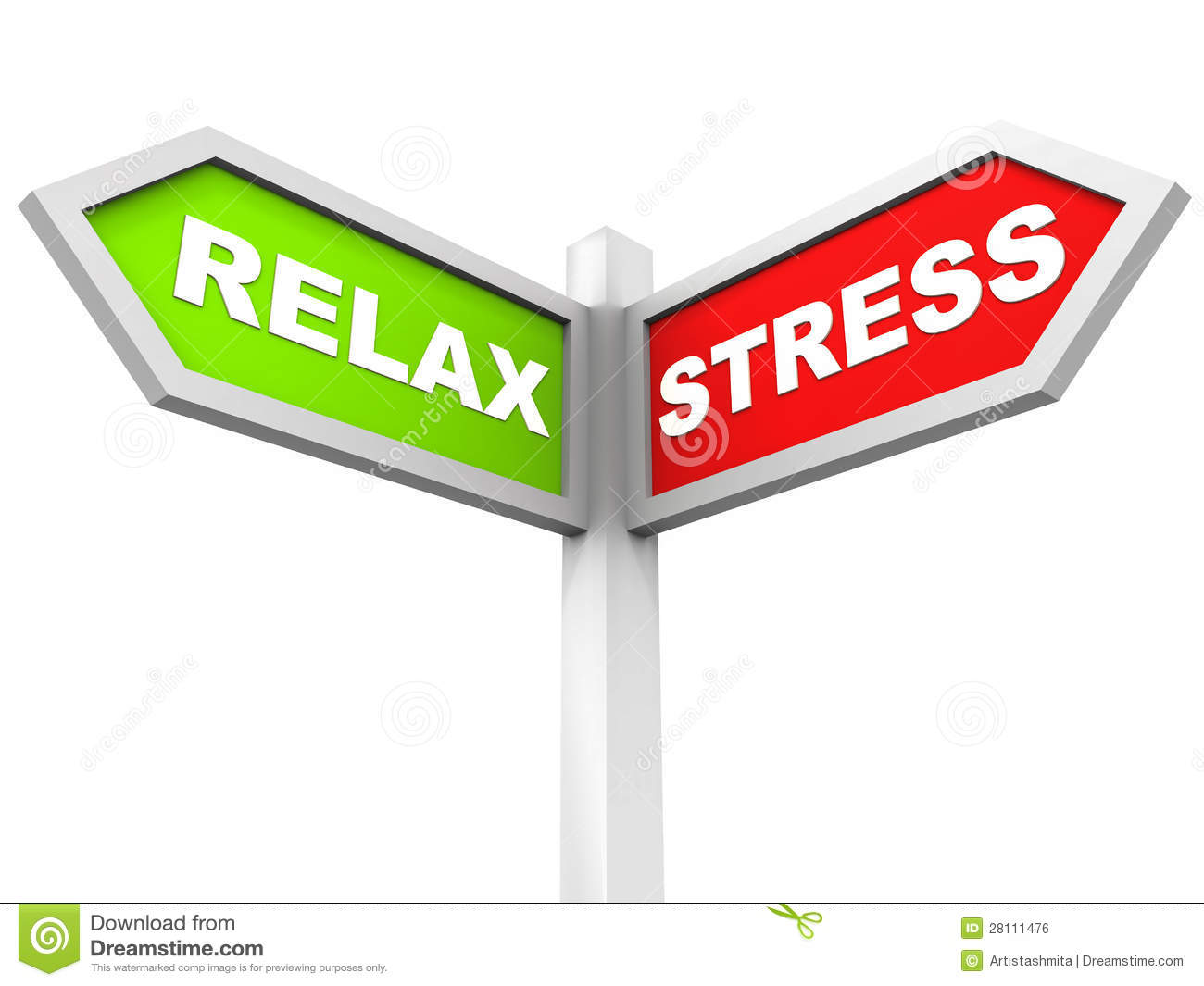


- Relaxation

| Target | Ideal conditions |
| --- | --- |
| Practice restful activities | Set aside time to unwind before bed. Incorporating activities into your bedtime routine like deep breathing, warm baths, or progressive muscle relaxation* can help you relax |
| Have restful thoughts | Visualize a peaceful, restful place: close your eyes and imagine a peaceful, calming place, and concentrate on how this makes you feel |
| Mindfulness | Using mobile apps such as Mindfulness, Calm, Insight timer (free), Headspace, Simple habit, Ten Percent Happier Meditation, and Buddify during the day may promote relaxation and sleep quality. |

* Progressive muscle relaxation: start by tensing your toes as tightly as you can then completely relax, next tense the muscles in your calves and release. Continue with each muscle group working your way to the top of your head.

**Thank you for taking part**

Remember everyone is different and not all of these targets will be effective or achievable in your lifestyle. We are here to help; please do not hesitate to contact us should you have any further questions. Sleep well,

The KING’S-WHOLE Team
