# Supplementary material for: Randomised feasibility trial of a remotely delivered holistic UK employee programme combining tailored sleep hygiene, diet, and physical activity counselling for weight management: a mixed-methods evaluation
Source: J Nutr Sci. 2026 Jun 18;15:e45. doi: 10.1017/jns.2026.10104 (PMC13279985; doi:10.1017/jns.2026.10104)
Supplement: Du Preez et al. supplementary material 3 — Du Preez et al. supplementary material [file S2048679026101049sup003.docx]

**SUPPLEMENTARY MATERIALS**

**Randomised feasibility trial of a remotely delivered holistic UK employee programme combining tailored sleep hygiene, diet, and physical activity counselling for weight management: a mixed-methods evaluation**

Andrea Du Preez^1*,^ Danae Marshall^1^*, Lorraine Kelly^2^, Kirti Swift^2^, Zak Evans^3^, Michael Clinton^3^, Rakhee Doshi^4^, Charlotte Fitzhugh^5^, Benjamin Gardner^4^, Rachel Gibson^1^* and Wendy Hall^1^*

(1). Department of Nutritional Sciences, School of Life Course & Population Sciences, Faculty of Life Sciences & Medicine, King’s College London, 150 Stamford Street, London SE1 9NH, UK; (2). Organisational Development, King's College London, 5-11 Lavington Street, SE1 0NZ, UK; (3). Department of Human Resource Management & Employment Relations, King’s Business School, Bush House, 30 Aldwych, London WC2B 4BG, UK; (4). Research Institute for Sport and Exercise Sciences, Liverpool John Moores University, Liverpool, UK; (5). School of Psychological Sciences, University of Surrey, Guildford, GU2 7XH, UK.

***Authors contributed equally to the study and manuscript.**

**Keywords:** Feasibility trial, holistic, lifestyle intervention, weight management, sleep, diet, physical activity.

**Correspondence to:** Dr Rachel Gibson, email: [rachel.gibson@kcl.ac.uk](mailto:rachel.gibson@kcl.ac.uk); or Dr. Andrea Du Preez, email: [andrea.du_preez@kcl.ac.uk](mailto:andrea.du_preez@kcl.ac.uk). Franklin Wilkins Building, 150 Stamford Street, London SE1 9NH, UK.

**CONTENT**

**Sleep Hygiene Guidelines**

## KING’s-WHOLE Trial: Sample Coding Manual

| Code | | Description | Positive Example | | Negative Example | Exemptions/restrictions |
| --- | --- | --- | --- | --- | --- | --- |
| Motivations to participate-seeking support  from professional with expert knowledge | This includes the motivational drives of wanting to participate | | “That's what really attracted me to this study because it was more about the eating habits, because I knew I could do the exercise cause I've done it before, but I needed an entire life change.” (P2) | “But during that, I found that I lost quite a bit of weight. I found that I got very, very engaged and exercise.” (P2) | | This does not include discussion about the motivational drives rather recalls of previous health behaviour change attempts |
| Psychological benefits-adopting a less rigid approach to behaviour change | | This includes the perceived psychological benefits of the intervention | Being a little bit less strict with myself. So being a little bit lenient that you know, obviously you're gonna have some days that don't go well.” (P2) | | “It's kind of about workload really. So, it depends if I was really busy then you know.” (P6) | This does not include talk of adopting a less rigid schedule rather busy workload impacting adherence |
| Factors in adhering/implementing behaviour changes perceived to be non-disruptive (i.e., can be incorporated into everyday routines) | | This includes factors of adherence to the intervention | “Super easy didn't affect me in any way, shape, or form. Very straightforward. That did not change very much. All I did is adjust the quantity.” (P3) | | “I don't think it's changing my behaviour. I never abused myself that much.” (P3) | This does not include discussion pertaining to adherence factors |
| Psychological benefits: validation from group sessions | | This includes the perceived psychological benefits of the peer support sessions | “It's just nice to speak to somebody else who's who, who sort of going, you know, experiencing the same things, who, who also likes to eat their food and you know.” (P8) | | “It's kind of trying to find a routine that works for me around that. The fact that I have three kids and you know, I don't think the other, the other guy from the from the group…It wasn’t super helpful” (P7) | This does not include talk of the benefits of the peer sessions |

*Note.* P, Participant.
